# The origin and early evolution of metatherian mammals: the Cretaceous record

**DOI:** 10.3897/zookeys.465.8178

**Published:** 2014-12-17

**Authors:** Thomas E. Williamson, Stephen L. Brusatte, Gregory P. Wilson

**Affiliations:** 1 New Mexico Museum of Natural History and Science, 1801 Mountain Road, NW, Albuquerque, New Mexico 87104-1375, USA; 2 School of GeoSciences, University of Edinburgh, Edinburgh EH9 3JW, UK; 3 Department of Biology and Burke Museum of Natural History and Culture, 24 Kincaid Hall, University of Washington, Seattle, Washington 98195-1800, USA

**Keywords:** Cretaceous, Metatheria, Mammalia, Boreosphenida, Deltatheroida, Marsupialiformes, dentition, osteology, phylogeny, paleobiology, macroevolution, paleoenvironment, biogeography

## Abstract

Metatherians, which comprise marsupials and their closest fossil relatives, were one of the most dominant clades of mammals during the Cretaceous and are the most diverse clade of living mammals after Placentalia. Our understanding of this group has increased greatly over the past 20 years, with the discovery of new specimens and the application of new analytical tools. Here we provide a review of the phylogenetic relationships of metatherians with respect to other mammals, discuss the taxonomic definition and diagnosis of Metatheria, outline the Cretaceous history of major metatherian clades, describe the paleobiology, biogeography, and macroevolution of Cretaceous metatherians, and provide a physical and climatic background of Cretaceous metatherian faunas. Metatherians are a clade of boreosphendian mammals that must have originated by the Late Jurassic, but the first unequivocal metatherian fossil is from the Early Cretaceous of Asia. Metatherians have the distinctive tightly interlocking occlusal molar pattern of tribosphenic mammals, but differ from Eutheria in their dental formula and tooth replacement pattern, which may be related to the metatherian reproductive process which includes an extended period of lactation followed by birth of extremely altricial young. Metatherians were widespread over Laurasia during the Cretaceous, with members present in Asia, Europe, and North America by the early Late Cretaceous. In particular, they were taxonomically and morphologically diverse and relatively abundant in the Late Cretaceous of western North America, where they have been used to examine patterns of biogeography, macroevolution, diversification, and extinction through the Late Cretaceous and across the Cretaceous-Paleogene (K-Pg) boundary. Metatherian diversification patterns suggest that they were not strongly affected by a Cretaceous Terrestrial Revolution, but they clearly underwent a severe extinction across the K-Pg boundary.

## Introduction

Metatherian mammals, which include the extant marsupials, are the second most diverse major clade of living mammals (~334 species; Armati et al. 2006). They are also an ancient group that has its origins deep in the Mesozoic Era. Today, metatherians are abundant and taxonomically and morphologically diverse in Central America, South America, and Australia, and are the dominant mammals on only one continent, Australia. However, during the Late Cretaceous, they were a diverse group on the northern continents of Laurasia. In North America, metatherians were far more numerous and taxonomically diverse than were eutherian mammals, the stem placental mammalian group that is dominant across most of the world today.

Our understanding of metatherian evolution during the Mesozoic and into the early Paleogene has grown rapidly over the past 20 years with the discovery of new fossils and the application of new analytical tools. In particular, new fossils from Asia have shed light on the origin and evolution of Theria (Eutheria + Metatheria), the earliest metatherians and the divergence between the eutherian and metatherian clades, and the early metatherian skull and postcranial skeleton (Averianov et al. 2010; Chester et al. 2010; Chester et al. 2012; Ji et al. 2002; Kielan-Jaworowska and Cifelli 2001; Lopatin and Averianov 2006; 2007; Luo et al. 2003; Luo et al. 2011; Rougier et al. 1998; Rougier et al. 2004; Szalay and Sargis 2006; Szalay and Trofimov 1996). Many new Late Cretaceous metatherian taxa and new specimens, especially from western North America, have been described, re-described, or revised (Case et al. 2005; Cifelli 2004; Cifelli and Davis in press; Cifelli and Johanson 1994; Davis 2007; Davis and Cifelli 2011; Davis et al. 2008; Fox and Naylor 2006; Hunter et al. 2010). Metatherians are now also known from the Late Cretaceous of Europe, confirmed for the first time by recent discoveries (Martin et al. 2005; Vullo et al. 2009). Many of these new discoveries have been of fairly derived metatherians somewhat closely related to marsupials, but several new basal metatherians (deltatheroidans) have also come to light recently in the Cretaceous of North America (Davis et al. 2008; Fox et al. 2007; Wilson and Riedel 2010). In sum, these discoveries make it clear that a suite of basal and more derived metatherians were widespread across Laurasia during the Cretaceous.

These discoveries have provided important new insight into the evolution and paleobiogeography of metatherians. There has been a tremendous upsurge in studies using analytical cladistic methods to clarify phylogenetic relationships within Metatheria (Davis 2007; Johanson 1996b; Luo et al. 2003; Luo et al. 2002; Rougier et al. 1998; Williamson et al. 2012), which has helped clarify the relationships and rise of Marsupialia from Cretaceous metatherians (Horovitz et al. 2008; Horovitz et al. 2009; O’Leary et al. 2013; Sánchez-Villagra et al. 2007; Wible et al. 2009; Williamson et al. 2012). Quantitative analyses have also looked at the survivorship of metatherians across the Cretaceous-Paleogene (K-Pg) boundary (Wilson 2013, 2014) and have used new morphological data to examine metatherian disparity, ecomorphological diversity, and selectivity of extinctions across the K-Pg boundary (Wilson 2013). As a result, over the past decade we have learned a tremendous deal about Cretaceous metatherians and their phylogeny and evolution.

This paper is meant to supplement the excellent review of metatherians by Kielan-Jaworowska et al. (2004, chapter 12) by summarizing developments in metatherian paleontology over the past decade, and to provide a comprehensive synopsis of Cretaceous metatherian evolution. We review the phylogenetic relationships of metatherians with respect to other mammals and the taxonomic definition and diagnosis of Metatheria, the Cretaceous history of major metatherian clades and the paleobiology and macroevolution of Cretaceous metatherians, and the physical and climatic background and biogeography of Cretaceous metatherian faunas.

## The taxonomic and evolutionary context of Metatheria

### Mammalia

**Figure 1.**
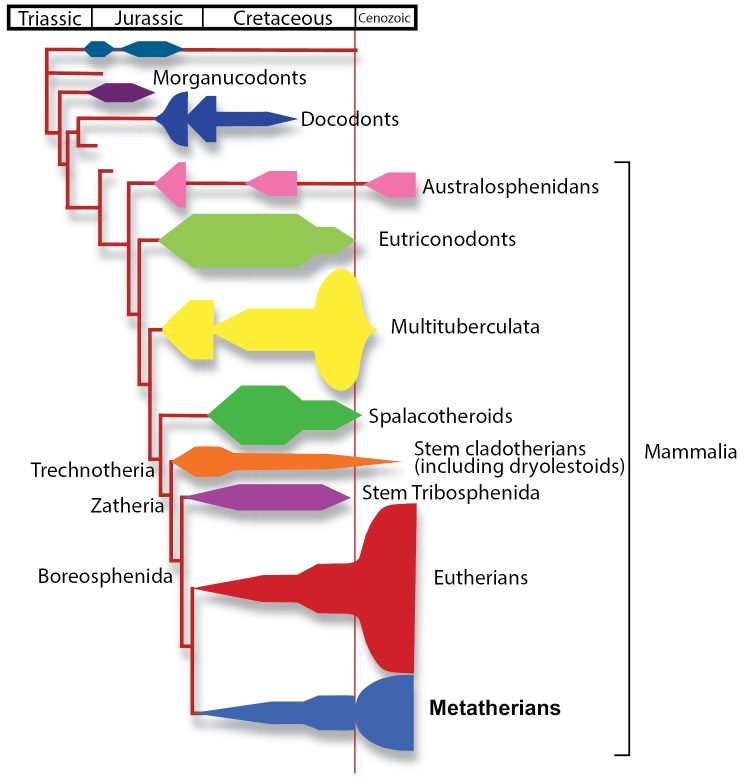
Phylogeny and diversification patterns of major mammal lineages after Luo (2007).

The taxon Mammalia consists of numerous extinct lineages as well as three extant clades: the monotremes (egg-laying mammals), marsupials (“pouched” mammals that give live birth to relatively altricial young), and placentals (mammals that give live birth to precocial young). Here, we follow the crown-group definition of Mammalia, which circumscribes the clade as the most recent common ancestor of living monotremes, marsupials, and placentals, and all its descendants (sensu Rowe 1988). Mammalia is nested within the clade Mammaliaformes (= Mammalia of Kielan-Jaworowska et al. 2004), which arose and diversified beginning in the Late Triassic, within approximately 20 million years of the Permo-Triassic mass extinction (Benton 2005; Fraser and Sues 2010) (Fig. 1). Mammaliaformes is a subset of Cynodontia, which, in turn, is a subset of Synapsida, one of the primary branches of terrestrial vertebrates that arose in the late Paleozoic. The evolution that led from pre-mammalian cynodonts to mammals is an important transition in vertebrate evolution (Kemp 2005; Kielan-Jaworowska et al. 2004; Luo 2007).

Mammaliaformes underwent multiple episodes of diversification through the Mesozoic, resulting in several now extinct lineages (Kemp 2005; Kielan-Jaworowska et al. 2004; Luo 2007). The timing of the origin of Mammalia is poorly constrained. This is largely due to the incomplete and scarce nature of mammalian fossils from the Jurassic and Early Cretaceous and to disagreements regarding the relationships among basal mammals (Cifelli and Davis 2013). There is current debate about whether mammals originated in the Late Triassic, but are poorly sampled in the fossil record at this time, or much later during the Middle Jurassic, when a diversity of unequivocal mammals first appear in the fossil record (Cifelli and Davis 2013; Zheng et al. 2013; Zhou et al. 2013). Resolving this debate in part hinges on resolving the phylogenetic positions of enigmatic Triassic–Jurassic mammaliaforms, such as the Haramiyida, which may or may not belong to crown group Mammalia. For example, if haramiyidans prove to be within the crown clade Mammalia and represent a paraphyletic group that gave rise to Multituberculata, as has been suggested by some workers (e.g., Butler 2000; Zheng et al. 2013; Bi et al. 2014), then Mammalia must have originated deep in the Triassic.

**Figure 2.**
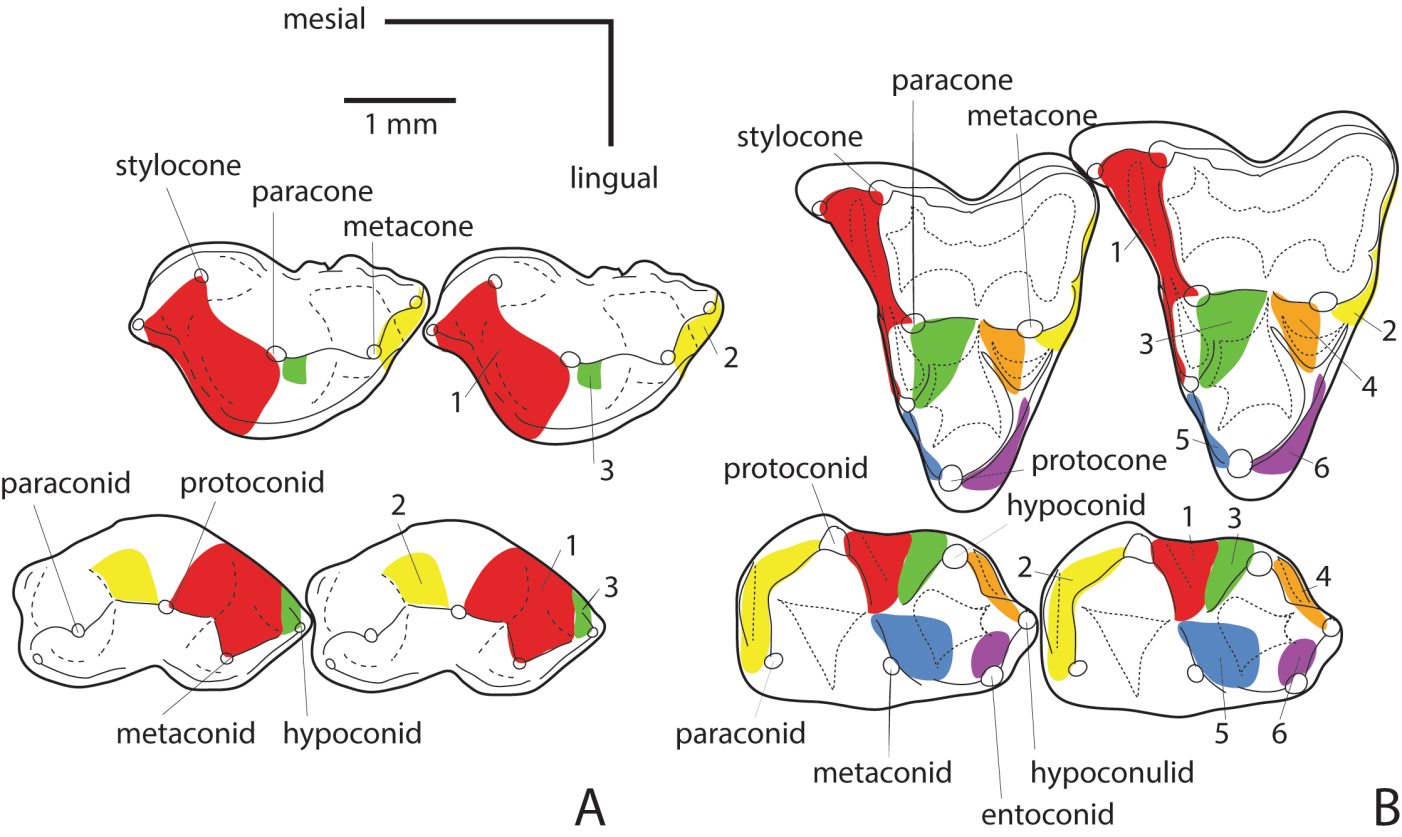
Molar terminology and wear facet designation showing upper and lower molar tooth cusp homologies between the “symmetrodont” *Kuehneotherium* (**A**) and the metatherian *Kokopellia* (**B**) after Davis (2011a).

Once Mammalia originated, several fundamental lineages split from each other during the early stages of mammalian evolution. According to several phylogenetic analyses (Kielan-Jaworowska et al. 2004; Luo et al. 2001; Luo et al. 2002; Rougier et al. 2007), monotremes may fall within Australosphenida, which is sister taxon to the Theriimorpha. The Theriimorpha later diversified into a number of now-extinct groups, such as the multituberculates and eutriconodontans, as well as the trechnotherians, which include the living therian mammals (metatherians and eutherians) and their closest fossil relatives (Averianov et al. 2013; Kielan-Jaworowska et al. 2004; Luo et al. 2007a; Luo et al. 2007b). Much of this diversification probably occurred in Asia (Averianov et al. 2013; Martin and Averianov 2010; Martin et al. 2010) before spreading to other continents during the later Jurassic, although this pattern may be a figment of the relatively complete and well-studied Asian fossil record compared to that of other regions. Trechnotherians underwent three major diversifications, all likely confined to Laurasia, before the origin of the tribosphenic molar (teeth in which a lingual upper cusp occludes into a distal basin on the lower molars; Fig. 2) within the Boreosphenida (Averianov et al. 2013). Each evolutionary radiation of trechnotherians involved successive transformation of the molar form, eventually resulting in the tribosphenic pattern found in the immediate ancestors of metatherians and eutherians (stem boreosphenidans).

### Boreosphenida

Most Mesozoic mammal fossils consist of fragmentary jaws and teeth, which largely explains the intense emphasis that paleontologists place on the evolution of the mammalian dentition. One of the most distinctive and important features that arose in some mammaliaforms is precise molar occlusion. The upper and lower molars of some basal mammaliaforms (e.g., kuehneotheriids) are nearly triangular and have a loosely interlocking fit. From such teeth, basal cladotherians (e.g., *Amphitherium*; Fig. 2) evolved more precisely occluding teeth in which the lower molars possess a distally projecting talonid, initially consisting of only a single small distal cusp. This talonid may have initially served as an interlocking mechanism with the succeeding lower molars, but it also added another shearing facet between upper and lower teeth during mastication. Many non-tribosphenic trechnotherians (e.g., “symmetrodontans,” dryolestoids, stem zatherians) retained or slightly modified this early tooth morphology in which the talonid supported only a single cusp that is now regarded as homologous with the hypoconid of tribosphenic molars (see Davis 2011a). More advanced non-tribosphenic trechnotherians called “peramurans” evolved a talonid with a second cusp (hypoconulid). Although it still lacked a basin, the expanded talonid participated more fully in mastication with the opposing upper molar.

The final step towards the first tribosphenic molar was the acquisition of a molar talonid basin, which appears to have happened with the acquisition of the protocone (Lopatin and Averianov 2007). One of the most plesiomorphic examples of a talonid basin and protocone is found in the Early Cretaceous basal boreosphenidan *Kielantherium gobiense*. The protocone is small and the talonid bears two cusps; the hypoconid and hypoconulid.

This two-cusped talonid morphology was modified further in derived tribosphenic mammals. The appearance of the lingual talonid cusp (entoconid) of the lower molar, which helped to close the lingual side of the talonid to form a basin (Davis 2011a) may have functioned to keep food in place within the talonid basin during the final mortar and pestle phase of the chewing stroke (Crompton and Hiiemae 1970; Crompton and Kielan-Jaworowska 1978). These developments resulted in the basic pattern of molar occlusion of living therian mammals, in which lower molars have a basined talonid (crushing heel) and trigonid (shearing end). The tribosphenic molar of therian mammals is not to be confused with the superficially similar pattern found in taxa grouped as australosphenidans and shuotheriids in some phylogenies (Davis 2011b; Luo et al. 2001). Lower molars of those groups (upper molars are not known in australosphenidans) indicate that the talonid (or pseudo-talonid of pseudo-tribosphenic mammals in the case of shuotheriids, see Luo et al. 2007b) may not be completely homologous or functionally identical to the tribosphenic molars of boreosphenidans (see Davis 2011a).

Recent studies in experimental developmental biology (e.g., Harjunmaa et al. 2014) are shedding light on the evolution of the mammalian dentition by showing how genetic modifications can alter developmental pathways that control tooth shape. Although most of these studies focus on mice (*Mus musculus*), rodents with highly derived dentitions, some studies (e.g., Moustakas et al. 2011) have examined the effects of genetic modification on tooth development and patterning in the extant metatherian *Monodelphis domestica* (the short-tailed opposum), an animal that retains a relatively plesiomorphic, heterodont dentition. Studies such as these will undoubtedly lead to a better understanding of the molecular mechanisms underlying the evolution of mammalian teeth. The evolution of the distinctive tightly interlocking occlusal molar pattern of tribosphenic mammals was accompanied by necessary changes in tooth ontogeny and growth. In Mammalia, tooth replacement was suppressed further. This is likely related to the determinate growth pattern of the skull (Luo et al. 2004). Teeth are replaced no more than once (diphyodonty) so that an initial set of deciduous teeth were replaced by a second generation of permanent teeth, or teeth, such as molars, were not replaced at all.

### Theria

Theria is a clade of boreosphenidan mammals that is defined as the most recent common ancestor of extant marsupials and placentals and all of its descendants. The only extant members of boreosphenidans can be grouped into two sister clades, the Metatheria (living marsupials and their close fossil relatives) and Eutheria (living placentals and their close fossil relatives). The most recent and comprehensive molecular clock divergence estimates place the origin of Theria approximately 170–190 million years ago (e.g., dos Reis et al. 2012, 2014). The oldest confidently identified therian fossil is the eutherian *Juramaia* from the early Late Jurassic (ca. 160 million years old) of China (Luo et al. 2011). Because Metatheria and Eutheria are sister clades, the age of *Juramaia* implies that metatherians and eutherians must have diverged from each other by 160 million years ago.

## Metatheria

### Definition

We follow the stem-based definition of Kielan-Jaworowska et al. (2004), in which Metatheria is defined as all mammals more closely related to living marsupials (such as kangaroos and opossums) than to living placentals (such as humans and hedgehogs) and monotremes. According to most recent phylogenetic analyses (Luo et al. 2003; Luo et al. 2011; Rougier et al. 2004), Metatheria includes basal stem taxa (e.g., *Sinodelphys*), the Deltatheroida, and Marsupialiformes, with the latter group consisting of the Marsupialia (crown group metatherians) plus stem metatherians more closely related to Marsupialia than to Deltatheroida (Vullo et al. 2009).

### Diagnosis

Many of the anatomical features that are used to distinguish living Metatheria (marsupials) and Eutheria (placentals) are based on aspects of “soft anatomy” that do not commonly preserve in fossils and do not have reliable osteological correlates (Kielan-Jaworowska et al. 2004). Many of these features relate to differences in the reproductive system. Marsupials give birth to altricial young after a relatively brief gestation period compared to placental mammals, whose young are born in a more advanced developmental state (precocial). This is at least partly because marsupials have not evolved the ability to prevent immunological recognition and rejection of the young by the mother. Marsupials then undergo a prolonged period of lactation, compared to most placentals. In most, but not all, marsupials this occurs in a pouch.

Although the vast majority of features distinguishing metatherians and eutherians relate to soft tissues, there are limited characters of the dentition, cranium, and postcranial skeleton that diagnose Metatheria (or very proximal groups on the phylogeny). The possession of these features is usually taken as strong evidence that a fossil in question belongs to Metatheria.

### Dental formula, tooth replacement, and tooth homologies

Metatherians differ from eutherians in their dental formula and tooth replacement pattern. The metatherian pattern of tooth replacement is postulated to be intimately related to the metatherian reproductive process, which includes an extended period of lactation with nipple fixation following birth of extremely altricial young (Cifelli and Muizon 1997; 1998b; Cifelli et al. 1996b; but see van Nievelt and Smith 2005). However, the identification of tooth loci and resulting tooth homologies remains controversial, as does the identification of the plesiomorphic condition shared by the therian ancestor of metatherians and eutherians (Averianov et al. 2010; Cifelli et al. 1996b; Clemens 1979; Luckett 1993; Marshall and Muizon 1988; Owen 1868; Reig et al. 1987; Rich et al. 2002). These issues are complicated by the paucity of specimens preserving relatively complete tooth rows. Therefore, the dental formula of many taxa is based upon composites constructed from numerous specimens (see Wible et al. 2009). Even among specimens with relatively complete dentitions, there are sometimes differences of interpretation because some specimens may retain deciduous teeth along with their permanent successors.

In recent years, there has been a growing consensus that the plesiomorphic dental pattern for Theria consists of seven to eight postcanine teeth, consisting of four to five premolars and three molars. Many Cretaceous eutherian mammals retain this plesiomorphic dental formula, but the ancestral placental mammal has reduced the premolar number to four, based on a loss of the P3/p3 position (the P3/p3 is variably present in stem therians and early eutherians) (Averianov et al. 2010; Novacek 1986; O’Leary et al. 2013; Szalay 1977). The metatherian dentition differs from this in the loss of a permanent ultimate premolar. Under this dental replacement model, the deciduous ultimate premolar (DP5/dp5) is retained (Fig. 4). The DP5/dp5 has traditionally been identified as the M1/m1 due to its position and molariform morphology; the remaining molars were typically identified as M2–M4. Therefore, the metatherian premolar/molar formula is usually given as 3.4. However, based on this model, the remaining molars are correctly identified (in a development sense) as M1–3 and are therefore homologous with the eutherian M1–3. This pattern was certainly established among at least some metatherians in the Late Cretaceous (Cifelli and Muizon 1998a; Cifelli and Muizon 1998b; Cifelli et al. 1996b; Clemens 1966; Fox and Naylor 2006) and is hypothesized to have been shared by all metatherians (Averianov et al. 2010; O’Leary et al. 2013). The metatherian tooth replacement pattern is considered to be the most derived (in terms of most extreme modification from the ancestral condition) for all basal therian mammals (Luo et al. 2004) and diagnostic for Metatheria (Rougier et al. 1998).

The dentition mesial to P4 is not functionally replaced in Marsupialia. Some workers have argued that vestigial deciduous incisors and canines develop, but are resorbed before eruption of permanent teeth in some marsupials (Luckett 1993; van Nievelt and Smith 2005). The mesial premolars of marsupials are not replaced and some workers (Cifelli and Muizon 1998a; Cifelli et al. 1996b; Luckett 1993) have argued that these teeth should be considered deciduous teeth by default, but it may also be possible that the deciduous teeth were lost and only the successional teeth, homologous with the permanent teeth of eutherians, were present (van Nievelt and Smith 2005). McKenna (1975) suggested that the first premolar position was occupied by a retained deciduous tooth in both metatherians and eutherians. Averianov et al. (2010) suggested that the P1 position is lost in metatherians and the mesial two premolars represent retained P2 and P3. More recently, O’Leary et al. (2013) proposed that the most parsimonious scenario is that the 3rd premolar position was lost in the therian ancestor of both metatherians and eutherians. Accordingly, the first two premolar teeth in metatherians would be P1–P2, whereas the third premolar tooth would be homologous with P4 in non-therian mammals (Fig. 4).

Discrete synapomorphies of Metatheria. A list of metatherian synapomorphies recovered by recent higher-level phylogenetic analyses of mammals is given in Table 1. The list includes synapomorphies identified by Wible et al. (2009), with amendments and corrections from O’Leary et al. (2013). The O’Leary et al. (2013) analysis did not include any Cretaceous metatherians and so the soft-tissue characters included in their analysis (some of which can be assessed based on hard-tissue evidence) are based on two extant marsupial taxa.

Potential synapomorphies of Metatheria. Wible et al. (2009) identified a handful of other features as synapomorphies of Metatheria (Table 2). These were not recovered as explicit synapomorphies of Metatheria by O’Leary et al. (2013), but they acknowledged that these optimizations may change when additional taxa are added to their dataset. It is possible, therefore, that some or all of these features may diagnose Metatheria, or if not, then a very proximal portion of mammalian phylogeny.

**Figure 3.**
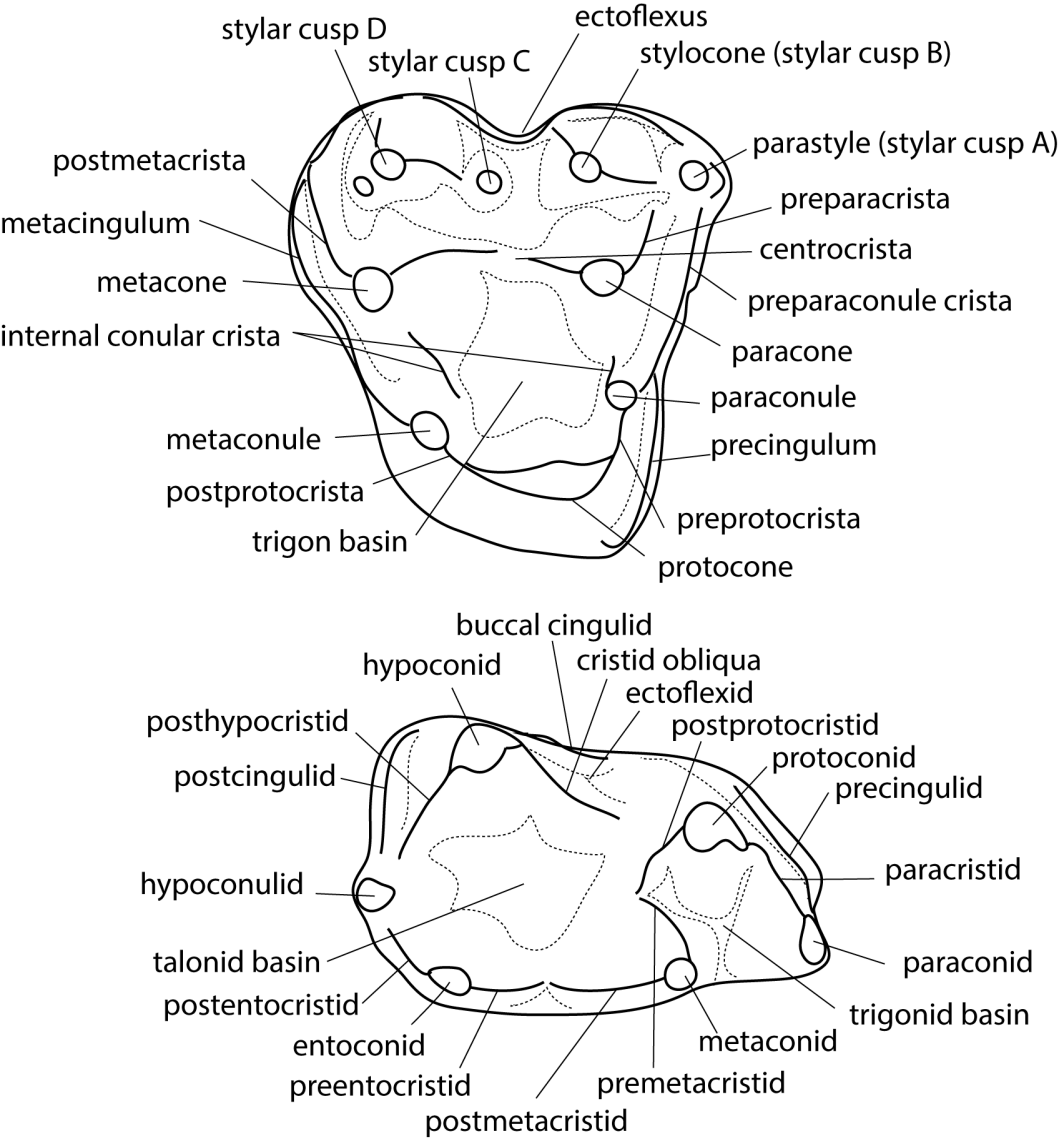
Molar tooth nomenclature of basal metatherian right M2 (upper) and left m2 (lower). Mesial is to the right, buccal is up. Teeth are based on the Late Cretaceous metatherian *Glasbius*.

**Figure 4.**
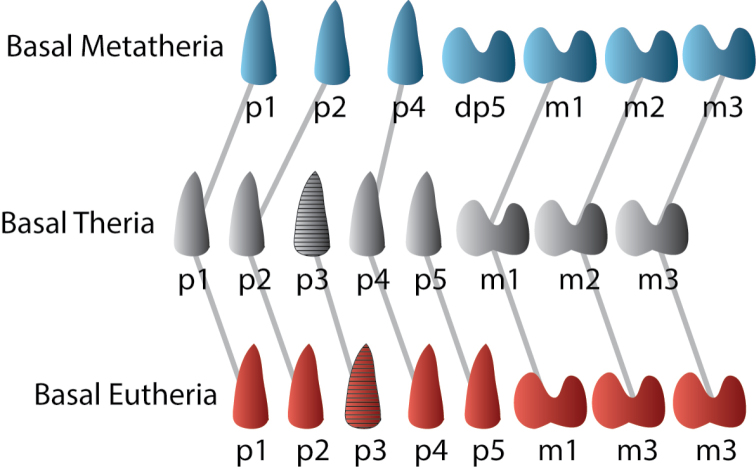
Homologies between postcanine teeth of adult basal therian, basal metatherian, and basal eutherian mammals after O’Leary et al. (2013).

**Table 1.** List of synapomorphies for Metatheria after Wible (2009) and O’Leary (2013).

**Dental:**
1) Absence of tooth replacement in the P2, p2, P5, and p5 tooth positions. The retention of the dP5 is responsible for the sharp break in the “premolar”-molar series that has been considered distinctive for Metatheria (e.g., Clemens 1979).
2) Lateral divergence of lower canines
3) dp5 cristid obliqua (=ectolophid) very trenchant
**Skull:**
1) Presence of a posterior masseteric shelf on the dentary
2) Ventral exposure of the presphenoid
3) Angular process that is equal to half but less than the length of the dentary ramus
**Postcranial:**
1) Presence of a capitular tail on the humerus
2) Testes that descend through the body wall to the scrotum
3) A small vermis in the cerebellum
4) Presence of a prehensile tail (convergently develops in many placental mammal groups including rodents, primates, carnivorans)

**Table 2.** Potential synapomorphies of Metatheria after O’Leary et al. (2013).

1) Posterior most mental foramen at ultimate premolar first molar junction or more posterior
2) Labial mandibular foramen
3) Condyloid crest absent
4) Meckelian groove absent
5) Coronoid facet absent
6) Palatal process of premaxilla reaches nearly or to canine alveolus
7) Glenoid fossa troughlike
8) Lacrimal foramen exposed on face
9) Wible et al. (2009) found the “medial course of the internal carotid artery” to be a synapomorphy of Metatheria, but O’Leary et al. (2013) found that none of the four osteological correlates that correspond to this interpretation of this feature optimizes as an unambiguous synapomorphy of Metatheria.

## A survey of Cretaceous metatherian taxa

Several major clades of Cretaceous metatherians have been recognized. *Sinodelphys* from the Early Cretaceous of China is widely considered to be a basal metatherian, sister taxon to all remaining Cretaceous–Recent forms (Luo et al. 2003; Luo et al. 2011; Vullo et al. 2009). The poorly known *Holoclemensia* from the Early Cretaceous of Texas may occupy a similar position close to the base of Metatheria (e.g., Luo et al. 2003; Luo et al. 2011; Rougier et al. 1998; Rougier et al. 2004), but alternatively it may be a basal eutherian or a stem boreosphenidan outside of Theria (e.g., Averianov et al. 2010; Kielan-Jaworowska et al. 2004; Vullo et al. 2009). All or most remaining Cretaceous metatherians fall into two principal clusters that are sister taxa to each other: Deltatheroida and Marsupialiformes (e.g., Averianov et al. 2010; Luo et al. 2003; Luo et al. 2011; Rougier et al. 1998; Rougier et al. 2004; Vullo et al. 2009; Williamson et al. 2012). Extant marsupials are members of Marsupialiformes.

### Sinodelphys

*Sinodelphys szalayi* is based on a partial skeleton compressed on a shale slab. It was originally described as an Early Cretaceous metatherian by Luo et al. (2003). This referral was based largely on the molar formula, which was interpreted to consist of four upper premolars and four upper molars and three lower premolars and four lower molars (P4M4/p3m4). The metatherian affinities of this taxon were corroborated by a phylogenetic analysis in the original paper, as well as subsequent analyses by the same authors (e.g., Luo et al. 2011). Szalay and Sargis (2006) later provided additional evidence for the metatherian position of *Sinodelphys* based on tarsal morphology, arguing that the exceptionally large and distally extending peroneal processes, and small and steeply angled calcaneocuboid articulations facing mediodistally, supported an assignment to Metatheria. However, the diagnostic utility of these characters has not been established using cladistic analysis.

The metatherian identification of *Sinodelphys* has not been confidently corroborated by subsequent phylogenetic analyses, often because it is excluded. *Sinodelphys* was not included in some recent phylogenetic analyses of metatherians because the researchers had not examined the holotype specimen (Averianov et al. 2010; O’Leary et al. 2013; Wible et al. 2009; Williamson et al. 2012). In another recent analysis based on a limited sample of dental characters, *Sinodelphys* was recovered in a basal polytomy with Metatheria and Eutheria (Vullo et al. 2009), meaning that it is equally most parsimoniously a basal metatherian or a basal eutherian. Other doubts about the metatherian affinities of *Sinodelphys* have been raised based on anatomical considerations. Averianov et al. (2010) noted that no therians have four upper and three lower premolars, raising the possibility that *Sinodelphys* may be a more basal, non-therian mammal. Averianov et al. (2010) further suggested that a diastema between the first and second preserved premolars represents a missing tooth, so that the specimen would have had one additional lower premolar, and therefore not the characteristic “three premolars” of metatherians (using the traditional terminology where the homolog of the final premolar is considered a molar, see above). In addition, they suggested that the teeth identified by Luo et al. (2003) as M1/m1 are correctly identified as P5/p5. If correct, then *Sinodelphys* would have had either five or six premolars, depending on whether the diastema between the first two preserved premolars was filled by another tooth. In either case, *Sinodelphys* would not have had a classic metatherian dental formula. *Sinodelphys* also lacks other character states often considered synapomorphic for Metatheria, such as a medially inflected angular process of the dentary. However, this character was not found to be a synapomorphy for Metatheria by O’Leary et al. (2013, see Tables 1 and 2).

We provisionally accept *Sinodelphys* as a basal metatherian, noting that it does share some features with other metatherians, and most published phylogenetic analyses recover it as a metatherian. If *Sinodelphys* is one of the earliest diverging metatherian lineages, then it may not be surprising that it lacks certain derived character states that are seen in all later metatherians (such as the inflected angular process and the characteristic metatherian tooth formula). In this scenario, some characters long considered metatherian hallmarks are actually diagnostic of a slightly less inclusive clade that does not include *Sinodelphys*, and possibly other early-diverging metatherians. Although we accept *Sinodelphys* as most likely belonging to Metatheria, this must be corroborated by additional phylogenetic analyses that include a large swath of Mesozoic mammals and a broad sampling of dental and non-dental characters.

### Deltatheroida

Deltatheroidans were long regarded as eutherians (Gregory and Simpson 1926; Van Valen 1966) or stem boreosphenidan species (Fox 1974; Kielan-Jaworowska et al. 1979), but are now generally accepted as basal metatherians (Butler and Kielan-Jaworowska 1973; Kielan-Jaworowska and Nessov 1990; Rougier et al. 1998). Deltatheroidans are best known from Asia (Averianov et al. 2010; Rougier et al. 1998) where they were the most common metatherians in the Late Cretaceous. Asian genera include *Sulestes* from the Turonian of Uzbekistan and *Deltatheridium* and *Deltatheroides* from the Campanian of Mongolia and surrounding areas. However, the oldest occurrences are the genera *Atokatheridium* and *Oklatheridium* from the Early Cretaceous of North America (Davis et al. 2008; Kielan-Jaworowska and Cifelli 2001). The group persisted, but remained rare, through the Late Cretaceous of North America, surviving into the latest Cretaceous and most likely up to the Cretaceous-Paleogene boundary (Fox 1974; Wilson 2013; Wilson and Riedel 2010).

### Marsupialiformes

The sister taxon to Deltatheroida is a large clade that includes most remaining metatherians, including crown Marsupialia. The nomenclature of this clade and its major subclades has a confusing history in the literature, with different authors using various names and various taxonomic groupings, many of which are paraphyletic or polyphyletic based on more recent phylogenetic analyses. The non-deltatheroidan Cretaceous metatherians were placed in Ameridelphia in the influential review of Kielan-Jaworowska et al. (2004), although this taxon was originally established by Szalay (1982) to contain only North and South American taxa, including those from the Cretaceous (Szalay 1994b). However, because most workers consider australidelphians (the South American microbiotheres, including the living Monito del monte [*Dromiciops gliroides*], the living Australian marsupials, and their closest fossil relatives) to have arisen from Ameridelphia (Muizon and Cifelli 2001), Ameridelphia is paraphyletic as originally conceived (Kielan-Jaworowska et al. 2004). Another group name, Alphadelphia, is also paraphyletic; it is a cohort erected by Marshall et al. (1990) and revised by Case et al. (2005) as a group of stem metatherians on the line to marsupials (crown group metatherians). Furthermore, Didelphimorphia has been used informally as an unnatural group to include only Cretaceous North American metatherians (Kielan-Jaworowska et al. 2004). It must be paraphyletic if North American metatherians gave rise to Marsupialia and extinct South American clades.

More recently, Vullo et al. (2009) erected the taxon Marsupialiformes to refer to the clade that comprises the Marsupialia (crown group metatherians) and all taxa that are more closely related to them than to the Deltatheroida and the most basal metatherians (such as *Sinodelphys*). Under this taxonomic arrangement, taxa previously grouped together as “Ameridelphia” are a paraphyletic grade of stem marsupialiforms on the line to marsupials. Given the well-established sister-group relationship between the Deltatheroida and the Marsupialiformes, and the paraphyletic nature of once-used names like the Ameridelphia, our preferred taxonomic arrangement uses the Marsupialiformes and disposes of the Ameridelphia, Alphadelphia, Didelphimorphia, and other names that are no longer tenable in phylogenetic taxonomy.

### Basal stem marsupialiforms

There is a stem grade of early-diverging marsupialiforms that do not fit easily into the major Cretaceous marsupialiform clades. Taxa that appear to populate this grade, based on recent phylogenetic studies, including *Adelodelphys*, *Sinbadelphys*, *Kokopellia*, *Arcantiodelphys*, *Anchistodelphys*, *Aenigmadelphys*, *Eoalphadon*, *Apistodon*, *Iugomortiferum*, and *Bistius*. *Bistius* was considered to possibly represent a stem therian by Clemens and Lillegraven (1986), but Williamson et al. (2012) found it to be well nested within metatherians. Similarly, Cifelli and Eaton (2013) recently suggested that *Dakotadens* and the European *Arcantiodelphys* are not metatherians, but stem boreosphenidans. Williamson et al. (2012) did not include *Arcantiodelphys* in their analysis because it was too fragmentary, but they recovered *Dakotadens* as well nested within Metatheria. Cifelli (1990b) suggested that *Iugomortiferum* might also represent a stem boreosphenidan.

Recent phylogenetic analyses of Cretaceous metatherian taxa find little resolution of the relationships of the most basal marsupialiform taxa and only weak support for many proposed clades within Marsupialiformes. The Stagodontidae may be a monophyletic group, but inclusion of *Pariadens* is only weakly supported (Williamson et al. 2012). Although “Alphadontidae” has long been used to group taxa closely related to *Alphadon marshi*, it is now considered to be either paraphyletic (Kielan-Jaworowska et al. 2004) or polyphyletic (Williamson et al. 2012). Moreover, many taxa once placed within the genus *Alphadon* have now been split off into other genera (e.g., *Protalphadon*, *Nortedelphys*, *Varalphadon*, *Turgidodon* Case et al. 2005; Cifelli 1990a; Johanson 1996b). Little phylogenetic resolution was found among these taxa in a recent comprehensive phylogenetic analysis, although some small clusters of monophyletic groups (roughly corresponding to the major “alphadontid” genera) were recovered (Williamson et al. 2012).

### Stagodontidae

Stagodontids are a small clade that includes the Campanian *Eodelphis* and the Maastrichtian *Didelphodon*, both from North America. This clade was defined by Williamson et al. (2012) as the most inclusive clade (stem-based) containing *Didelphodon vorax*, but not *Glasbius intricatus*, *Dakotadens morrowi*, *Turgidodon praesagus*, *Herpetotherium fugax*, *Peradectes elegans*, *Pediomys elegans*, or *Didelphis virginiana*.

Stagodontids are the largest metatherians of the Mesozoic and in all faunas where they are present they are the among the largest therian species. They are also commonly recognized in the fossil record by their distinctive teeth. The premolars are massive and inflated (Clemens 1966; Clemens 1968a; Clemens 1979; Fox and Naylor 2006; Lofgren 1992). The lower molars have an enlarged paraconid with a well-developed carnassial notch in the paracristid. The upper molar postmetacrista is also well developed, indicating the importance of postvallum/prevallid shear (Fox and Naylor 2006; Muizon and Lange-Badré 1997). The lower molar metaconid and upper molar preparacrista, on the other hand, are relatively small, indicating that prevallum/postvallid shear was relatively less important (Fox and Naylor 2006). However, these shearing features were obliterated through wear in older animals, which may have relied more on a “crushing and grinding” function (Fox and Naylor 1995; 2006).

Unequivocal stagodontids are known only from the middle Campanian through Maastrichtian of North America (Fox and Naylor 2006). However, Cifelli (2004) and Cifelli and Eaton 1987) tentatively referred the Albian-Cenomanian North American *Pariadens* to Stagodontidae based on its large molar size, relatively large paraconids, and a distinctive lower molar paraconid keel. The phylogenetic analysis of Williamson et al. (2012) supports this assignment, placing *Pariadens* as the earliest-diverging stagodontid, the immediate outgroup to a clade consisting of *Eodelphis* and *Didelphodon*.

### Pediomyidae

The Pediomyidae is one of the few marsupialiform clades that is strongly supported by explicit synapomorphies and high tree-support values in phylogenetic analyses (Davis 2007; Williamson et al. 2012). Williamson et al. (2012) defined this clade as the most inclusive clade (stem-based) containing *Pediomys elegans*, but not *Peradectes elegans*, *Herpetotherium fugax*, *Didelphis virginiana*, or *Didelphodon vorax*. Robust phylogenetic evidence that such a clade exists was provided by Davis (2007), who revised the taxonomy and systematics of the Pediomyidae and demonstrated that a suite of taxa historically referred to *Pediomys* (but now assigned to a variety of species of *Pediomys*, *Protolambda*, and *Leptalestes*) can be grouped together exclusive of other Cretaceous metatherians. The phylogenetic analysis of Davis (2007) found *Aquiladelphis* and *Glasbius* as more closely related to the various species of *Pediomys* than to other Cretaceous metatherians, and therefore as pediomyids following the definition of Williamson et al. (2012). The analysis of Williamson et al. (2012) found *Aquiladelphis* deeply nested within Pediomyidae but *Glasbius* as a more distantly related, non-pediomyid taxon.

Pediomyids first appear in the Santonian Milk River Formation of Alberta (Fox 1971) and reach a relatively high taxonomic and morphological diversity in Late Cretaceous faunas in northern North America. Larger-bodied taxa such as *Aquiladelphis* and *Protolambda* resemble stagodontids in emphasizing crushing and postvallum/prevallid shear.

### Glasbiidae

*Glasbius* is one of the most dentally distinctive Cretaceous metatherians (Archibald 1982; Clemens 1966, 1979; Davis 2007; Williamson and Weil 2008; Wilson 2013). Its molars are relatively small, but have low and bulbous cusps that include a large and elongate protocone with a lingually positioned metaconule. The stylar cusps are large, nearly as large as the paracone and metacone, and positioned lingual to the buccal margin of the tooth. The metacone is larger than the paracone. In addition, some of the upper molars also typically possess a mesial basal cingulum, an unusual feature among Cretaceous metatherians. The lower molars possess a broadly expanded talonid, a buccal cingulid that sometimes sports accessory cuspules, a cristid obliqua that meets the trigonid buccal to the protocristid notch, and an ultimate molar that is reduced in size relative to the other molars.

*Glasbius* has been found as a close relative or member of Pediomyidae by several phylogenetic analyses, including the analysis of Rougier et al. (1998) and its many offshoots (e.g., Averianov et al. 2010; Rougier et al. 2004; Wilson and Riedel 2010) and that of Davis (2007). Case and others allied *Glasbius* with *Hatcheritherium*, a poorly known latest Cretaceous taxon represented only by upper molars (Case et al. 2005). However, Williamson et al. (2012) did not find support for either of these relationships. Instead, their phylogenetic analysis placed *Glasbius* as the sister taxon to the South American Paleogene taxon *Roberthoffstetteria*, corroborating the close relationship between these two genera noted previously by several workers (Case et al. 2005; Goin et al. 2003; Marshall et al. 1990). If this topology is correct, it would therefore require that the *Glasbius* + *Roberthoffstetteria* clade crossed the K-Pg boundary; one of the few metatherian mammal lineages to do so (Williamson et al. 2012).

The taxon Glasbiidae was not defined by Williamson et al. (2012) and has not to our knowledge previously been defined. To provide nomenclatural stability and make it more explicit to assign taxa to Glasbiidae based on phylogenetic analyses, we here restrict Glasbiidae to the genus *Glasbius*. According to the topology of Williamson et al. (2012), *Roberthoffstetteria*, a South American taxon that is commonly considered a basal polydolopimorph, is the sister taxon to Glasbiidae. Clemens (1966) in his original description of the genus considered a wide array of possible taxonomic affinities for *Glasbius*, with particular attention to what he concluded was convergent evolution of bunodont molars within the South American Microbiotheriidae and Caroloameghiniidae. Some authors have since rejected this notion (but see Kirsch et al. 1997) and variously argued for close phylogenetic relationship of *Glasbius* to either the caroloameghiniids (e.g., Marshall et al. 1990), more broadly within (Case et al. 2005) or sister to the Polydolopimorphia (Goin et al. 2003), or within the Paucituberculata (Kielan-Jaworowska et al. 2004). Kirsch et al. (1997) joined the Polydolopimorphia and Paucituberculata (including the living caenolestids) into a new cohort Pseudiprotodontia, although they excluded *Glasbius* from this arrangement. In a recent cladistic analysis of bunodont metatherians, Goin et al. (2009) rejected the concept of Pseudiprotodontia and instead recovered a clade comprising *Microbiotherium*, *Glasbius*, and Polydolopimorphia (including *Roberthoffstetteria*) that was supported by three synapomorphies. Similarly, a consensus tree from Oliveira and Goin (2011) also showed a possible sister-taxon relationship between the Microbiotheria and the Polydolopimorphia (including *Roberthoffstetteria*). Because these cladistic analyses do not have comprehensive taxon sampling or well-supported trees, the possible phylogenetic relationships between *Glasbius* and South American taxa (polydolopimorphs, paucituberculatans, and microbiotherians) should be viewed cautiously.

### Herpetotheriidae

Herpetotheriidae was defined by Williamson et al. (2012) as the most inclusive clade (stem-based) containing *Herpetotherium fugax*, but not *Peradectes elegans*, *Pediomys elegans*, *Didelphodon vorax*, or *Didelphis virginiana*. This is one of the most species-rich North American metatherian clades of the Paleogene (Korth 2008), and they are represented by nearly complete skulls and skeletons (Horovitz et al. 2008; Horovitz et al. 2009; Sánchez-Villagra et al. 2007). Recent phylogenetic analyses that include relatively complete cranial material of Paleogene peradectid (*Mimoperadectes*) and herpeotheriid taxa indicate that herpetotheriids are the sister taxon to crown clade Marsupialia (Horovitz et al. 2009).

Unequivocal herpetotheriids are relatively common in the fossil record of North America after the Cretaceous-Paleogene boundary. There is some evidence, however, for more basal herpetotheriids in the Cretaceous. Case et al. (2005) argued that the poorly known Campanian taxon *Ectocentrocristus* represents a herpetotheriid, along with another genus, *Nortedelphys*, that includes three species from the Maastrichtian of North America. *Ectocentrocristus* remains problematic because it is based on what may be a deciduous P4, possibly of a *Turgidodon*-like taxon (Beck et al. 2008; Cifelli in Kielan-Jaworowska et al. 2004; see Williamson et al. 2012). The phylogenetic analysis by Williamson et al. (2012) and here (below) supports the placement as a basal herpetotheriid of *Ectocentrocristus*, but not *Nortedelphys*, which is found to be a more basal marsupialiform. This provides additional evidence for a Late Cretaceous origin of Herpetotheriidae, suggesting that this clade crossed the K-Pg boundary.

### Peradectidae

This clade was defined by Williamson et al. (2012) as the most inclusive clade (stem-based) containing *Peradectes elegans*, but not *Herpetotherium fugax*, *Pediomys elegans*, or *Didelphis virginiana*. This clade comprises almost entirely post-Cretaceous taxa, and was recently hypothesized to be the sister taxon of living opossums (Didelphidae) and other members of crown clade Marsupialia based on a comprehensive higher-level phylogenetic analysis of fossil and living marsupials (Horovitz et al. 2009). Historically, workers have assigned various Cretaceous species to Peradectidae, including some species of *Alphadon* (Crochet 1979; 1980). These species are either not clearly referable to Peradectidae based on phylogenetic analyses (e.g., Williamson et al. 2012) or are now considered as Paleocene-Eocene in age (Sigé et al. 2004).

The phylogenetic analysis of Williamson et al. (2012) recovered a massive polytomy comprising many species traditionally referred to Peradectidae as well as many non-peradectid metatherians. Because of this, when the phylogenetic definition of the clade is strictly applied according to the Williamson et al. (2012) topology, only two taxa fall into a restricted Peradectidae (the name-bearing definitional taxon *Peradectes elegans* and its sister taxon *Peradectes californicus*). Williamson et al. (2012) discussed how this result was likely due to missing data in several taxa, and anticipated that future phylogenetic analyses would successfully group together all or most traditionally regarded peradectids into a clade. This clade may or may not include the Cretaceous taxon *Maastrichtidelphys*.

### A note on metatherian taxonomy

**Figure 5.**
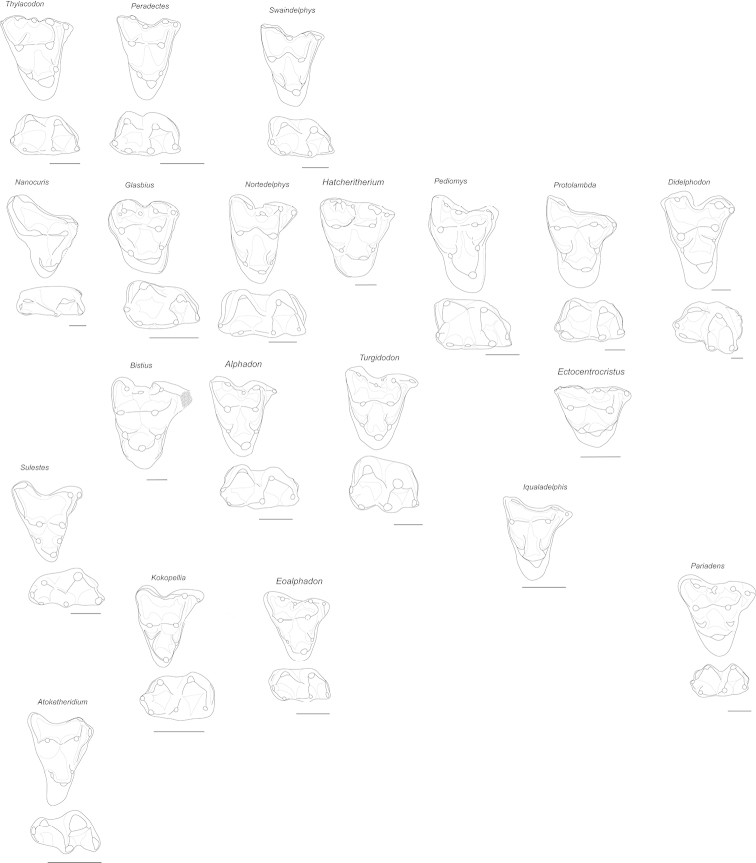
Representative upper and lower molars of Cretaceous and early Paleogene metatherians. Scale bars equal 1 mm.

As for the vast majority of Cretaceous mammals, Cretaceous metatherians are usually represented by isolated teeth. Therefore, most phylogenetic analyses and taxonomic arrangements rely heavily on dental characters, especially size and relative size, shape, and position of various tooth features (Fig. 5). Identification is complicated by the change in tooth shape according to position within the tooth row, but teeth typically change in a predictable fashion. Further, few upper dentitions are found in direct association with lower dentitions. Fortunately, because molars of Mesozoic therian mammals have precise occlusion, the shape of upper molars can be used to predict the shape of the occluding lower molars and vice versa. Moreover, during mastication, upper and lower molars contact each other, resulting in distinctive wear facets. These facets can provide clues to the shape of the opposing teeth and factors regarding the mandibular movement and occlusion (Crompton and Hiiemae 1970; Crompton and Kielan-Jaworowska 1978). Teeth of metatherians form an intricate functional complex that is intimately related to diet (Gordon 2003b) and tooth size is highly correlated with mass of the animal (Gordon 2003a).

Although teeth can usually be used to confidently distinguish metatherians from other mammals and give some insight into body size and diet, there are difficulties associated with using teeth for higher-level metatherian taxonomy and phylogeny. Some of the differences used to distinguish teeth of various taxa are subtle. In addition, some taxa are represented only by small samples, in some cases only a few fragmentary teeth. Therefore, the range of morphological variation within some taxa is poorly understood. This, no doubt, partly explains why there is considerable disagreement and skepticism surrounding some identifications of some taxa and the validity and taxonomic decisions regarding synonymies of other taxa (see Eaton 2013; Eaton and Cifelli 2013; Williamson et al. 2012 for recent discussions regarding the taxonomy of metatherian taxa). This also helps explain rampant homoplasy and polytomies in some phylogenetic analyses, as well as why competing analyses with different taxon- and character-sampling regimes produce different results.

### Phylogenetic analysis

Metatherians underwent a substantial radiation during the Late Cretaceous, and while several Cretaceous metatherians are known from the Late Cretaceous of Europe and Asia (e.g., Averianov et al. 2010; Martin et al. 2005; Rougier et al. 2004; Szalay and Trofimov 1996; Vullo et al. 2009), the majority are known from North America where they are represented almost exclusively by jaws and teeth.

The interrelationships of most major metatherian subclades are unresolved. Several recent cladistic analyses have looked at the higher-level phylogenetic relationships of mammals and have included some Cretaceous and Paleogene metatherian taxa (Ji et al. 2002; Kielan-Jaworowska et al. 2004; Luo et al. 2003; Luo et al. 2002). Other analyses have focused on the relationships within Metatheria and the position of Metatheria within Mammalia (Horovitz et al. 2008; Horovitz and Sánchez-Villagra 2003; Rougier et al. 2004; Sánchez-Villagra et al. 2007), and others have explored the relationships of a small number of Cretaceous taxa within Metatheria (Davis 2007; Johanson 1996a; Martin et al. 2005; Vullo et al. 2009).

Williamson et al. (2012) provided the first analysis to include nearly complete, species-level coverage of Cretaceous and early Paleogene metatherian taxa. The resulting phylogeny was used to examine the pattern of metatherian survivorship across the K-Pg boundary and the origin and evolution of early Paleogene North American metatherian taxa. The dataset consists only of dental characters, because the majority of Cretaceous-Paleocene metatherians are represented solely by teeth. We here present a revised analysis of Mesozoic and early Paleogene metatherian taxa built upon that of Williamson et al. (2012).

The revised analysis presented here includes 95 taxa and 83 characters. It adds three non-metatherian taxa to those included in the Williamson et al. (2012) analysis: *Juramaia sinensis*, *Asioryctes nemegetensis*, and *Ukhaatherium nessovi*. The analysis thus includes four basal eutherians, six deltatheroidans, 59 Cretaceous marsupialiaforms, and 26 Paleogene marsupialiforms. *Juramaia* is used as the outgroup taxon. We have modified the character descriptions to reflect the postcanine dental homologies as proposed by O’Leary et al. (2013). Based on this, the first tooth in the molar series in Metatheria is homologous to the deciduous p5 (dp5) of eutherians (O’Leary et al. 2013) (Fig. 4).

We added five new characters (characters 7, 15, 51, 52, and 68; see Suppl. material 1), slightly changed character 85 (now character 83), and deleted one character (Williamson et al. 2012, character 15). As with the previous Williamson et al. (2012) analysis, we excluded taxa that are represented only by lower dentitions that are very incomplete (e.g., *Esteslestes*, *Arcantiodelphys*), or that we believe are invalid (see Williamson et al. 2012). We have also excluded *Pappotherium* and *Holoclemensia*, poorly known taxa that may be outside of Metatheria (see Williamson et al. 2012) and that acted like wildcard taxa when included in a preliminary version of our analysis.

Minor changes or corrections were made to the scoring of some taxa from the Williamson et al. (2012) dataset; a total of 10 changes are listed in Suppl. material 1.

We conducted a parsimony analysis using TNT v. 1.1, September 2013 (Goloboff et al. 2008). We first analyzed the matrix (Suppl. material 2) under the “New Technology Search” with Sectorial Search, Rachet, Drift, and Tree Fusing, finding the minimum length tree 10 times. The 21 recovered trees were then analyzed under traditional tree bisection reconnection (TBR) swapping, to more extensively explore each tree island. This resulted in 2,008 most parsimonius trees of length 500 (consistency index = 0.210; retention index = 0.703).

## Results of phylogenetic analysis

**Figure 6.**
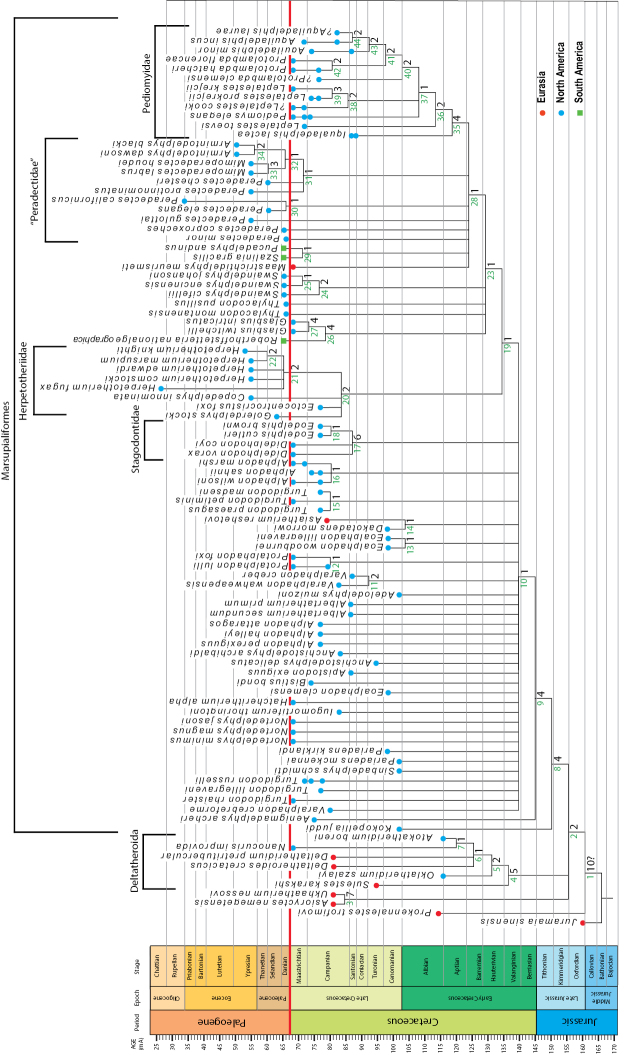
Strict consensus of 2008 trees of 500 steps calculated using TNT (Goloboff et al. 2008) based on a taxon-character matrix of 95 taxa and 83 dental characters (CI = 0.210; RI = 0.703). Analysis conducted using a New Technology Search with a Driven Search (Sectorial Search, Ratchet, Drift, and Tree Fusing), finding minimum length 10 times. All characters are unweighted and 19 characters are additive. Numbers to the left of each node correspond to nodes in Online Suppl. material 1 listing the synapomorphies common to the 500 shortest trees. Numbers to the right of each node correspond to Bremer branch supports calculated from a pool of 30,000 suboptimal trees of up to 10 steps longer than the shortest trees obtained.

Parts of our strict consensus tree (Fig. 6) are well resolved, but individual support values for many clades are low (see Fig. 6 for Bremer support values). As in the previous analysis of Williamson et al. (2012), homoplasy is rampant. These issues are most likely due to the large amounts of missing data in some taxa, as well as the nearly 1:1 ratio of taxa and characters, an unavoidable problem that can be remedied in future analyses with the addition of non-dental characters (although at the present time very few taxa could be scored for any non-dental characters). A list of synapomorphies for each node is provided in Suppl. material 1.

The topology of the strict consensus tree is similar to that recovered by Williamson et al. (2012). Salient differences are related to the inclusion of two additional basal eutherian taxa, *Asioryctes* and *Ukhaatherium*, which form a clade that is placed in a polytomy with Deltatheroida and Marsupialiaformes. In the revised analysis, the Cenomanian *Pariadens* is not recovered as a basal stagodontid, lending support to the conclusion reached by Fox and Naylor (2006) that neither species of *Pariadens* is a stagodontid.

As in the previous analysis by Williamson et al. (2012), *Thylacodon* and *Swaindelphys*, a clade containing *Glasbius* and *Roberthoffstetteria*, Pediomyidae, and an assemblage composed of “Peradectidae sensu lato” (all species of *Peradectes*, *Armintodelphys*, *Mimoperadectes*, *Maastrichtidelphys*, *Szalinia*, and *Pucadelphys*) are in a polytomy. *Golerdelphys* (= “Goler Formation taxon” of Williamson et al. 2012), *Ectocentrocristus*, and *Copedelphys* are in a polytomy with a clade composed of species of *Herpetotherium*.

Species of *Nortedelphys* do not fall within Herpetotheriidae or any other Paleogene metatherian taxon, contra Case et al. (2005). The latest Cretaceous European *Maastrichtidelphys* was also proposed to be a herpetotheriid based on a phylogenetic analysis that did not include undisputed herpetotheriid taxa (Martin et al. 2005). Our analysis does not support a close relationship between *Maastrichtidelphys* and Herpetotheriidae. Rather, the late Campanian taxon *Ectocentrocristus* shows a close, but unresolved relationship with *Golerdelphys* and taxa traditionally assigned to Herpetotheriidae (e.g., *Copedelphys*, *Herpetotherium*). Our analysis also does not support a close relationship between species of *Alphadon* and the earliest Paleocene species of *Thylacodon*, *Peradectes*, or any Paleogene metatherian taxa.

A strong link exists between the enigmatic latest Cretaceous *Glasbius* and the early Paleocene *Roberthoffstetteria*, in agreement with previous suggestions (Case et al. 2005; Goin et al. 2003; Marshall and Muizon 1988; Muizon 1992).

## Osteology of Metatheria

The crania and postcrania of Cretaceous metatherians are poorly known. Most taxa are represented only by teeth and jaw fragments. Only two specimens that include articulated skulls and partial skeletons have been described: the stem marsupialiform *Asiatherium* from the Campanian of Mongolia (Szalay and Trofimov 1996; Trofimov and Szalay 1994; Fig. 6) and the early-diverging metatherian *Sinodelphys* from the Early Cretaceous (Barremian) of China (Luo et al. 2003).

The specimens of *Asiatherium* and *Sinodelphys* include nearly complete skulls that provide some of the only information on Cretaceous metatherian cranial anatomy. A nearly complete skull from Gulin Tsav (or Guriliin Tsav), Mongolia, the “Guriliin Tsav Skull” (Kielan-Jaworowska and Nessov 1990; Szalay and Trofimov 1996), remains undescribed, but has been included in several phylogenetic analyses (e.g., Averianov et al. 2010; Rougier et al. 1998). It was provisionally assigned to Deltatheroida by Szalay and Trofimov, but other workers (e.g., Averianov et al. 2010; Rougier et al. 1998) have found it to be near the base of Marsupialiformes and possibly related to stagodontids. Partial skulls are known for several Late Cretaceous metatherian taxa, including the Asian deltatheroidans *Deltatheridium* and *Sulestes* (Averianov et al. 2010; Rougier et al. 1998). Although metatherians were more species rich and evidently numerically abundant in the Late Cretaceous of North America relative to Asia (at least based on the known fossil record), the North American specimens are more fragmentary. Fragments of the cranium have been described for the Late Cretaceous stagodontids *Eodelphis browni* (Matthew 1916) and *Didelphodon vorax* (Clemens 1966; Wilson et al. 2012a). A second ear region (AMNH 58852) referred to *Didelphodon vorax* by Clemens (1966) is a multituberculate (Wible 1990). In addition, several isolated petrosal bones, a part of the ear region, have been tentatively referred to metatherian taxa (Wible 1990; Wible and Hopson 1994) including “*Pediomys*” (*Protolambda*, following Davis [2007]) and “*Alphadon*” (*Turgidodon* following Cifelli [1990b]).

### Skull

The skulls of *Asiatherium* and *Sinodelphys* are relatively crushed, making reconstruction problematic. Extant didelphid marsupials and some early Paleocene taxa retain many plesiomorphic therian features and their skulls closely resemble the limited fossil cranial material of Cretaceous metatherians in many respects, meaning that they can be used as a guide for reconstructing Cretaceous metatherian skull morphology (Wible 1990, 2003; Argot 2001) (Fig. 8). In particular, *Pucadelphys* and *Mayulestes* from the early Paleocene of Bolivia are useful models for approximating the cranial anatomy of Cretaceous taxa, although they are probably more closely related to extant Marsupialia than to any Cretaceous taxon (Horovitz et al. 2009; Luo et al. 2003; O’Leary et al. 2013). Their skulls have both been described in detail (*Pucadelphys*: [Macrini et al. 2007; Marshall and Muizon]; *Mayulestes* [Muizon 1998]), as has the skull of the extant didelphid *Monodelphis brevicaudata* (the red-legged short-tailed opossum) (Wible 2003). These are all similar in size to many Cretaceous taxa, including *Asiatherium*, which had a skull of about 3 cm in length, about the same as that of the extant didelphid *Monodelphis domestica* (Szalay and Trofimov 1996).

The skulls of Cretaceous metatherians have a pronounced postorbital constriction of the cranium when seen in dorsal view, a braincase with well-developed sagittal and nuchal crests and high and robust zygomatic arches. However, these sagittal and nuchal crests were probably not as highly developed as they are in the living didelphid *Didelphis*, which is similar in size to *Didelphodon*, the largest Cretaceous metatherian (Gordon 2003a). Asian deltatheroidans as well as *Didelphodon* had a relatively short rostrum compared to extant didelphids.

The mammalian palate has incisive foramina situated within the premaxilla, and these are particularly large in metatherians compared to basal eutherians, extending posteriorly to the premaxillary-maxillary suture. In addition, many basal metatherians have two pairs of large palatine vacuities. The larger of these is anterior to the anterolateral part of the maxillary-palatine suture, and there is a smaller pair on the maxillary-palatine suture, at the posterolateral margins of the palatal exposure of the palatine. However, these are variably present in basal metatherians, living marsupials, and basal eutherians, so it is not clear if these are a synapomorphy of Metatheria or just a feature seen in many (but not all) species. The pterygoids of metatherians are not fused to the alisphenoids as they are in eutherians. Metatherians have a relatively larger alisphenoid than do eutherians. In some taxa, the alisphenoid contributes to the glenoid fossa and in others it extends posteriorly to form a tympanic process that partially conceals the petrosal as an alisphenoid bulla. An alisphenoid bulla is present in the undescribed Guriliin Tsav skull, *Asiatherium*, *Didelphodon*, and many extant marsupials, suggesting that it may be a feature that evolved at or near the base of Metatheria, potentially as a synapomorphy uniting the clade. However, it is lacking in *Pucadelphys* and *Mayulestes*, possibly indicating that it has evolved multiple times within Marsupialia (Muizon 1994).

Ear region. The incorporation of postdentary bones into the basicranial region of the braincase is one of the most extraordinary examples of morphological evolution in the fossil record (see Luo 2007). This transition independently occurred multiple times, including in a recent common ancestor of metatherians and eutherians. This means that metatherians have a single bone in the lower jaw (the dentary) and that the numerous postdentary bones of more basal mammaliaforms have been modified and many incorporated into the ear region. The bones of this region of the mammalian skull are morphologically complex and contain evidence of the cranial neurovasculature. Consequently, the ear region has been an important area of focus for assessing both the interrelationships of metatherians and the position of Metatheria within higher-level mammalian phylogeny.

Extant placental and marsupial mammals possess very different basicranial vasculature (Wible 1990). Stem therians possessed two major vessels running through the middle ear: a lateral head vein and a stapedial artery, which is a branch of the medial carotid artery. Eutherians possess a basicranial vasculature in which the prootic sinus and the lateral head vein completely disappear by adulthood. A new vein, the capsuloparietal emissary vein, leaves the cranial cavity through the capsuloparietal foramen and exits the skull through the postglenoid foramen (Wible 1990). In extant marsupials, and evidently also in some Cretaceous metatherians (Wible 1990), the posterior or tympanic portion of the lateral head vein becomes reduced during ontogeny and in most extant taxa, is totally lacking in the adult (Wible 1990, 2003; Wible et al. 2009). A new vessel, the sphenoparietal emissary vein, forms in marsupials as a new outlet for the prootic sinus.

Fossils may help us to better understand the evolution of this basicranial vasculature in metatherians and other early mammals. The mammalian basicranial region, especially the petrosal bone, can preserve osteological correlates associated with each vessel of the basicranial vasculature, meaning that their presence or absence can be assessed in well-preserved fossils.

Wible described several isolated petrosals from the Bug Creek Anthills of Montana, some of which he assigned to Cretaceous metatherians (Wible 1990). He found that the most complete Cretaceous petrosal, which he referred to as ‘Type A’, lacks the vascular sulci for the stapedial artery and contains a short prootic canal. In fact, the vascular grooves and canals of the Type A petrosal (Fig. 9) are essentially the same as in some recent didelphids including *Didelphis*, and he therefore concluded that it possessed the same fundamental basicranial vasculature pattern. This same pattern has also been found in the deltatheroidan metatherians *Deltatheridium* (Rougier et al. 1998) and *Sulestes* (Averianov et al. 2010). It appears, therefore, that this pattern (the carotid artery takes a medial course, the stapedial artery is absent) may characterize Metatheria. Indeed, Rougier et al. (1998), Kielan-Jaworowski et al. (2004), Wible et al. (2009) and other authors have considered this pattern to be a diagnostic character of Metatheria. Based on phylogenetic character optimization, however, O’Leary et al. (2013), concluded that this pattern is actually an ambiguous synapomorphy for Theria that is later reversed in Eutheria. Therefore, it would be plesiomorphic for Metatheria, not apomorphic. Because the O’Leary et al. (2013) analysis had a limited sampling of metatherian taxa and did not include the stem cladotherian *Vincelestes* or any Cretaceous metatherians, this optimization is subject to change with additional fossil evidence.

Mandible. The dentary of most Cretaceous metatherians has a shallow, horizontally oriented, and unfused mandibular symphysis. The canines diverge laterally, which is a synapomorphy of metatherians that was identified in the analysis by O’Leary et al. (2013; character 1411). An inflected angular process on the medial surface of the posterior mandible, which is so large that it forms a shelf, is often regarded as a synapomorphy for Metatheria, but it is absent from *Sinodelphys* which instead has a rod-like angular process that projects posteriorly (Luo et al. 2003) as in eutherian taxa. Therefore, the inflected angular process is a synapomorphy of a more exclusive group within Metatheria (e.g., Marsupialiformes + Deltatheroida), and as such its presence in a fossil is still a clear indication that the fossil is a metatherian.

Metatherians typically have several mental foramina on the mandible, and the posterior-most one is located below a position between p4 and dp5 (following the tooth terminology of O’Leary et al. 2013) or more posteriorly (this feature could not be corroborated as a synapomorphy of Metatheria, but was found to be an ambiguous synapomorphy of Theria by O’Leary et al. [2013]). Lack of a mandibular foramen was listed as a synapomorphy for Metatheria in some phylogenies (Rougier et al. 1998; Wible et al. 2009). However, Cifelli and de Muizon (1997) identified a small lateral mandibular foramen near the base of the masseteric fossa in the middle Cretaceous metatherian *Kokopellia*, and suggested that one might also be present in a specimen of *Alphadon*. However, no lateral mandibular foramen has been found in other metatherian specimens, including deltatheroidans (Averianov et al. 2010; Wilson and Riedel 2010). O’Leary et al. (2013) were also unable to corroborate this as a synapomorphy, but optimizations of this character might change with the incorporation of additional taxa in their matrix. *Kokopellia* may also preserve a remnant of a Meckelian groove on its lingual face (Cifelli and Muizon 1997), but this feature has not been detected in other metatherian specimens.

### Postcrania

**Figure 7.**
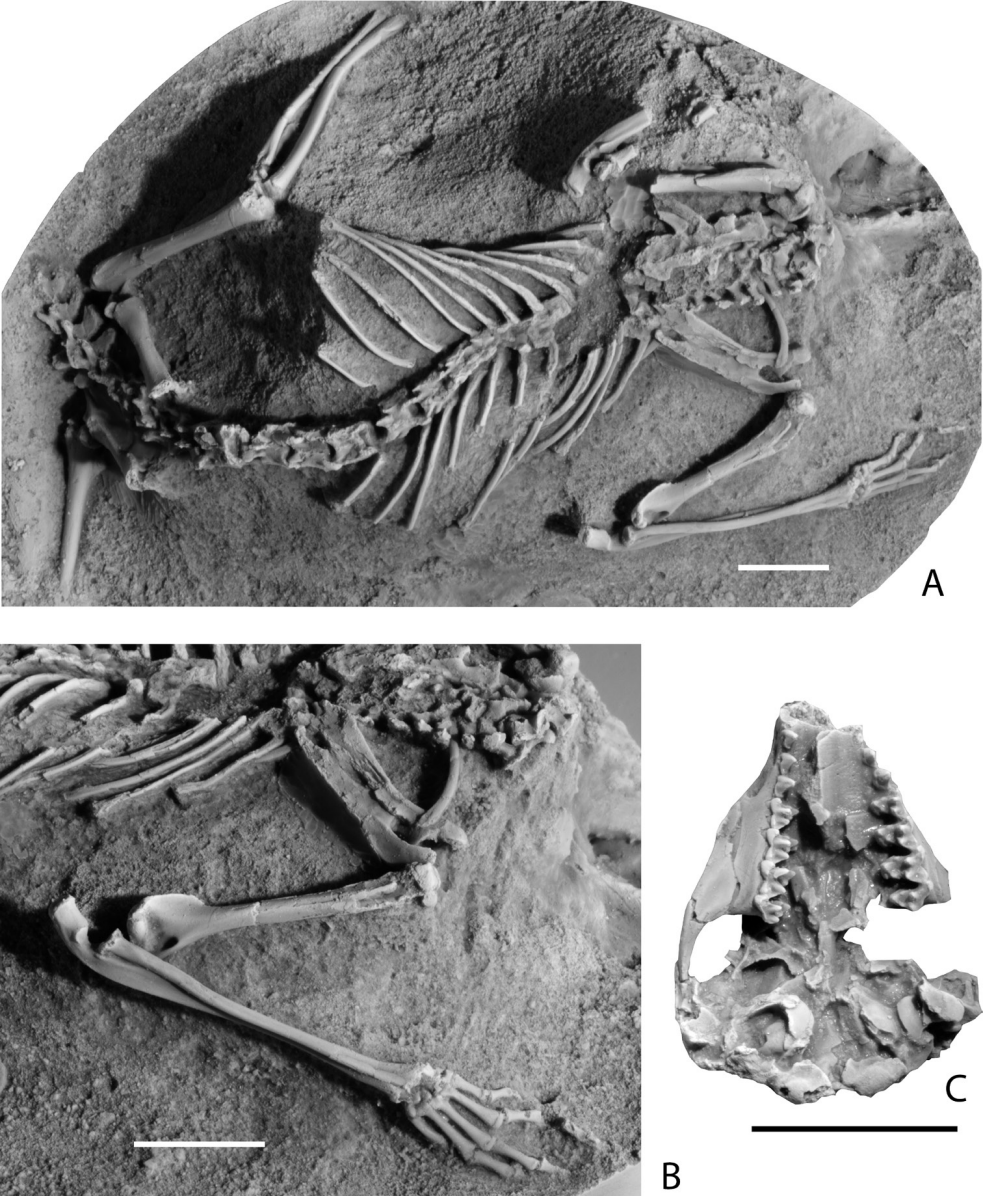
Cast of holotype of *Asiatherium reshetovi*. **A** postcranial skeleton **B** close-up of right forelimb **C** ventral view of skull. Scale bars equal 1 cm.

Associated partial postcranial skeletons (Fig. 7) are known for only two Cretaceous metatherian taxa: *Asiatherium* (Szalay and Trofimov 1996; Trofimov and Szalay 1994) and *Sinodelphys* (Luo et al. 2003). Szalay (1994a; b) referred isolated tarsal bones from the latest Cretaceous of western North America to “*Pediomys*” (probably *Protolambda*, following Davis [2007] and *Didelphodon*). These bones were collected from the Bug Creek Anthills, now regarded as a mixed assemblage of reworked latest Cretaceous and earliest Paleocene fossils (Lofgren 1995). We assume that Szalay (1994a, b) referred the isolated tarsals to “*Pediomys*” based on their large size and presumed Cretaceous age. They are too large to belong to *Thylacodon montanensis*, the only earliest Paleocene metatherian reported from eastern Montana (e.g., Wilson 2013; 2014). Longrich (2004) described isolated caudal vertebrae from latest Cretaceous North America as *Didelphodon*, but the referral of these and other isolated bones to Metatheria has been questioned by Fox and Naylor (2006). Horovitz illustrated a partial calcaneum of *Deltatheridium* from the Late Cretaceous of Mongolia (Horovitz 2000). More recently, Szalay and Sargis (2006) have described isolated tarsal elements including several calcanea and one partial astragalus, from the Bissekty local fauna of the middle Cretaceous of Uzbekistan. Other isolated elements described from the Bissekty Formation are an isolated partial humerus (Chester et al. 2010) and two partial femora (Chester et al. 2012). It is unclear how many taxa these various postcranial bones from the Bissekty Formation represent; they may belong to as many as four species, or alternatively may all belong to one taxon, *Sulestes karakshi* (Averianov et al. 2010; Chester et al. 2010; Chester et al. 2012; Szalay and Sargis 2006).

Axial skeleton. Little is known of the Cretaceous metatherian axial skeleton as it is only incompletely preserved in two specimens, the holotypes of *Asiatherium* and *Sinodelphys*. *Asiatherium* was found to lack an anticlinal vertebra and has two fused sacral vertebrae (Szalay and Trofimov 1996).

Scapula. Damaged scapulae are preserved with the partial skeletons of *Asiatherium* and *Sinodelphys*. The scapula of *Asiatherium* (Fig. 7) possesses a long and prominent scapular spine and a prominent acromion process (Szalay and Trofimov 1996). The scapula of *Sinodelphys* has a much wider supraspinous fossa than infraspinous fossa at midlength, with a strongly sigmoidal cranial profile (Luo et al. 2003); a feature found in Paleogene metatherians, but absent in Cretaceous eutherians (Luo et al. 2003). The scapula of *Asiatherium* differs in having a wider infraspinous fossa than supraspinous fossa at midlength and a nearly straight cranial margin.

Clavicle. A robust clavicle is preserved in both *Asiatherium* and *Sinodelphys*.

Humerus. *Asiatherium* and *Sinodelphys* both preserve humeri. In *Asiatherium* (Fig. 7) the humerus has a distinct and well-developed greater tuberosity and a large deltoid crest that extends about a third of the length of the bone. The distal end of the humerus has a relatively large and rounded capitulum and a large and well-developed supinator crest and capitular tail. The humerus of *Sinodelphys* has a similarly large supinator crest (Luo et al. 2003).

The three distal humerus fragments from the Bissekty Formation (Fig. 11) of Uzbekistan possess a spherical capitulum, a trochlea separated from the capitulum, and a well-developed lateral epicondylar crest. One fragment that might represent a third metatherian taxon possesses a wide capitular tail (Chester et al. 2010). The presence of a capitular tail is recognized as a synapomorphy for Metatheria by O’Leary et al. (2013; ch. 3072).

Radius and ulna. The head of the radius of *Asiatherium* (Fig. 7) is oval rather than round as in most didelphids (Szalay and Trofimov 1996; Trofimov and Szalay 1994). The anterior edge of the olecranon process of the ulna twists medially as in terrestrial didelphids and caenolestids (Trofimov and Szalay 1994).

Carpus. The carpals of both *Sinodelphys* and *Asiatherium* (Figs 7, 11) have a large cuneiform, a large unciform, and scaphoid and a small, bean-shaped trapezium, unlike non-metatherian therians and eutherians (Luo et al. 2003). In living didelphids, the enlarged hamate, triquetrum, and scaphoid in the carpus are thought to be related to increased capacity for gripping (Luo et al. 2003).

Pelvis. *Asiatherium* possesses rod-shaped ilia and epipubic bones (Fig. 7). Although often erroneously thought to be a feature of marsupials that evolved in order to support the pouch, epipubic bones are plesiomorphic for Theria and are found in basal eutherians, as well as non-therian mammals such as monotremes and multituberculates (Novacek et al. 1997; Reilly et al. 2009). Although they do support the pouch in living marsupials, epipubic bones may also relate to abdominal muscle function (Reilly et al. 2009).

Femur. The femur of *Asiatherium* (Fig. 7) lacks a distinct patellar groove, indicating that it lacked a patella, a common condition in extant marsupials (see Szalay and Sargis 2001). It lacks a third trochanter as in all extant marsupials except caenolestids and peramelids (Szalay and Sargis 2001). The lateral condyle of the femur is larger than the medial condyle. Chester et al. (2012) described two isolated metatherian femora from the Bissetky Formation (Fig. 12). These also lack a distinct patellar groove and a third trochanter. The lack of a patellar groove (and patella) may indicate arboreal ancestry (Argot 2004; Chester et al. 2012; Szalay and Sargis 2001).

Fibula. The fibula of *Asiatherium* (Fig. 7) contacts the distal femur as is typical of living marsupials, but it is not proximally expanded as in extant didelphids.

Tarsus. Associated tarsals of a Cretaceous metatherian are known only for *Sinodelphys*. (Fig. 13G, J–K). Isolated tarsals from the Late Cretaceous sediments and referred to “*Pediomys*” (likely *Protolambda* sp. following the revision of pediomyids of Davis [2007]) were described by Szalay (1994a)(Fig. 13L–M). Isolated tarsal bones tentatively referred to the deltatheroidan *Sulestes* (Fig. 13A–C, D–F) were described from the Bissetky Formation of Uzbekistan by Szalay and Sargis (2006).

The astragalus of Cretaceous metatherians is distinct from other mammals because the neck is asymmetrical, with the navicular facet of the astragalus extending medially along the length of the neck (Fig. 13).

The calcaneus of Cretaceous metatherians (Fig. 13) possesses a calcaneocuboid facet that is oblique (approximately 60 degrees) to the long axis of the calcaneus, a calcaneoastragalar facet that is nearly vertical, and a sustentacular facet that is medially facing (Luo et al. 2003; Szalay 1994a; Szalay and Sargis 2006) and adjacent to a large distally positioned plantar tubercle. This may be related to the habitual inversion of the distal part of the pes (Luo et al. 2003). The sustentacular process forms a pointed triangle.

The navicular of *Sinodelphys* (Fig. 13G) is transversely wide and short and the cuboid facet faces mediodistally.

**Figure 8.**
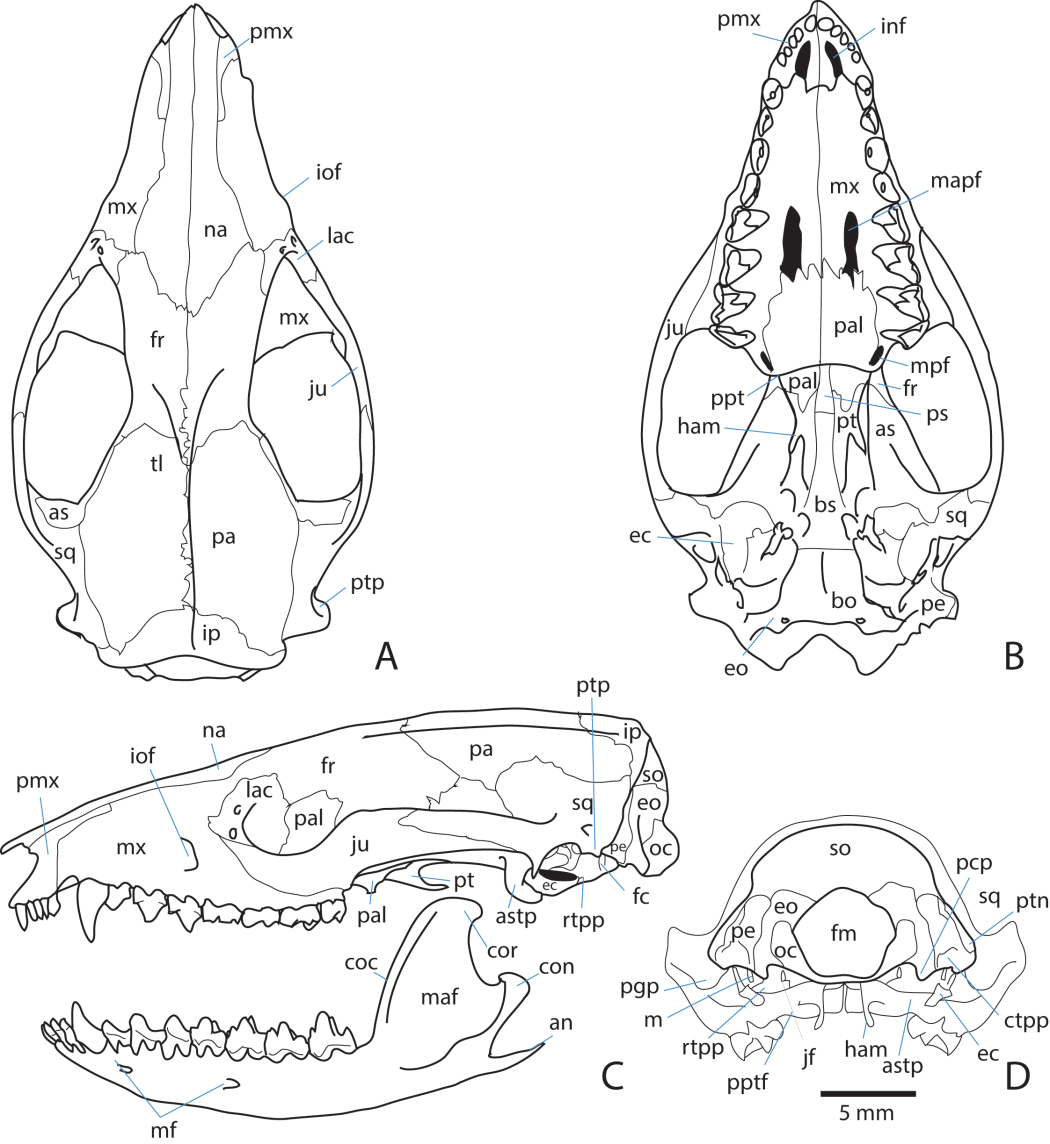
Skull of *Monodelphis brevicaudata*, modified from Wible (2003). Skull (**A–D**) and mandible (**C**) in dorsal (**A**), ventral (**B**), left lateral (**C**), and caudal (**D**) views. Abbreviations: **an** angular process; **as** alisphenoid; **astp** alisphenoid tympanic process; **bo** basioccipital; **bs** basisphenoid; **coc** coronoid crest; **con** mandibular condyle; **cor** coronoid process; **ctpp** caudal tympanic process of the petrosal; **ec** ectotympanic; **eo** exoccipital; **fc** fenestra cochleae; **fm** foramen magnum; **fr** frontal; **ham** pterygoid hamulus; **inf** incisive foramen; **iof** infraorbital foramen; **ip** interparietal; **jf** jugular foramen; **ju** jugal; **lac** lacrimal; **lacf** lacrimal foramen; **m** malleus; **maf** masseteric fossa; **mapf** major palatine foramen; **mf** mental foramen; **mpf** minor palatine foramen; **mx** maxilla; **na** nasal; **oc** occipital condyle; **pa** parietal; **pal** palatine; **pcp** paracondylar process of the exoccipital; **pe** petrosal; **pmx** premaxilla; **pgp** postglenoid process; **ppt** postpalatine torus; **pptf** foramen in the postpalatine torus; **ps** presphenoid; **pt** pterygoid; **ptn** posttemporal foramen; **ptp** posttympanic process; **rtpp** rostral tympanic process of the petrosal; **so** supraoccipital; **sq** squamosal; **tl** temporal line.

**Figure 9.**
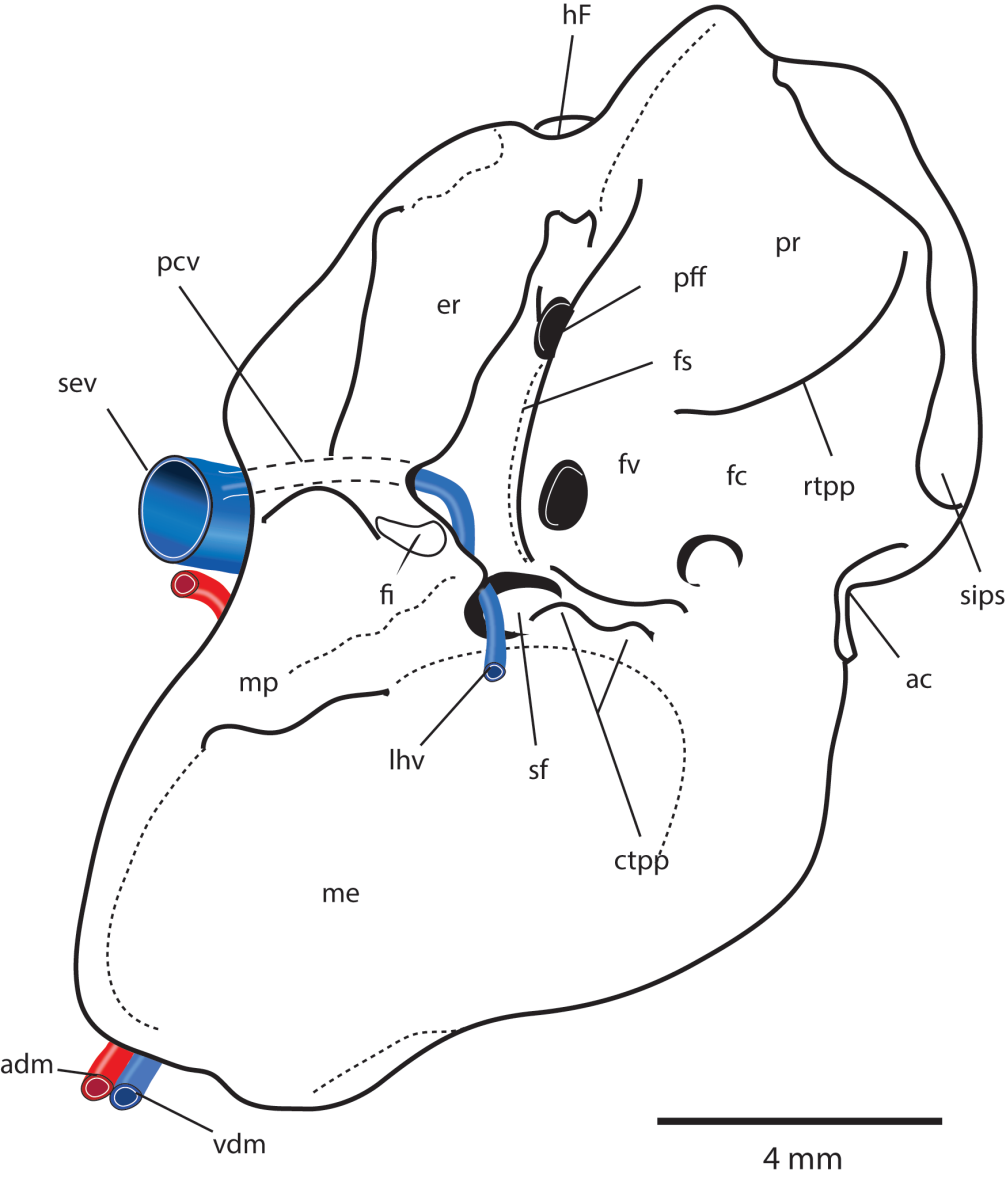
Right petrosal Type A (*Protolambda*) after Wible (1990) showing reconstruction of vasculature. Abbreviations: **ac** aqueductus cochleae; **adm** arteria diploetica magna; **cev** capsuloparietal emissary vein; **cp** crista parotica; **ctpp** caudal tympanic process of petrosal; **er** epitympanic recess; **fc** fenestra cochleae; **fi** fossa incudis; **fs** facial sulcus; **fv** fenestra vestibuli; hF, hiatus Fallopii; If, lateral flange of petrosal; **lhv** lateral head vein; It, lateral trough of petrosal; **me** mastoid exposure; **mp** mastoid process; **pc** prootic canal; **pcv** vein ofprootic canal; **pff** primary facial foramen; **pp** paroccipital process; **pr** promontorium; **ri** ramus inferior of stapedial artery; **rs** ramus superior of stapedial artery; **rtpp** rostral tympanic process of petrosal; **sa** stapedial artery; **sev** sphenoparietal emissary vein; **sf** stapedius fossa; **sff** secondary facial foramen; **sips** sulcus for inferior petrosal sinus; **spd** sulcus for perilymphatic duct; **vdm** vena diploetica magna.

**Figure 10.**
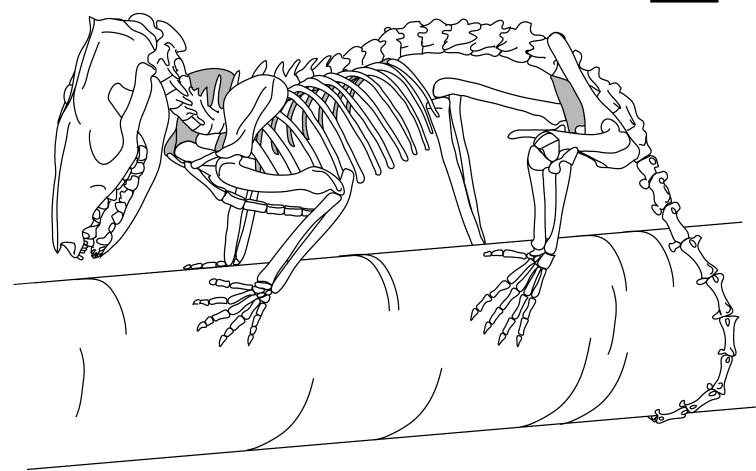
Skeleton of *Monodelphis domestica*. Scale bar is 1 cm.

**Figure 11.**
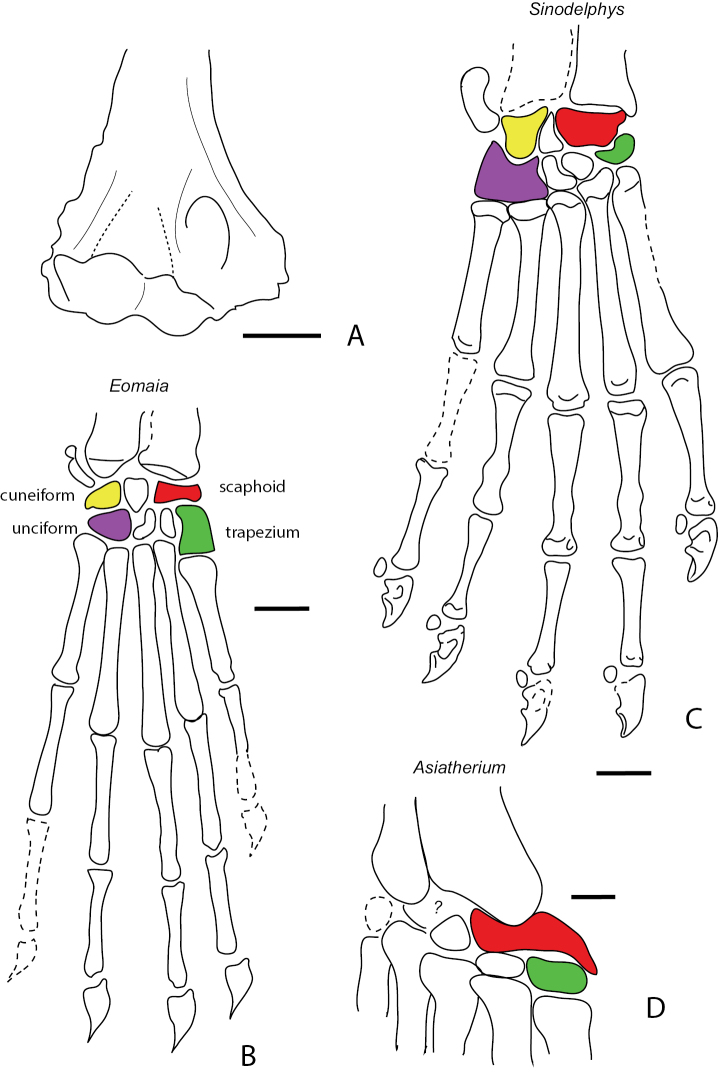
Humerus and carpi of Cretaceous metatherians. **A** Distal humerus of unidentified metatherian (cf. *Sulestes*?) from the Bissetky Locale, Uzbekistan after Chester et al. (2010) in anterior view. Carpus of the Cretaceous stem therian *Eomaia* (**B**) and the Cretaceous metatherians *Sinodelphys* (**C**) and *Asiatherium* (**D**) after Szalay and Trofimov (1996) and Luo et al. (2003). Scale bars equal 1 mm.

**Figure 12.**
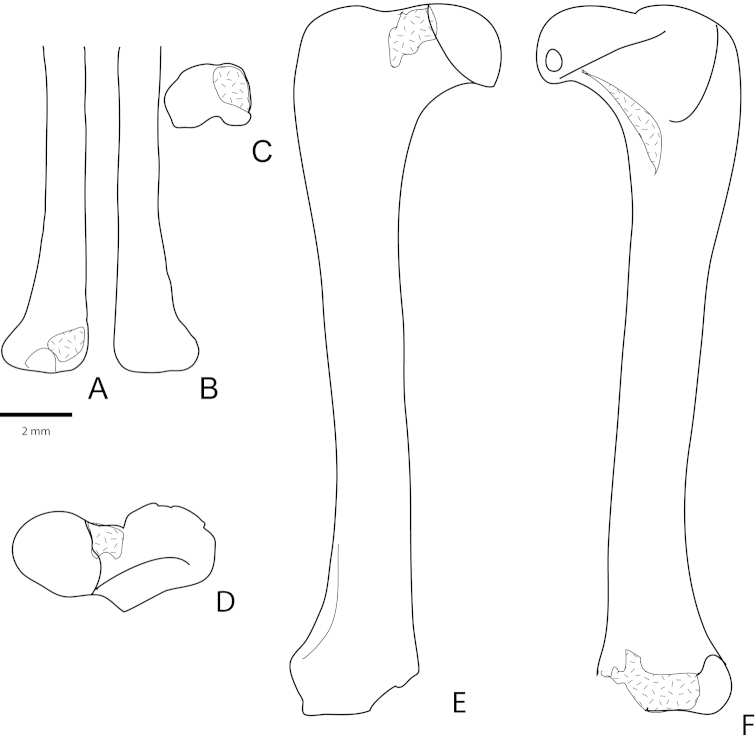
Partial femora of unidentified metatheria (cf. *Sulestes*?) from the Bissetky Locale, Uzbekistan after Chester et al. (2012). **A–C** right distal femur in anterior (**A**), posterior (**B**), and distal (**C**) views **D–F** right proximal femur of a possible unidentified metatherian in proximal (**D**), anterior (**E**), and posterior (**F**) views.

**Figure 13.**
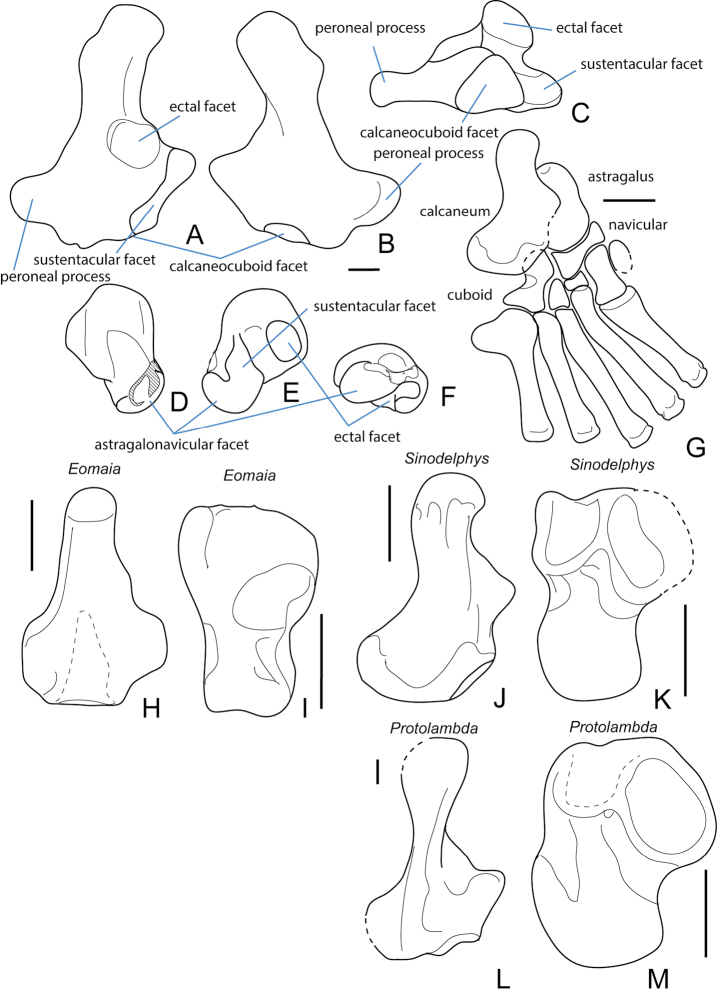
Tarsus of the Cretaceous stem therian *Eomaia* and Cretaceous metatherians. **A–F** isolated calcaneum (**A–C**) (cf. *Sulestes*?) from the Bissetky Locale, Uzbekistan in dorsal (**A**), ventral (**B**), and distal (**C**) views after Szalay and Sargis (2006). **D–F** isolated astragalus (cf. *Sulestes*?) from the Bissetky Locale, Uzbekistan in dorsal (**D**), ventral (**E**), and distal (**F**) views after Szalay and Sargis (2006). **G** articulated partial pes of *Sinodelphys* in ventral view **H–I** calcaneum and astragalus, respectively, of *Eomaia* in ventral view **J–K** calcaneum and astragalus of *Sinodelphys*, respectively, in ventral view **L–M** calcaneum and astragalus of *Protolambda*, respectively, in ventral view **G–M** are after Luo et al. (2003). Scale bars equal 1 mm.

## Dating the origin and evolution of Metatheria

### Molecular evidence

The most recent molecular clock analyses estimate the marsupial-placental split between ~140 and 190 million years ago, depending on the tree topology, calibrations, and rate models used (Bininda-Emonds et al. 2007; dos Reis et al. 2014; dos Reis et al. 2012; Meredith et al. 2011; Phillips et al. 2009; van Rheede et al. 2006). The recent study of dos Reis et al. (2012), which is the most rigorous and comprehensive yet published, estimated this split at approximately 168–178 million years ago, which corresponds to the late Early Jurassic-early Middle Jurassic. Most of these studies were conducted prior to the discovery of what is currently considered the oldest unequivocal eutherian fossil, the ca. 160 million-year-old holotype of *Juramaia* (Luo et al. 2011). If *Juramaia* is correctly identified as a eutherian, then the marsupial-placental split must have occurred prior to 160 million years ago. The dates provided by dos Reis et al. (2012) therefore comply with the age of *Juramaia*.

Many of these molecular clock studies also estimate the origin of Marsupialia. dos Reis et al. (2012) estimated marsupials to have originated 64–84 million years ago. Previous studies also reported similar age ranges. For example, Bininda-Emonds et al. (2007) estimated this divergence at 71.4–93.7 million years ago, and Meredith et al. (2011) at 67.9–97.2 million years ago. These findings generally agree that the origin of Marsupialia likely occurred in the Late Cretaceous, although there is a small possibility using the dos Reis et al. (2012) results that marsupials were a strictly post-Cretaceous radiation.

### Paleontological evidence

*Juramaia sinensis* was recently described as the oldest eutherian mammal, and therefore the oldest therian as well (Luo et al. 2011). Luo et al. (2011) estimated its age to be ca. 160 Ma based on radiometric dates of between about 159 Ma and 165 Ma for the Tiaojishan Formation, the rock unit in China in which the holotype was discovered (Liu and Liu 2005; Liu et al. 2006). More recent dates have further constrained the age of the horizon in which *Juramaia* was discovered to between 160.5–161.0 million years ago (Liu 2012). This would place *Juramaia* in the earliest part of the Late Jurassic (Gradstein et al. 2012). This is substantially older than the previously regarded oldest eutherian, *Eomaia*, from the Early Cretaceous (ca. 125 million years old) of China. It is also older than what were previously considered to be the oldest boreosphenidan taxa (*Tribactonodon* and *Aegialodon*) from the Lower Cretaceous of England (Berriasian Purbeck Group and Valangininan Wealden Group, respectively) and similar in age to the oldest australosphenidan (*Ambondro*) from the Middle Jurassic of Madagascar (Flynn et al. 1999).

The oldest proposed metatherian, *Sinodelphys szalayi*, is Early Cretaceous in age (ca. 124–131 million years old [Yang et al. 2007]). However, some workers (e.g., Averianov et al. 2010) have voiced doubts about whether this taxon has the classic metatherian dental formula, as was originally described by Luo et al. (2003). If its dental formula is different from the metatherian norm, it may mean that *Sinodelphys* is not a metatherian (see above). Disregarding *Sinodelphys*, the oldest certain metatherians are the deltatheroidans *Oklatheridium szalayi* and *Atokatheridium boreni* from the Lower Cretaceous (Albian, ca. 104–108 million years old) Antlers Formation of Oklahoma (Averianov et al. 2010; Cifelli and Davis in press; Davis et al. 2008; Kielan-Jaworowska and Cifelli 2001) and of an indeterminate genus and species from the Lower Cretaceous (Albian) Cloverly Formation of Montana (Cifelli and Davis in press). These are older than the oldest unequivocal deltatheroidans from Asia, which are *Sulestes karakshi* and *Sulestes* sp. from the Upper Cretaceous (Turonian, ca. 94–90 million years old) Bissekty Formation of Uzbekistan (Averianov et al. 2010; Kielan-Jaworowska and Nessov 1990).

The first appearance of marsupialiforms (i.e., non-deltatheroidan metatherians) in North America is near the Albian-Cenomanian boundary, at the base of the Late Cretaceous, approximately 100 million years ago (Cifelli 2004; Cifelli and Muizon 1997; Eaton 1993; Cifelli and Eaton 1987). *Arcantiodelphys*, a marsupialiform from the Cenomanian of Europe (Vullo et al. 2009), is nearly as old.

The oldest unequivocal crown marsupials appear in the early Paleocene with *Peradectes* and other taxa (Horovitz et al. 2009; O’Leary et al. 2013; Williamson et al. 2012). The South American Paleocene *Pucadelphys* was considered to be outside of Marsupialia by some workers (Horovitz et al. 2009), but within the crown clade by O’Leary et al. (2013). The latest Cretaceous North American *Glasbius* may represent a Cretaceous member of Marsupialia if it is found to be a member of, or sister taxon to, the extinct, mostly South American clade, Polydolopimorphia (Goin et al. 2003), which some workers have allied with the extant clade Caenolestidae, in Paucituberculata and other workers have allied with the extant Microbiotheria (Horovitz and Sánchez-Villagra 2003; Kirsch et al. 1997).

### Summary

Both molecular clock estimates and fossil evidence (if correctly identified) currently agree that the metatherian-eutherian split, and therefore the origin of Metatheria, occurred by 160 million years ago. Crown-group metatherians (Marsupialia) probably originated within the 10 million years before the Cretaceous-Paleogene boundary, although their oldest unequivocal fossils are from the earliest Paleocene and molecular clocks cannot completely rule out a post-Cretaceous origin. Both deltatheroidans and marsupialiforms first appear in the fossil record in the middle part of the Cretaceous, but these are minimum divergence estimates.

## Geologic setting and paleoenvironment of Cretaceous Metatheria

The oldest probable metatherian, *Sinodelphys*, comes from the Lower Cretaceous lagerstätten of the Yixian Formation of northeastern China (Tables 3–4), which has produced hundreds of relatively complete fossil specimens, including many of the famous feathered dinosaurs (Luo et al. 2003). These exceptional fossils were preserved by pyroclastic debris flows that killed and buried a diverse array of terrestrial and aquatic organisms living in and around a series of lakes surrounded by dense forests (Jiang et al. 2014). Just because *Sinodelphys* is the oldest currently recognized metatherian, however, does not mean that metatherians necessarily originated in this type of environment or even favored it during their early history. Given the extreme sampling biases affecting the recovery of early mammal fossils, it is probable that at some point in the near future an even older metatherian will be found, from a different place and paleoenvironment. What *Sinodelphys* does indicate is that early metatherians were small-bodied components of diverse ecosystems that also included dinosaurs, crocodylomorphs, amphibians, reptiles, and many different types of mammal.

Following *Sinodelphys*, the next earliest metatherians in the fossil record are the Early Cretaceous delatatheroidans *Oklatheridium* and *Atokatheridium* from eastern Oklahoma, USA (Davis et al. 2008; Kielan-Jaworowska and Cifelli 2001) (Tables 3–4), which are only slightly younger than the classic Trinity faunas from nearby north-central Texas Davis et al. 2008). These deltatheroidans were found in marginal marine deposits (Nydam et al. 1997) from the southern edge of a salient of an epeiric sea that antedated the Western Interior Seaway. They date to a time (late Aptian–early Albian; ca. 113–110 Ma [Gradstein et al. 2012] approximately 10 million years before the seaway joined with southern coastal waters to separate North America into western (Laramidia) and eastern (Appalachia) landmasses (Gates et al. 2010) near the Albian-Cenomanian boundary (about 100 Ma: Gradstein et al. 2012).

Marsupialiforms first appear in North America near the Albian-Cenomanian boundary (Cifelli et al. 2004; Cifelli et al. 1997), a time of marked changes in North American Cretaceous terrestrial ecosystems (Cifelli et al. 1997). High global temperature, an equable climate, and high global sea levels persisted through the rest of the Late Cretaceous, as these metatherians diversified into numerous lineages across North America (Herman and Spicer 1997; Huber et al. 1995; Huber et al. 2002; Miller et al. 2005). Paleoclimate models and oxygen isotope evidence support the presence of a highland-driven monsoon during the Late Cretaceous of North America (Fricke et al. 2010).

The entire North American record of Late Cretaceous metatherians (Tables 3–4), with the exception of a small fauna from the Prince Creek Formation of the Alaskan North Slope (Clemens 1992; Clemens 1995; 2003), the El Gallo Formation of Baja California (Lillegraven 1972; Lillegraven 1976), and a single tooth from the Maastrichtian of New Jersey (Grandstaff et al. 2000), are from the marginal marine to alluvial floodplain deposits exposed along the eastern margin of Laramidia, between the Sevier and Laramide highlands (ancestral Rockies) and the margin of the Western Interior Seaway. These faunas consist primarily of isolated teeth and small jaws collected through underwater screenwashing methods (Cifelli et al. 1996a). Characteristic taxa such as *Pediomys*, *Didelphodon*, *Glasbius*, and *Alphadon* have been collected in this way from well-known formations such as the Hell Creek and Judith River formations. These metatherians would have been components of dinosaur-dominated ecosystems, including some faunas that included iconic dinosaurs like *Tyrannosaurus* and *Triceratops*.

This North American record for the Mesozoic is unequaled for its sampling density, broad geographic span, and great temporal depth (Gates et al. 2010). Among the longest and most continuous records is that from the Kaiparowits Plateau of Utah, which spans the Albian–Cenomanian to late Campanian (most are summarized in Cifelli et al. [2004]), with recent additions (Eaton 2006a; Eaton 2006b; Eaton 2009; Eaton and Cifelli 2013) (Tables 3–4). Other important records are found throughout the Western Interior from West Texas and northern New Mexico to southern Alberta and Sakatchewan (Cifelli et al. 2004) with recent additions and summaries (Wilson 2005; Fox and Naylor 2006; Fox et al. 2007; Williamson and Weil 2008; Hunter et al. 2010; Wilson et al. 2010; Wilson and Riedel 2010) (Tables 3–4).

Important records of Cretaceous metatherians are also known from Asia and Europe (Tables 3–4), although these are nowhere near as diverse and well sampled as those of western North America. The deltatheroidans *Deltatheroides* and *Deltatheridium* are known from the Campanian Djadokhta Formation of Mongolia, which preserves a desert environment of enormous sand dunes and interspersed freshwater oases that was dominated by a diversity of small-to-large dinosaurs (Dashzeveg et al. 1995). Other deltatheroidans, including *Sulestes*, have been found in the Turonian Bissekty Formation of Uzbekistan, which was deposited along a floodplain bordering a saltwater sea (e.g., Averianov et al. 2010). The European taxa *Arcantiodelphys* and *Maastrichtidelphys* have been found in coastal and/or nearshore marine deposits formed on the margins of island archipelagos during a time when high sea levels flooded Europe (e.g., Martin et al. 2005; Vullo et al. 2009).

**Table 3.** Locales that have yielded Jurassic and Cretaceous metatherians.

**Europe**
Font-de-Benon quarry, Archingeay-Les Nouillers (Cenomanian, Late Cretaceous), Charente-Maritime, southwestern France (Vullo et al. 2009)
Valkenburg Member, Maastricht Formation (late Maastrichtian, Late Cretaceous), southern Limburg, The Netherlands (Martin et al. 2005)
**Asia**
Yixian Formation, China (Barremian, Early Cretaceous)
Jehol fauna (Luo et al. 2003)
Bissekty Formation, Kyzylkum Desert, Uzbekistan (Turonian, Late Cretaceous)
Bissekty local fauna (Averianov et al. 2010; Kielan-Jaworowska and Nessov 1990; Nessov 1985; 1987; 1993)
Darbasa Formation, southern Kazakhstan (Campanian, Late Cretaceous)
Grey Mesa locality (Averianov 1997)
Barun Goyot Formation, Umuni Gobi, Mongolia (Campanian, Late Cretaceous)
Udan Sayr locality (Szalay and Trofimov 1996; Trofimov and Szalay 1994)
Nemegt Formation, Omnogov, Mongolia (Maastrichtian, Late Cretaceous)
Gurlin Tsav, Mongolia (Kielan-Jaworowska and Nessov 1990; Rougier et al. 1998; 2004; Szalay and Trofimov 1996)
Djadokhta Formation, Mongolia (Campanian, Late Cretaceous)
Ukhaa Tolgod and Kholbot localities, Mongolia (Horovitz 2000; Rougier et al. 1998; 2004)
Bayn Dzak, Mongolia (Gregory and Simpson 1926; Kielan-Jaworowska 1975)
**North America**
Alaska
Prince Creek Formation, Alaska (early Maastrichtian, Late Cretaceous)
Colville River (Clemens 1995)
*Pediomys* Point local fauna (Clemens 2003; Conrad et al. 1992)
Alberta and Saskatchewan, Canada
Milk River Formation, southern Alberta, Canada (late Santonian, Late Cretaceous)
Upper Milk River (Fox 1971; 1972; Fox 2005)
Oldman Formation, southern Alberta, Canada (Campanian, Late Cretaceous)
South Saskatchewan River (Fox 1976)
Dinosaur Park Formation, southern Alberta, Canada (late Campanian, Late Cretaceous)
“Oldman Formation” (Fox 1979a; Fox 1979b; Fox 1981; Fox 1987; Fox and Naylor 2006)
Horseshoe Canyon Formation, southern Alberta, Canada (early Maastrichtian, Late Cretaceous)
Drumheller local fauna (Fox and Naylor 1986; 2006)
St. Mary River Formation, Alberta and northwestern Montana (early Maastrichtian, Late Cretaceous)
Scabby Butte local fauna (Russell 1975)
Lundbreck locality (Clemens 1979; Russell 1975)
Shell Hell locality (Hunter et al. 2010)
Scollard Formation, Alberta (late Maastrichtian, Late Cretaceous)
Trochu local fauna (Lillegraven 1969; Russell 1987; Wilson and Riedel 2010)
Frenchman Formation, Saskatchewan (late Maastrichtian, Late Cretaceous)
Wounded Knee local fauna (Fox 1989)
Gryde local fauna (Storer 1991)
quarry (Fox et al. 2007)
Montana and North Dakota
Judith River Formation (late Campanian, Late Cretaceous)
Type fauna (Lillegraven and McKenna 1986; Sahni 1972)
Two Medicine Formation (late Campanian, Late Cretaceous)
Egg Mountain (Montellano 1988; 1992)
Hell Creek Formation, Montana and North Dakota (late Maastrichtian, Late Cretaceous)
Garfield and McCone Counties, assorted localities (Archibald 1982; Lofgren 1995; Wilson 2005)
Muddy Tork local fauna, Williston Basin, Montana (Hunter et al. 1997)
Ekalaka local faunas, Montana (Hunter and Archibald 2002; Pearson et al. 2002)
Localities in the Little Missouri badlands, Montana and North Dakota (Archibald et al. 2011; Hunter and Archibald 2002; Hunter and Pearson 1996)
South Dakota
Fox Hills Formation, South Dakota (late Maastrichtian, Late Cretaceous)
Iron Lightning local fauna (Waage 1968)
Hell Creek Formation, South Dakota (late Maastrichtian, Late Cretaceous)
Joe Painter Quarry (Wilson 1983)
Eureka Quarry (Wilson 1983)
Wyoming
“Mesa Verde Formation” (late Campanian, Late Cretaceous)
Bighorn Basin local fauna (Lillegraven and McKenna 1986)
Wind River Basin local fauna (Lillegraven and McKenna 1986)
Lance Formation, Wyoming (late Maastrichtian, Late Cretaceous)
Localities near Mule Creek Junction (Whitmore 1985)
Localities in Lance Creek drainage, type Lance Formation (Clemens 1966; Lillegraven and McKenna 1986; Wilson and Riedel 2010)
Hewitt’s Foresight (Webb 2001)
Black Butte Station (Breithaupt 1982)
Ferris Formation, Wyoming (late Maastrichtian, Late Cretaceous)
Localities in Hanna Basin (Eberle and Lillegraven 1998; Lillegraven and Eberle 1999)
Utah
Cedar Mountain Formation (Albian-Cenomanian)
Mussentuchit local fauna, southern Utah (Cifelli 1993; 2004; Cifelli and Muizon 1997)
Dakota Formation fauna (late Cenomanian, Late Cretaceous)
Kaiparowits and Paunsaugunt figaus, southern Utah (Cifelli and Eaton 1987; Eaton 1993b; 2009; Eaton and Cifelli 2013)
Smoky Hollow Member, Straight Cliffs Formation (Turonian, Late Cretaceous)
Kaiparowits and Paunsaugunt figaus, southern Utah (Cifelli 1990c; Eaton and Cifelli 2013)
John Henry Member, Straight Cliffs Formation (Coniacian-Santonian, Late Cretaceous)
Kaiparowits and Paunsaugunt figaus, southern Utah (Cifelli 1990b; Eaton 2006; Eaton 2013; Eaton and Cifelli 2013)
Wahweap Formation (early-middle Campanian, Late Cretaceous)
Kaiparowits Plateau (Cifelli 1990b; Eaton 1993a; Eaton 2013; Eaton and Cifelli 2013)
Kaiparowits Formation (late Campanian, Late Cretaceous)
Kaiparowits Plateau (Cifelli 1990a; Cifelli and Johanson 1994; Eaton 2013; Eaton and Cifelli 2013)
Iron Springs Formation fauna, southern Utah (Turonian – Santonian, Late Cretaceous)
Pine Valley Mountains (Eaton 1999)
North Horn Formation, Utah (late Maastrichtian, Late Cretaceous)
Localities in North Horn Mountain and in South Dragon Canyon (Cifelli et al. 1999; Clemens 1961)
Colorado
Williams Fork Formation, Colorado (late Campanian-early Maastrichtian)
Rio Blanco local fauna (Archibald 1987)
Laramie Formation, northeastern Colorado (late Maastrichtian, Late Cretaceous)
Cheyenne Basin assemblages (Carpenter 1979; Wilson et al. 2010)
Site in Weld County (Carpenter 1979; Wilson et al. 2010)
Baja California Del Norte, Mexico
El Gallo Formation (late Campanian, Late Cretaceous) (Clemens 1980; Lillegraven 1972; Lillegraven 1976)
New Mexico
Fruitland and lower Kirtland Formation, San Juan Basin (late Campanian, Late Cretaceous)
Hunter Wash local fauna, Bisti/De-na-zin Wilderness Area, San Juan Basin (Clemens 1973; Clemens and Lillegraven 1986; Flynn 1986)
Fossil Forest, San Juan Basin (Rigby and Wolberg 1987)
Willow Wash local fauna (Flynn 1986)
Naashoibito Member, Kirtland Formation, New Mexico (late Maastrichtian, Late Cretaceous)
Alamo Wash local fauna, San Juan Basin (Flynn 1986; Williamson and Weil 2008)
Oklahoma
Antlers Formation, Texas and Oklahoma (Aptian-Albian, Early Cretaceous)
Tomato Hill local fauna (Davis et al. 2008; Kielan-Jaworowska and Cifelli 2001)
Texas
Aguja Formation, West Texas (late Campanian, Late Cretaceous)
Terlingua local fauna (Cifelli 1994; Rowe et al. 1992)
New Jersey
Marshalltown Formation, New Jersey (Campanian, Late Cretaceous)
Ellisdale Site (Grandstaff et al. 1992)

**Table 4.** Distribution of Cretaceous metatherian taxa. Numbers correspond to localities listed in Table 3.

Taxon	Localities
*Adelodelphys muizoni*	25
*Aenigmadelphys archeri*	30
*Albertatherium primum*	10
*Albertatherium secundum*	10
*Alphadon attaragos*	22, 30
*Alphadon eatoni*	32
*Alphadon halleyi*	12, 17, 18, 22, 28?, 30, 36, 38?
*Alphadon marshi*	15, 16, 19, 21?, 23, 33, 37
*Alphadon perexiguus*	38
*Alphadon sahni*	12, 17, 22, 30, 36
*Alphadon wilsoni*	15, 19, 23, 33, 36?
*Anchistodelphys archibaldi*	29
*Anchistodelphys delicatus*	27
*Apistodon exiguus*	10, 28?, 29?
?*Aquiladelphis laurae*	29
*Aquiladelphis incus*	10, 33
*Aquiladelphis minor*	10, 36
*Arcantiodelphys marchandi*	1
*Asiatherium reshetovi*	6
*Atokatheridium boreni*	38
*Bistius bondi*	36
*Dakotadens morrowi*	26
*Deltatheridium pretrituberculare*	8
*Deltatheroides cretacicus*	8
*Didelphodon coyi*	13
*Didelphodon vorax*	15, 16, 19, 20?, 23
*Ectocentrocristus foxi*	17, 36?
*Eoalphadon clemensi*	26
*Eoalphadon lillegraveni*	26
*Eoalphadon woodburnei*	26
*Eodelphis browni*	12
*Eodelphis cutleri*	12, 17
*Glasbius intricatus*	23, 37?
*Glasbius twitchelli*	16, 19
*Hatcheritherium alpha*	23
*Iqualadelphis lactea*	10, 28
*Iugomortiferum thoringtoni*	29
*Kokopellia juddi*	25
?*Leptalestes cooki*	14?, 19, 23, 33, 34, 36?
*Leptalestes krejcii*	14?, 15, 16, 19, 21, 23
*Leptalestes prokrejcii*	11, 12, 17, 18, 36
*Leptalestes toevsi*	14
*Maastrichtidelphys meurismeti*	2
*Nanocuris improvida*	15, 19, 23
*Nortedelphys jasoni*	15, 16, 19, 21, 23, 34
*Nortedelphys magnus*	15, 19, 23
*Nortedelphys minimus*	19, 23
*Oklatheridium szalayi*	38
*Pariadens kirklandi*	26
*Pariadens mckennai*	25
*Pediomys elegans*	9, 12, 15, 16, 21, 23, 35
*Protalphadon foxi*	19
*Protalphadon lulli*	21?, 22, 23, 24, 39
?*Protolambda clemensi*	12, 17, 18, 22
*Protolambda florencae*	19, 21, 23
*Protolambda hatcheri*	12?, 15, 16?, 19, 21, 23, 32, 34
*Sinbadelphys schmidti*	25
*Sinodelphys szalayi*	3
*Sulestes karakshi*	4
*Turgidodon lillegraveni*	30, 38?
*Turgidodon madseni*	30
*Turgidodon petiminis*	16
*Turgidodon praesagus*	11, 12, 17
*Turgidodon rhaister*	14?, 15, 19, 23
*Turgidodon russelli*	11, 12, 14, 17, 29?, 33, 36
*Varalphadon creber*	10, 29?
*Varalphadon crebreforme*	29
*Varalphadon wahweapensis*	29, 30
The “Gurlin Tsav Skull”	7

## Paleobiology

Body mass is significantly correlated with dental dimensions in extant mammals. As such, predictive formulae have been developed to estimate body mass in fossil metatherians. These formulae rely on the area of the dp5 (traditionally m1) as estimated by the product of length and width of the crown (Gordon 2003a; Smits and Wilson 2011). Based on estimates from Smits and Wilson (2011), the average body mass of metatherians was ~13 g during the Early- to mid-Cretaceous and increased during the Late Cretaceous to ~180 g. Body mass ranged from tiny shrew-sized (~10 g) *Anchistodelphys archibaldi*, *Anchistodelphys delicatus*, and *Leptalestes prokrejcii* to nearly ring-tailed cat-sized (~2 kg) *Didelphodon vorax*, *Didelphodon coyi*, and *Aquiladelphis incus*. For comparison, the smallest living marsupial, the long-tailed planigale (*Planigale ingrami*), weighs ~5 g, only slightly smaller than *Anchistodelphys*. The largest living marsupial, the red kangaroo (*Macropus rufus*), weighing up to ~90 kg, is an order of magnitude larger than *Didelphodon* (Nowak 1999).

Reconstructing the postures, locomotor abilities, and habitat preferences of most fossil metatherians is exceedingly difficult, because most extinct taxa are known only from isolated jaws and teeth. Nevertheless, the rare postcranial fossils of Cretaceous metatherians give some insight into their locomotory paleobiology. The small skeleton of the oldest recognized metatherian, *Sinodelphys*, possesses features seen in living mammals that climb, leading Luo et al. (2003) to hypothesize that it was scansorial and possibly arboreal. These include features of the forefoot that indicate grasping ability, such as proximal manual phalanges that are dorsally arched, large protuberances supporting large digital flexor muscles, sesamoid bones at the distal phalangeal joints, and phalangeal ratios similar to those of extant scansorial/arboreal didelphid marsupials. Luo et al. (2003) concluded that *Sinodelphys* would have been an agile, fast-moving animal capable of grasping and walking along branches, but also able to move on the ground and in small shrubs. The living *Didelphis* was suggested as a modern analog. Luo et al. (2011) later described the basal eutherian *Juramaia* as possessing similar scansorial features, suggesting that the common ancestor of metatherians and eutherians was a small, agile, climbing animal. This differs from many other Mesozoic mammals, most of which are thought to have been terrestrial.

The locomotor habits of deltatheroidans are difficult to reconstruct due to the lack of associated skeletal material for this clade. Chester et al. (2010; 2012) described several isolated metatherian humeri and femora from the Bissekty Formation (Fig. 11A), which may belong to deltatheroidans based on the fact that *Sulestes* is known from this formation. The humeri possess an elbow joint with a wide range of motion, a large muscle scar for the flexor muscles of the antebrachium, and a large medial epicondyle that would have supported enlarged muscles for grasping, features that are important for arboreal mammals. The femora (Fig. 12) have distal condyles that are unequal in size and lack a patellar groove, which are also commonly seen in climbing species. Szalay and Sargis (2006) also described isolated tarsals from Bissekty (Fig. 13) that they assigned to Deltatheroida, which they described as possessing arboreal features. Therefore, if some or all of these specimens belong to deltatheroidans, they may indicate that this clade was largely scansorial or arboreal.

Living didelphids are widely considered to be good analogs for Cretaceous non-deltatheroidan metatherians. This is somewhat problematic, however, because living didelphids are capable of a great variety of locomotor habits, and range from being mostly terrestrial (e.g., *Metachirus*) to arboreal (e.g., *Caluromys*) (Kielan-Jaworowska et al. 2004). Such variety may have also been present in Cretaceous taxa. *Asiatherium*, which along with *Sinodelphys* is the only Cretaceous metatherian represented by decent skeletal remains, lacks an anticlinal vertebra, a condition also seen in many leaping and cursorial mammals today (Kielan-Jaworowska et al. 2004). Furthermore, it has a transversely stabilized elbow joint and a slender proximal fibula, both of which are commonly seen in extant terrestrial marsupials (Szalay and Trofimov 1996). Therefore, it appears as if *Asiatherium* was well adapted for a terrestrial lifestyle, and lacks features (like those of *Sinodelphys*) expected in climbing species. Isolated tarsals from North America assigned to pediomyids (Fig. 13) have also been described as lacking arboreal specializations, such as the classic tarsal shapes that permit the foot to invert while climbing, and are therefore thought to indicate more terrestrial habits (Szalay 1994a).

There have been some suggestions that some latest Cretaceous metatherians from North America may have occupied an unusual aquatic habitat. Based on the morphology of isolated tarsal bones, Szalay (1994a) suggested that stagodontids had remarkably flexible feet that could rotate in a wide arc, which he regarded as a characteristic of aquatic animals. However, Fox and Naylor (2006) criticized this hypothesis, noting that flexible feet are also commonly seen in non-aquatic taxa such as climbers and gliders. They also argued that Szalay (1994a) could not confidently assign isolated tarsals to stagodontids, or other metatherian taxa, as these taxa are usually known entirely from teeth and jaws. Therefore, even if these tarsals exhibited unequivocal aquatic adaptations, it is uncertain if they belonged to metatherians or other therian mammals that coexisted in the same ecosystems. In an abstract, Longrich (2004) argued that the morphology of supposed stagodontid caudal vertebrae and their abundance in fluvial deposits supported an aquatic lifestyle. Fox and Naylor (2006) also criticized this hypothesis, noting again the difficulty of assigning postcranial bones to stagodontid taxa known only from teeth and also pointing out that many other mammals and dinosaurs are commonly found in the same fluvial deposits, likely because they were transported by rivers and not because they all lived in the water.

Compared to locomotion, reconstructing the diet of Cretaceous metatherians is more straightforward, because tooth morphology can often give clear insights into feeding behavior and preferences (Clemens 1966; Clemens 1968a; b; Davis 2007; Fox and Naylor 2006; Gordon 2003b; Wilson 2013). However there are few quantitative analyses of Cretaceous metatherian tooth shape (Butler 1990; Gordon 2003b; Wilson 2013). The earliest North America Albian–Cenomanian metatherian faunas exhibited fairly conservative molar morphologies (see Fig. 5), with a low range of tooth size and shape, which probably reflects a relatively narrow range of mostly faunivorous diets (Cifelli 2004). A much greater range in tooth size (reflecting a greater range of body sizes) and shape (reflecting a greater range of diets) was established among North American metatherians by the latest Santonian and persisted throughout the remainder of the Late Cretaceous (Cifelli 2004; Davis 2007).

Deltatheroidans were probably predominantly carnivorous. They are relatively large in size, exhibit enlarged temporalis muscle attachment sites on the skull, possess large canines, and have hypertrophied upper and lower molar shearing blades accentuating postvallum/prevallid shear (enlarged paracristids on the lower molars and metacrista on the upper molars) and reduced talonid basins reflecting de-emphasis of crushing (Fox et al. 2007; Muizon and Lange-Badré 1997; Wilson 2013; Wilson and Riedel 2010) (Fig. 5). All of these features indicate that deltatheroidans incorporated a large amount of vertebrate prey in their diet, potentially other small mammals as well as small lizards and amphibians.

Small-bodied Cretaceous marsupialiforms were probably mostly insectivorous, based on their fairly conservative molar structure and teeth that appear well suited for both shearing and crushing. These taxa include various basal marsupialiforms (such as “alphadontids”), small pediomyids, and potential Cretaceous herpetotheriids and peradectids (Fig. 5). In general, these taxa are probably comparable to small-bodied living didelphids, which are mainly insectivorous, but also include some meat in their diets. Larger-bodied Cretaceous marsupialiforms are probably analogous to larger-bodied living didelphids, which are opportunistic feeders that incorporate a range of foodstuffs in their diets, including insects, vertebrates, carrion, fruit, and other items (Nowak 1999). Their small, sharp and high-cusped teeth that are well-adapted for piercing the thick, brittle exoskeletons of small arthropods (Evans and Sanson 1998). Similar teeth are seen in many Cretaceous marsupialiforms.

Large pediomyids are also probably roughly analogous to mid-to-large-sized extant didelphids, but exhibit somewhat more pronounced adaptations for crushing (larger protocone and talonid basin) relative to most other Cretaceous metatherians. Taxa such as *Pediomys* and *Protolambda* (Fig. 5) possess strong shearing crests with pronounced postprotocrista that extend completely across the distal margin of the tooth to the buccal margin, creating pronounced double-rank postvallum/prevallid shear, but they have reduced the size of the stylar shelf mesially, greatly reducing prevallum-postvallid shear. These taxa also possess large and broadly expanded talonid basins for occlusion with the large protocones, reflecting a large capacity for crushing.

Stagodontids exhibit some of the most unusual premolars among Cretaceous metatherians, indicating that they had a distinctive diet. These taxa are particularly large from their first appearance in the Albian-Cenomanian (*Pariadens*) (Cifelli and Eaton 1987) to the end of the Cretaceous, consistently representing the largest metatherians in their local faunas. The molars exhibit features related to enhanced postvallum/prevallid shearing, which suggests a carnivorous diet. The premolars are inflated and blunt, and the upper and lower sets broadly occluded when the jaws were shut, which is a hallmark of durophagy in many living mammals (Clemens 1968a; b; Fox and Naylor 2006). It has been suggested that stadogontids may have fed on shelled molluscs (Clemens 1979), or even used their inflated premolars to crush bone, as in the extant Tasmanian devil (*Sarcophilus*) and many placentals (Clemens 1968a; b).

The other Cretaceous metatherians with a highly unusual dentition are the species of *Glasbius* (Figs 3, 5). Their low molar crowns and reduced, bulbous cusps likely reflect an increase in crushing and grinding ability, at the expense of a loss of the shearing ability seen in many close relatives. It is widely hypothesized that *Glasbius* favored high-calorie plant material such as seeds and fruits, rather than foliage, insects, or vertebrate prey (Kielan-Jaworowska et al. 2004; Wilson 2013). It may be that *Glasbius* was one of the earliest mammals with a frugivorous (or nearly frugivorous) diet. Such a diet would have been suitable for the high energy requirements of a small (<50 gram) metatherian, and would have been possible in a Late Cretaceous world where flowering plants were diversifying (Kielan-Jaworowska et al. 2004).

## Cretaceous paleobiogeography

**Figure 14.**
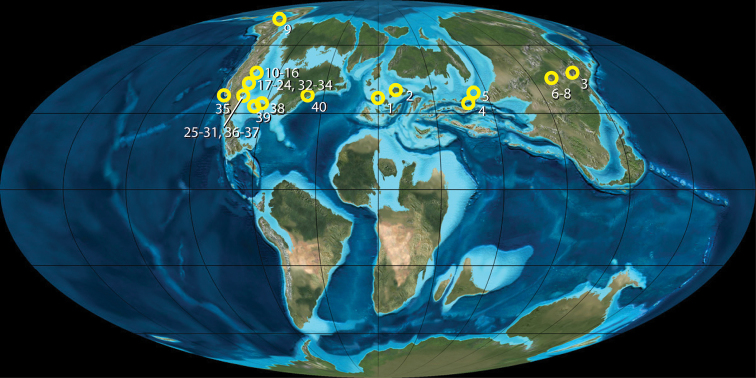
World map showing Late Cretaceous metatherian locales. Numbering of locales corresponds to the Cretaceous metatherian localities listed in Table 3.

As with many fossil groups, the greatest handicap in studying the biogeography and distribution of Cretaceous metatherians is their patchy fossil record (Fig. 14; Tables 3–4). Only Late Cretaceous North America boasts a diverse, densely sampled, relatively continuous record of metatherian fossils over the course of many millions of years. Even this record, however, is strongly biased, as metatherians are represented almost entirely by isolated teeth and jaws, most of which have been collected with a single technique (screenwashing) and found only in those settings that favor the preservation of small vertebrates. Furthermore, because of the uneven nature of fossil preservation and unequal collecting efforts, certain metatherian faunas (e.g., those of the Maastrichtian portion of the Hell Creek Formation of Montana and nearby states; Table 3) have been much better sampled than others (e.g., Hanna Basin, Cheyenne Basin, Denver Basin, San Juan Basin, Big Bend region).

Regardless, this imperfect North American record is considerably better than the very poorly sampled Asian and European Late Cretaceous records. Metatherians clearly lived in these regions, but very few specimens are known (e.g., only two Cretaceous metatherian taxa are known from Europe, one from only a single tooth). Unequivocal metatherians have yet to be found in the Cretaceous of Africa (Krause [2001] reported a tooth fragment of a metatherian from the Late Cretaceous of Madagascar, but this identification was refuted by Averianov et al. [2003]), Antarctica, Australia, and South America. This may be a genuine signal, and indeed it is widely considered that metatherians invaded the southern continents after the K-Pg boundary (e.g., Clemens 1968b; Rage 1981; Rage 1986). Alternatively, it could be that metatherians lived in some of these regions during the Cretaceous (perhaps with very low diversity) but remain unsampled (see Rana and Wilson 2003).

Because of the pervasiveness of sampling bias, it is very difficult to rigorously study how metatherians were distributed across the Cretaceous globe and how intercontinental dispersals and continental breakup may have affected their evolution. Many previous authors have proposed complex, and in some cases grand, biogeographic narratives based on very limited fossil evidence, sometimes the discovery of a single new tooth. Such scenarios are tempting to consider, but exceedingly difficult to test. Undoubtedly, as the metatherian fossil record improves and metatherian phylogeny becomes better resolved, more explicit cladistic biogeographic methods will enable biogeographic patterns to be proposed and tested with increased rigor. For the time being, however, we are restricted to discussing general biogeographic patterns based on a fairly literal reading of the fossil record, with a skeptical eye always on the lookout for sampling biases.

One particular question of interest in the literature has been the ancestral “area of origin” of Metatheria. For much of the last century, it was explicitly stated or implicitly assumed that metatherians, including crown marsupials, originated and first diversified in North America, based on the great diversity of Late Cretaceous metatherian fossils (e.g., Clemens 1979; Lillegraven 1974; Rose 2006). More recently, the discovery of what is currently the oldest known metatherian, *Sinodelphys*, in the Early Cretaceous of China (Luo et al. 2003), along with the discovery of close metatherian outgroups in China (Luo et al. 2011) and the description of numerous deltatheroidan fossils from across Asia (Averianov et al. 2010; Rougier et al. 1998; Rougier et al. 2004), has led some workers to consider Asia to be the most likely “center of origin.” For example, Luo et al. (2003) presented a biogeographic scenario in which metatherians diverged from eutherians in Asia prior to the Early Cretaceous and then experienced one or more dispersals to North America, where they radiated into a diversity of species in the Late Cretaceous.

Such a scenario is certainly congruent with the known fossil record, but as outlined above, this fossil record is biased. Just because the oldest and most basal currently known metatherian is from Asia is not particularly strong evidence that the clade must have originated there. *Sinodelphys* was found in the Yixian Formation, as part of a spectacularly preserved fauna that was killed, transported, and buried quickly by pyroclastic debris flows (Jiang et al. 2014). Such preservational modes are rare, which explains why small-bodied mammals and feathered dinosaurs are not commonly found elsewhere in the Mesozoic. *Sinodelphys*, therefore, probably provides a lucky glimpse into early metatherian anatomy and paleoenvironments, but not necessarily an accurate picture of the ancestral metatherian. This common ancestor could have lived almost anywhere, given the notoriously sporadic global fossil record of the late Early–early Middle Jurassic, when metatherians are estimated to have originated (see above). Also, the post-Jurassic, pre-Albian record of North America is virtually nonexistent (Cifelli et al. in press).

Once metatherians originated, they spread to various regions around the globe. The diversity of Cretaceous metatherians in North America, and to a lesser extent in Asia and Europe, indicates that most of their early diversification was centered in the northern continents. The lack of unequivocal Cretaceous metatherians in the southern continents is potentially due to sampling biases, as the Gondwanan latest Cretaceous fossil record is nowhere near as densely preserved or sampled as that in the northern continents. But, assuming that the lack of southern metatherian fossils either represents genuine absence or extreme rarity compared to the northern continents, then the Cretaceous metatherian radiation can be considered a predominantly Holarctic story. It is worth noting that Case et al. (2005) argued that there was a radiation of metatherians in the equatorial region of the Americas that gave rise to many Late Cretaceous taxa such as *Glasbius* and *Hatcheritherium*, which were dispersing into South America prior to the end of the Cretaceous. Although provocative, this scenario is not currently supported by the fossil record, as there is neither fossil evidence of metatherians in South America prior to the end of the Cretaceous, nor any Late Cretaceous metatherian fossils from Central America. The most southerly metatherian fossils from the Late Cretaceous of North America (e.g., Baja del Norte, Mexico; San Juan Basin, New Mexico; Big Bend, Texas) contain taxa that are similar to those found in faunas from higher latitudes (Cifelli 1994; Lillegraven 1972; Rowe et al. 1992; Williamson and Weil 2008).

There has been intense debate in the literature regarding how metatherians spread across the northern continents during their Cretaceous evolution. Several dispersal events probably occurred, but number and timing of these events is uncertain, due to the incomplete metatherian record, the lack of radiometric dates for most fossils, and poor resolution of metatherian phylogeny. At least one inter-continental interchange event must explain the distribution of deltatheroidans in North America and Asia (e.g., Averianov et al. 2010; Davis et al. 2008; Rougier et al. 2004; Wilson and Riedel 2010), another must account for the presence of the Asian *Asiatherium* interspersed among North America taxa in the phylogeny (Williamson et al. 2012), and one or two others are necessary to explain how the European taxa *Maastrichtidelphys* (Martin et al. 2005) and *Arcantiodelphys* (Vullo et al. 2009) are nested among North American (and possibly Asian) taxa in the phylogeny. The issues surrounding Deltatheroida and the European taxa deserve further comment.

Deltatheroidans were first discovered in the Late Cretaceous of Mongolia by the American Museum of Natural History’s Central Asiatic Expeditions in the 1920s (Gregory and Simpson 1926), and later numerous additional specimens were found in Mongolia by the Polish-Mongolian expeditions (e.g., Butler and Kielan-Jaworowska 1973; Kielan-Jaworowska 1975) and the Mongolian Academy of Sciences-AMNH expeditions (e.g., Rougier et al. 1998; Rougier et al. 2004) and in Uzbekistan by joint Uzbek/Russian/British/American/Canadian expeditions (e.g., Averianov et al. 2010; Nessov 1985). Because of the number and diversity of specimens from Asia, it was long held that deltatheroidans originated on this continent.

However, more recent discoveries show that early deltatheroidans were present in the middle Cretaceous of North America (e.g., Davis et al. 2008; Kielan-Jaworowska and Cifelli 2001) and persisted on this continent until the very latest Cretaceous (e.g., Fox 1974; Wilson and Riedel 2010). Davis et al. (2008) suggested that deltatheroidans may have originated in North America and dispersed to Asia, but regarded this hypothesis as extremely tentative because of the incomplete fossil record of the group. Wilson and Riedel (2010) used the results of a phylogenetic analysis, which found distinct Asian and North American deltatheroidan subclades, to instead hypothesize that the clade originated in Asia in the middle Cretaceous and made a single dispersal to North America. Although this scenario is compatible with the cladogram they reported, it is not consistent with the phylogenetic results of Williamson et al. (2012), who found various North American and Asian deltatheroidans interspersed with each other on their cladogram. This highlights the critical role of phylogenetic analyses in predicting the area of origin and number of dispersals for deltatheroidans. With different tree topologies currently implying different paleobiogeographic scenarios, all that can currently be said with confidence is that Deltatheroida was a long-lived clade that included Asian and North American members, so there must have been some interchange between these continents during the middle-Late Cretaceous.

In terms of the European Cretaceous metatherians, these taxa are so rare, and their phylogenetic positions are so poorly resolved, that it is difficult to comment on their biogeographic implications. Vullo et al. (2009) discussed how the discovery of *Arcantiodelphys* in the Cenomanian of France supports the idea of Europe being part of a “wide Euramerican continental province” on which marsupialiforms initially radiated. At a minimum, this taxon indicates that Europe was part of the early evolutionary story of metatherians. However, the phylogenetic analysis by Vullo et al. (2009) recovered *Arcantiodelphys* in a large polytomy, whereas it was excluded from Williamson et al.’s (2012) comprehensive phylogeny because of its incompleteness, meaning that it is uncertain which taxa are the closest relatives of this rare European early metatherian. Therefore, it is difficult to hypothesize about potential dispersal events or other biogeographic scenarios that may explain Europe’s role in early metatherian diversification, a point recognized by Vullo et al. (2009).

Similarly, Martin et al. (2005) described the European *Maastrichtidelphys* as providing evidence for high-latitude dispersal routes between North America and Europe during the latest Cretaceous. This interpretation hinges on their identity of *Maastrichtidelphys* as a close relative of the herpetotheriids, known mostly from the post-Cretaceous. Williamson et al. (2012), however, recovered *Maastrichtidelphys* in a large polytomy with Pediomyidae and many taxa traditionally referred to as “peradectids,” but distantly related to herpetotheriids. Therefore, it is also unclear which taxa may be the closest relatives to *Maastrichtidelphys*, which leaves open the question of when, and between where, any potential dispersal events may have occurred. High-latitude dispersal in the latest Cretaceous is certainly plausible, and there is some evidence from the fossil record of dinosaurs and other organisms that this likely occurred, particularly between North America and Asia (e.g., Sereno 2000). But testing whether mammals also employed such routes will require much better fossil sampling and better resolved phylogenies.

Metatherian faunas also provide an excellent opportunity to investigate Late Cretaceous provincialism within North America (Gates et al. 2010). Numerous workers have argued that faunas along the eastern edge of Laramidia (western North America) provide evidence for distinct northern and southern provinces in western North America during the late Campanian and potentially the late Maastrichtian (Davis 2007; Gates et al. 2010; Lehman 1997; Sampson et al. 2010; Sloan 1969; Weil 1999), possibly due to dispersal barriers caused by high sea levels fragmenting landmasses and/or mountain ranges (Gates et al. 2012; Sampson et al. 2010). Although largely based on the distribution of dinosaurs, these studies suggest that northern-southern differences extend to metatherians, particularly that pediomyids and stagodontids are typically abundant and diverse in northern late Campanian faunas, but rare or absent in approximately contemporaneous southern faunas (Cifelli et al. 2004; Clemens 1973; Davis 2007; Rowe et al. 1992).

Yet again, however, sampling biases may be influencing the above conclusions (Newham et al. 2014; Vavrek and Larsson 2010). Most representative metatherian samples, especially for the Maastrichtian but to a lesser extent the Campanian, are from the northern part of Laramidia (Cifelli et al. 2004; Wilson et al. 2010). A small Maastrichtian record from the latest Cretaceous of New Mexico documents the presence of pediomyids and has a relatively high abundance of the small, dentally-specialized metatherian *Glasbius* (Williamson and Weil 2008), a taxon present in northern latest Cretaceous assemblages (Archibald 1982; Cifelli et al. 2004; Wilson 2005). This reveals two things which give a more nuanced picture of possible provincialism: 1) that at least some latest Cretaceous metatherian taxa like pediomyids were geographically widespread in the Western Interior of North America, perhaps arguing against provincialism; 2) that beyond simple presence-absence of taxa, there may have been some differences in relative abundance in northern and southern faunas that could reflect biogeographic provincialism to some degree (Wilson et al. 2010). Refined dating techniques that will allow increasingly precise correlations among Late Cretaceous vertebrate faunas of Laramidia (Roberts et al. 2005) and increased sampling of these faunas should lead to a better understanding of Cretaceous metatherian biogeography, and help clarify what the patterns may reveal about larger biogeographic provincialism (or lack thereof) during the waning of the Cretaceous.

Finally, metatherians may give some insight into community structure and assembly on small time scales and in local geographic regions. Precise dating and dense sampling of successions of faunas in smaller areas has revealed that presence/absence and relative abundances of metatherian taxa may have fluctuated on short time scales (ca. 250 Ka) (Wilson 2005; 2014), and these changes in faunal composition may be related to climatic perturbations. Other areas show what appear to be relatively large changes in contemporaneous mammalian faunal composition, including metatherians, over geographically small areas (Eaton and Cifelli 2013), suggesting that faunal composition is closely tied to local environmental conditions.

## Macroevolution, diversification, and extinction patterns

**Figure 15.**
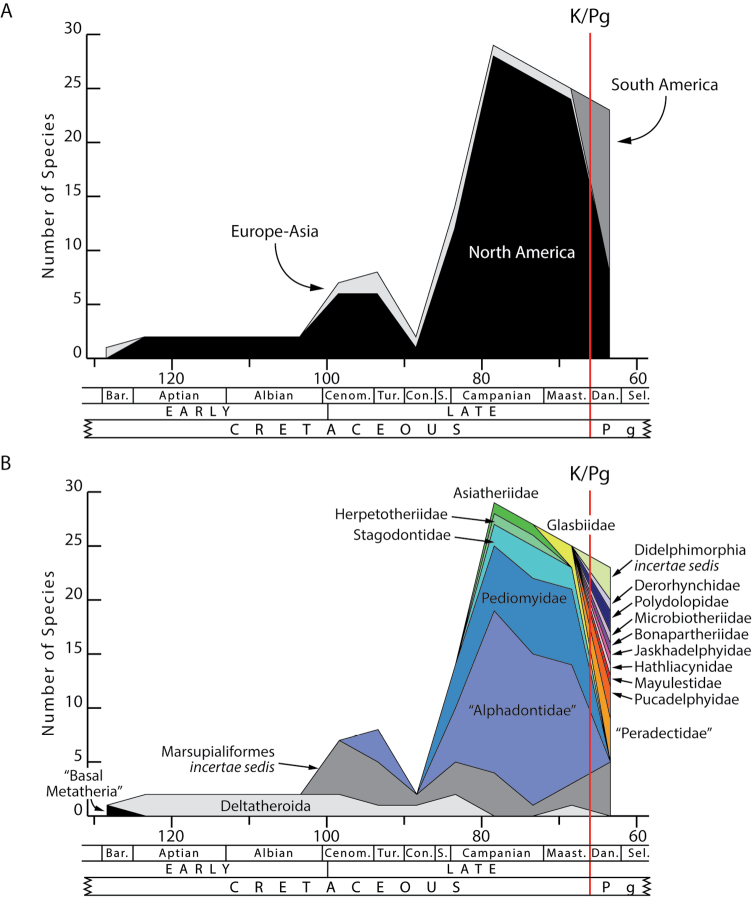
Taxonomic richness of Cretaceous and earliest Paleocene metatherians (see Tables 3–4, Suppl. materials 4–5 for data used to calculate values).

The Cretaceous record of metatherians includes a total of 68 valid species (one basal metatherian, six deltatheroidans, and 61 stem marsupialiforms) from the Barremian through Maastrichtian of Europe, Asia, and North America. In Tables 3–4, we compiled age and locality data for these Cretaceous species, as well as 22 early Paleocene (Danian) species that we considered valid (Table 3). To assess broad-scale patterns in these data, we plotted species richness through time relative to geography and higher-level taxonomy (Fig. 15). Given that the Cretaceous fossil record of mammals suffers from temporal and geographic sampling biases (see discussion above), we hope that these diversity patterns (i) will be interpreted cautiously and (ii) will serve to stimulate field research in intervals and regions of apparent low diversity.

Metatherian richness during the late Early Cretaceous (Barremian–Albian) was paltry, never exceeding two species in any time bin. Taxa included the basal metatherian *Sinodelphys szalayi* from the Barremian of China and the deltatheroidans *Atokatheridium boreni* and *Oklatheridium szalayi* from the Aptian/Albian of North America. These two deltatheroidans are the most abundant boreosphenidans in the Tomato Hill local fauna (Davis et al. 2008), but deltatheroidans are typically very rare components of younger Cretaceous mammalian faunas (e.g., Archibald and Averianov 2005; Wilson and Riedel 2010). In the early Late Cretaceous (Cenomanian–Coniacian), metatherian richness rose to a modest eight species, due to the first appearance of stem marsupialiforms, most notably “alphadontids”. As demonstrated in the local faunas of southwestern Utah, stem marsupialiforms had also become numerically abundant in some regions by the early Late Cretaceous, although usually not as abundant as multituberculates (Eaton and Cifelli 2013). Then, in the late Late Cretaceous (Santonian–Maastrichtian), a mainly North American radiation further increased the diversity of “alphadontids” and brought the first appearances of pediomyids in the Santonian, stagodontids and herpetotheriids in the Campanian, followed by glasbiids in the late Maastrichtian. Metatherian richness peaked at 29 species in the Campanian, coinciding with the tail end of the Cretaceous Terrestrial Revolution (KTR).

The KTR has been interpreted as a significant episode in the evolution of terrestrial biotas (125–80 Ma) in which the taxonomic diversification of angiosperms and the resulting new food resources spurred co-evolutionary radiations of insects and some terrestrial vertebrates (e.g., herbivorous dinosaurs; Lloyd et al. 2008). Wilson et al. (2012b), however, suggested that perhaps a more important evolutionary event occurred in the Late Cretaceous when angiosperms became ecologically diverse, abundant on the landscape, and acquired some key physiological traits (leaf hydraulic capacities) (Feild et al. 2011; Lupia et al. 1999; Wing and Tiffney 1987). Multituberculate mammals, for example, underwent an adaptive radiation at this time possibly in response to these changes in angiosperm local floras (Grossnickle and Polly 2013; Wilson et al. 2012b). In contrast, metatherians declined in richness to 25 species leading up to the K-Pg boundary (Fig. 15) and, along with other non-multituberculates, declined in morphological disparity (Grossnickle and Polly 2013). In light of how well sampled this interval is relative to other parts of the Cretaceous (see Cifelli et al. 2004), it would appear that the richness pattern is evolutionarily meaningful for at least North America and thus supports the claim by Kielan-Jaworowska et al. (2004:427) that “marsupials declined in generic diversity during the Campanian–Maastrichtian of North America.” Moreover, most recent molecular studies indicate that ordinal-level diversification within marsupials, in contrast to placentals, did not occur during the late Late Cretaceous but well within the Cenozoic (dos Reis et al. 2012; Meredith et al. 2011; but see Beck 2008; Bininda-Emonds et al. 2007). Despite the slight downtrend in stem marsupialiform richness and the low likelihood of marsupial diversification during the late Late Cretaceous, metatherians remained numerically abundant members of mammalian local faunas in North America. The record from Europe and Asia is less clear, but the Cenomanian–Coniacian eutherian-dominated mammalian local faunas of middle Asia (Uzbekistan) sharply contrast with the pattern from North America (Archibald and Averianov 2005) and speak to a biogeographically complex story that remains largely untold.

Across the K-Pg boundary, metatherian richness dropped from 25 species in the late Maastrichtian to 23 in the early Paleocene (Danian). However, this rather modest dip aggregates two very divergent patterns: in North America, richness plummeted to 8 species (66% decline), while it surged to 15 species and at least nine families in South America. A detailed review of this Paleogene radiation of South American stem marsupialiforms and marsupials is beyond the scope of this review, but see Gelfo et al. (2009), Goin et al. (2009), and Woodburne et al. (2013) for relevant summaries. Meanwhile, the mammalian transition across the K-Pg in North America has been well documented in the Hell Creek area of northeastern Montana (Archibald 1982; Clemens 2002; Lofgren 1995; Wilson 2005; 2013; 2014). Metatherians and multituberculates predominate the succession of latest Cretaceous (Lancian NALMA) local faunas from this study area, in terms of richness, relative abundances, and dental shape disparity. Metatherians make up 12 of the 31 mammalian species (4 alphadontids, 5 pediomyids, 1 stagodontid, 1 glasbiid, 1 deltatheroidan) and between 35% and 60% of all mammalian specimens found in the Hell Creek Formation, which in the study area corresponds to ca. the last 2 my of the Cretaceous. Their disparity of molar forms, which is a proxy for range of diets, is also more than twice that of eutherians from this interval. Although local richness and turnover of metatherians remained stable through the Hell Creek Formation, an apparent downturn in relative abundances of metatherians during the last 500 ky of the Cretaceous (Wilson 2014) coincides with a regional warming episode followed by a cooling episode and the K-Pg event (Tobin et al. 2012; Wilf et al. 2003).

At the K-Pg boundary, metatherians were nearly wiped out in the local section (92%); there is only a single metatherian species, *Thylacodon montanensis* (=*Peradectes* cf. *Peradectes pusillus*; Williamson et al. 2012), documented in the earliest Paleocene (early Puercan North American Land Mammal Age) local faunas of this study area, and some debate remains as to whether it was a local survivor or an immigrant that arrived immediately after the K-Pg boundary (see Williamson et al. 2012; Wilson 2013, 2014). In either case, *Thylacodon montanensis*, despite being the only metatherian species, was the third most abundant taxon in early Puercan local faunas (18% of specimens), and was among the few opportunistic generalists that appears to have thrived in this post-K-Pg disaster interval (‘bloom taxon’ see Erwin 1998). However, at least locally, metatherians were a ‘dead clade walking’—they were quickly marginalized by eutherian mammals in subsequent Puercan local faunas and never recovered previous levels of diversity in North America.

### Phylogenetic evidence for macroevolutionary patterns

Reconstruction of the phylogenetic relationships of Cretaceous and Paleogene metatherians is important for understanding the pattern of metatherian survivorship across the K-Pg boundary and the origin of the crown-clade members of Metatheria, the Marsupialia. North American metatherian faunas underwent a catastrophic decline at the boundary. The K-Pg boundary section of eastern Montana, which contains the most intensely studied latest Cretaceous terrestrial vertebrate fauna in the world, shows a drop from 11 species in the uppermost Cretaceous Hell Creek Formation to only one, *Thylacodon montanensis*, in the lowest Paleocene Tullock Formation (Williamson et al. 2012; Wilson 2014). Some previous workers have suggested that *Thylacodon* (often referred to as “*Peradectes*,” but see Williamson et al. 2012) is closely related to “*Alphadon*” or other North American metatherian taxa (e.g., Clemens 2010; Wilson 2014) and thus represents a “resident taxon.” However, our phylogenetic analysis does not support such a close relationship between *Thylacodon* and any Cretaceous metatherian taxa. Nevertheless, our analysis indicates that there were at least four boundary crossings, and perhaps as many as 13 depending on the resolution of polytomies. The Paleogene taxa Herpetotheriidae and “Peradectidae sensu lato” probably originated in the Late Cretaceous. Only two clades, the one containing *Glasbius* and *Roberthoffstetteria* and the other containing *Ectocentrocristus* and Herpetotheriidae, show well resolved relationships with sister taxa on either side of the K-Pg boundary. However, the taxa bracketing the boundary are either found on different continents (*Glasbius*, *Roberthoffstetteria*) or are separated by a significant temporal gap (*Ectocentrocristus*, *Golerdelphys*). This analysis supports the conclusion that metatherians underwent a profound extinction event at the end of the Cretaceous.

## Supplementary materials

Supplementary material 1 List of taxa and characters used in phylogenetic analysis. Data type: (measurement/occurence/multimedia/etc.) This dataset is made available under the Open Database License (http://opendatacommons.org/licenses/odbl/1.0/). The Open Database License (ODbL) is a license agreement intended to allow users to freely share, modify, and use this Dataset while maintaining this same freedom for others, provided that the original source and author(s) are credited.Thomas E. Williamson, Stephen L. Brusatte, Gregory P. Wilson

Supplementary material 2 Taxon-Character Matrix with characters ordered (TNT file). Data type: Matrix. This dataset is made available under the Open Database License (http://opendatacommons.org/licenses/odbl/1.0/). The Open Database License (ODbL) is a license agreement intended to allow users to freely share, modify, and use this Dataset while maintaining this same freedom for others, provided that the original source and author(s) are credited.Thomas E. Williamson, Stephen L. Brusatte, Gregory P. Wilson

Supplementary material 3 Characters in common on the most parsimonious trees diagnosing the nodes on the strict consensus tree in Figure 6. Data type: Characters. Explanation note: Characters in common on the most parsimonious trees diagnosing the selected nodes on the strict consensus tree resulting from the analysis run with characters ordered. This dataset is made available under the Open Database License (http://opendatacommons.org/licenses/odbl/1.0/). The Open Database License (ODbL) is a license agreement intended to allow users to freely share, modify, and use this Dataset while maintaining this same freedom for others, provided that the original source and author(s) are credited.Thomas E. Williamson, Stephen L. Brusatte, Gregory P. Wilson

Supplementary material 4 Temporal ranges of Cretaceous metatherian taxa used to calculate taxonomic richness of Metatheria. Data type: Range. Explanation note: Temporal ranges of Cretaceous metatherian taxa used to calculate taxonomic richness of Metatheria (Suppl. material 5). Data were compiled from the Paleobiology Database (PBDB; http: //fossilworks.org/?a=home), Kielan-Jaworowska et al. (2004), Woodburne et al. (2004), Williamson et al. (2012), Tables 3–4, and based on the timescale of Ogg et al. (2004). This dataset is made available under the Open Database License (http://opendatacommons.org/licenses/odbl/1.0/). The Open Database License (ODbL) is a license agreement intended to allow users to freely share, modify, and use this Dataset while maintaining this same freedom for others, provided that the original source and author(s) are credited.Thomas E. Williamson, Stephen L. Brusatte, Gregory P. Wilson

Supplementary material 5 Data used to calculate taxonomic richness for Metatherian shown in Figure 15. Data type: Range. Explanation note: Data used to calculate taxonomic richness for Metatheria shown in Figure 15. This dataset is made available under the Open Database License (http://opendatacommons.org/licenses/odbl/1.0/). The Open Database License (ODbL) is a license agreement intended to allow users to freely share, modify, and use this Dataset while maintaining this same freedom for others, provided that the original source and author(s) are credited.Thomas E. Williamson, Stephen L. Brusatte, Gregory P. Wilson
