# Supplementary material for: The origin and early evolution of metatherian mammals: the Cretaceous record
Source: Zookeys. 2014 Dec 17;(465):1–76. doi: 10.3897/zookeys.465.8178 (PMC4284630; doi:10.3897/zookeys.465.8178)
Supplement: Supplementary material 1 — List of taxa and characters used in phylogenetic analysis. [file zookeys-465-001-s001.docx]

**Appendix 1.** Taxon Character Matrix, modified from Williamson et al. (2012).

**New characters: 7, 15, 51, 52, and 68.**

**Changes to scoring from taxon-character matrix of Williamson et al. (**[**2012**](#_ENREF_118)**)(using revised character numbering):**

*Glasbius twitchelli* Character 60 (from ? to 2) Talonid is significantly wider than trigonid.

*Glasbius intricatus* Character 60 (from ? to 2) Talonid is significantly wider than trigonid.

*?Leptalestes cooki* Character 60 (from 2 to 1) Talonid and trigonid are of subequal width.

*Leptalestes krejcii* Character 60 (from 2 to 1) Talonid and tridonid are of subequal width.

*Leptalestes prokrejcii* Character 60 (from 2 to 1) Talonid and tridonid are of subequal width.

*Nortedelphys jasoni* Character 60 (from 2 to 1) Talonid and tridonid are of subequal width.

*Nortedelphys magnus* Character 60 (from 2 to 1) Talonid and tridonid are of subequal width.

*Protolambda clemensi* Character 60 (from 2 to 1) Talonid and tridonid are of subequal width.

*Peradectes minor* Character 81 (from 0 to 1) m3 cristid obliqua meets the distal trigonid wall buccal to the protocristid notch.

*Kielantherium*. Specimen PSS 10-16, a dentary fragment with four preserved cheek teeth preceded by alveoli for at least four double-rooted teeth is interpreted to represent p5-m3 preceeded by roots for p1-p4. Based on this interpretation, it follows that *Kielantherium* has five double-rooted premolars with a molariform p5. This agrees with the interpretation by Luo et al. ([Luo et al., 2002](#_ENREF_78)), but is at odds with the interpretation by others that Kielantherium possessed four molars ([Lopatin and Averianov, 2007](#_ENREF_75); [Luo et al., 2003](#_ENREF_76))

Character 85 changed and rescored:

m3 size: Ultimate molar is small relative to first molar (ultimate molar length/first molar length less than 0.8). (0); Ultimate molar is not small relative to first molar (ultimate molar L/first molar L ≥ 0.8 m, ≤ 1.5). (1); Ultimate molar is large relative to first molar (ultimate molar L/first molar L > 1.5). (2)

**Characters ordered: 8, 13, 16, 19, 23, 25, 26, 34, 41, 42, 58, 60, 62, 63, 69, 72, 80, 82, 83.**

**Taxa added to the Williamson et al. (2012) dataset:**

*Juramaia sinensis*

*Asioryctes nemegetensis*

*Ukhaatherium nessovi*

Outgroup taxon:

*Juramaia sinensis*

**List of taxa and characters used in the parsimony analysis**

**Abbreviations**. **AMNH**, American Museum of Natural History, New York, USA; **MNA**, Museum of Northern Arizona, Flagstaff, USA; **OMNH**, Oklahoma Museum of Natural History, Norman, USA; **RAM**, Raymond M. Alf Museum of Paleontology, Claremont, California, USA; SMP-SMU, Shuler Museum of Paleontology, Southern Methodist University, Dallas, Texas, USA; **UNM**, University of New Mexico, Albuquerque, USA.

**Taxa**

*Adelodelphys muizoni* ([Cifelli, 2004](#_ENREF_15)): Descriptions and illustrations from Cifelli ([2004](#_ENREF_15)).

*Aenigmadelphys archeri* Cifelli and Johanson, 1994: Descriptions and illustrations from Cifelli ([1990a](#_ENREF_9)) and Cifelli and Johanson ([1994](#_ENREF_19)). Cast of OMNH 20120, 20531, and 20160.

*Albertatherium primum* Fox, 1971: Descriptions and illustrations from Fox ([1971](#_ENREF_35)) and Johanson ([1995](#_ENREF_55)).

*Albertatherium secundum* Johanson, 1995: Descriptions and illustrations from Johanson ([1995](#_ENREF_55)).

*Alphadon attaragos* Lillegraven and McKenna, 1986: Descriptions and illustrations from Lillegraven and McKenna ([1986](#_ENREF_72)).

*Alphadon halleyi* Sahni, 1972: Descriptions and illustrations from Fox ([1979](#_ENREF_37)), Lillegraven and McKenna ([1986](#_ENREF_72)), Montellano ([1988](#_ENREF_89), [1992](#_ENREF_90)), and Sahni ([1972](#_ENREF_101)). Cast of AMNH 77367 (holotype), OMNH 20119, and 20171.

*Alphadon marshi* Simpson, 1927: Description and illustrations from Archibald ([1982](#_ENREF_2)), Clemens ([1966](#_ENREF_23)), Johanson ([1996b](#_ENREF_57)), Lillegraven ([1969](#_ENREF_71)), and Simpson ([1927](#_ENREF_104)). Cast of AMNH 58752, 58789, and 58830.

*Alphadon perexiguus* Cifelli, 1994: Descriptions and illustrations from Cifelli ([1994](#_ENREF_14)).

*Alphadon sahni* Lillegraven and McKenna, 1986: Descriptions and illustrations from Lillegraven and McKenna ([1986](#_ENREF_72)).

*Alphadon wilsoni* Lillegraven, 1969: Description and illustrations from Lillegraven ([1969](#_ENREF_71)).

*Anchistodelphys archibaldi* Cifelli, 1990b: Descriptions and illustrations from Cifelli ([1990b](#_ENREF_10)). Cast of MNA V4558, 4532, 4545, OMNH 20133, and 20968.

*Anchistodelphys delicatus* Cifelli, 1990a: Descriptions and illustrations from Cifelli ([1990c](#_ENREF_11)). Eaton and Cifelli ([Eaton and Cifelli, 2013](#_ENREF_34)) questioningly placed this species within *Varalphadon*.

*Apistodon exiguus* Fox, 1971: Descriptions and illustrations from Davis ([2007](#_ENREF_28)) and Fox ([1971](#_ENREF_35)).

*?Aquiladelphis laurae* Eaton, 2006: Descriptions and illustrations from Eaton ([2006](#_ENREF_32)).

*Aquiladelphis incus* Fox, 1971: Descriptions and illustrations from Davis ([2007](#_ENREF_28)) and Fox ([1971](#_ENREF_35)).

*Aquiladelphis minor* Fox, 1971: Descriptions and illustrations from Davis ([2007](#_ENREF_28)) and Fox ([1971](#_ENREF_35)). We follow Davis ([2007](#_ENREF_28)) in considering “*Pediomys*” *fassetti* ([Rigby and Wolberg, 1987](#_ENREF_96)) to be a junior subjective synonym.

*Armintodelphys blacki* Krishtalka and Stucky, 1983b: Descriptions and illustrations from Krishtalka and Stucky ([1983a](#_ENREF_66), [b](#_ENREF_67)).

*Armintodelphys dawsoni* Krishtalka and Stucky, 1983b: Descriptions and illustrations from Krishtalka and Stucky ([1983a](#_ENREF_66), [b](#_ENREF_67), [1984](#_ENREF_68)).

*Asiatherium reshetovi* Trofimov and Szalay, 1993: Descriptions and illustrations from Trofimov and Szalay ([1993](#_ENREF_114)) and Szalay and Trofimov ([1996](#_ENREF_113)).

*Asioryctes nemegetensis* Kielan-Jaworowska, 1975: Descriptions and illustrations from Kielan-Jaworowska ([Kielan-Jaworowska, 1975](#_ENREF_58)), Kielan-Jaworowska et al. ([Kielan-Jaworowska et al., 2004](#_ENREF_60)), Archibald and Averianov ([Archibald and Averianov, 2006](#_ENREF_1)), and Wible et al. ([Wible et al., 2009](#_ENREF_117)).

*Atokatheridium boreni* Kielan-Jaworowska and Cifelli, 2001: Descriptions and illustrations from Kielan-Jaworowska and Cifelli ([2001](#_ENREF_59)).

*Bistius bondi* Clemens and Lillegraven, 1986: Descriptions and illustrations from Clemens and Lillegraven ([1986](#_ENREF_24)).

*Copedelphys innominata* ([Simpson, 1928](#_ENREF_105)): Descriptions and illustrations from Krishtalka and Stucky ([1983a](#_ENREF_66), [b](#_ENREF_67)) and Simpson ([1928](#_ENREF_105)).

*Dakotadens morrowi* Eaton, 1993: Descriptions and illustrations from Eaton ([1993](#_ENREF_31)).

*Deltatheridium pretrituberculare* Gregory and Simpson, 1926: Descriptions and illustrations from Gregory and Simpson ([1926](#_ENREF_49)) and Rougier et al. ([1998](#_ENREF_98); [2004](#_ENREF_99)).

*Deltatheroides cretacicus* Gregory and Simpson, 1926: Descriptions and illustrations from Gregory and Simpson (1926) and Rougier et al. ([1998](#_ENREF_98); [2004](#_ENREF_99)).

*Didelphodon coyi* Fox and Naylor, 1986: Descriptions and illustrations from Fox and Naylor ([1986](#_ENREF_41), [2006](#_ENREF_42)).

*Didelphodon vorax* Marsh, 1889: Descriptions and illustrations from Clemens ([Clemens, 1968](#_ENREF_20), [1966](#_ENREF_23)), Fox and Naylor ([1986](#_ENREF_41), [2006](#_ENREF_42)), and Lofgren ([1992](#_ENREF_73)).

*Ectocentrocristus foxi* Rigby and Wolberg, 1987: Descriptions and illustrations from Case et al. ([2005](#_ENREF_8)), Fox ([1979](#_ENREF_37)), Rigby and Wolberg ([1987](#_ENREF_96)), and Sahni ([1972](#_ENREF_101)) and examinations of cast of AMNH 77372 (holotype) amd (AMNH 77371).

*Eoalphadon clemensi* (Eaton, 1993): Descriptions and illustrations from Eaton ([1993](#_ENREF_31), [2009](#_ENREF_33)).

*Eoalphadon lillegraveni* (Eaton, 1993): Descriptions and illustrations from Eaton ([1993](#_ENREF_31), [2009](#_ENREF_33)).

*Eoalphadon woodburnei* Eaton, 2009: Descriptions and illustrations from Eaton ([2009](#_ENREF_33)).

*Eodelphis browni* Matthew, 1916: Descriptions and illustrations from Fox ([1981](#_ENREF_38)), Fox and Naylor ([2006](#_ENREF_42)), and Matthew ([1916](#_ENREF_84)).

*Eodelphis cutleri* Woodard, 1916: Descriptions and illustrations from Fox ([1981](#_ENREF_38)), Fox and Naylor ([1986](#_ENREF_41), [2006](#_ENREF_42)), Russell ([1952](#_ENREF_100)), and Woodard ([1916](#_ENREF_108)).

*Glasbius intricatus* Clemens, 1966: Descriptions and illustrations from Clemens ([1979](#_ENREF_21); [1966](#_ENREF_23)).

*Glasbius twitchelli* Archibald, 1982: Descriptions and illustrations from Archibald ([1982](#_ENREF_2)).

*Golerdelphys stocki* Williamson and Lofgren ([Williamson and Lofgren, 2014](#_ENREF_119)), formerly referred to as the “Goler Formation taxon” by Williamson et al. (2012): Based on RAM 6432, a right M2, and RAM 6699, a right m2 or m3 ([Williamson and Lofgren, 2014](#_ENREF_119)).

*Hatcheritherium alpha* Case et al., 2005: Descriptions and illustrations from Case et al. ([2005](#_ENREF_8)).

*Herpetotherium comstocki* Cope, 1884: Descriptions and illustrations from Cope ([1884](#_ENREF_26)) and Krishtalka and Stucky ([1983a](#_ENREF_66)). Taxonomy follows Korth ([2008](#_ENREF_64)).

*Herpetotherium edwardi* Gazin, 1952: Descriptions and illustrations from Gazin ([1952](#_ENREF_44)) and Krishtalka and Stucky ([1983a](#_ENREF_66)). Taxonomy follows Korth ([2008](#_ENREF_64)).

*Herpetotherium fugax* ([Cope, 1873](#_ENREF_25)): Descriptions and illustrations from Fox ([1983](#_ENREF_39)), Green and Martin ([1976](#_ENREF_48)), and Korth ([1994](#_ENREF_63)). Taxonomy follows Korth ([2008](#_ENREF_64)).

*Herpetotherium knighti* McGrew, 1959: Descriptions and illustrations from Krishtalka and Stucky ([1983a](#_ENREF_66)) and McGrew ([1959](#_ENREF_87)). Taxonomy follows Korth ([2008](#_ENREF_64)).

*Herpetotherium marsupium* Troxell, 1923: Descriptions and illustrations from Krishtalka and Stucky ([1983a](#_ENREF_66)) and Troxell ([1923](#_ENREF_115)). Taxonomy follows Korth ([2008](#_ENREF_64)).

*Iqualadelphis lactea* Fox, 1987: Descriptions and illustrations from Davis ([2007](#_ENREF_28)) and Fox ([1987](#_ENREF_40)).

*Iugomortiferum thoringtoni* Cifelli, 1990b: Descriptions and illustrations from Cifelli ([1990b](#_ENREF_10)).

*Juramaia sinensis* Luo et al., 2011: Descriptions and illustrations from Luo et al. ([2011](#_ENREF_77)).

*Kokopellia juddi* Cifelli, 1993: Descriptions and illustrations from Cifelli ([1993a](#_ENREF_12)) and Cifelli and Muizon ([1997](#_ENREF_16)).

*?Leptalestes cooki* ([Clemens, 1966](#_ENREF_23)): Descriptions and illustrations from Davis ([2007](#_ENREF_28)). Cast of AMNH 58756, 58770.

*Leptalestes krejcii* (Clemens, 1966): Descriptions and illustrations from Clemens ([1966](#_ENREF_23)) and Davis ([2007](#_ENREF_28)). Cast of AMNH 58735 and UCMP 51390 (holotype).

*Leptalestes prokrejcii* (Fox, 1979): Descriptions and illustrations from Davis ([2007](#_ENREF_28)) and Fox ([1979](#_ENREF_37)). We follow Davis ([2007](#_ENREF_28)) in considering *Aquiladelphis paraminor* ([Rigby and Wolberg, 1987](#_ENREF_96)) to be a junior synonym.

*Leptalestes toevsi* Hunter et al., 2010: Descriptions and illustrations from Hunter et al. ([2010](#_ENREF_54)). This is equivalent to the “St Mary River Pediomyid” of Davis ([2007](#_ENREF_28)).

*Maastrichtidelphys meurismeti* Martin et al., 2005: Descriptions and illustrations from Martin et al. ([2005](#_ENREF_83)).

*Mimoperadectes houdei* Horovitz et al., 2009: Descriptions and illustrations from Horovitz et al. ([2009](#_ENREF_52)). *Mimoperadectes sowasheensis* Beard and Dawson, ([2009](#_ENREF_5)) is treated as a junior synonym.

*Mimoperadectes labrus* Bown and Rose, 1979: Descriptions and illustrations from Bown and Rose ([1979](#_ENREF_6)), Strait ([2001](#_ENREF_112)), Gingerich and Smith ([2006](#_ENREF_45)), and Horovitz et al. ([2009](#_ENREF_52)).

*Nanocuris improvida* Fox et al., 2007: Descriptions and illustrations from Fox et al. ([2007](#_ENREF_43)) and Wilson and Riedel ([2010](#_ENREF_121)). We consider AMNH 59451 and UALP 4085b to represent an M1 and M2, respectively, of *N. improvida*

*Nortedelphys jasoni* ([Storer, 1991](#_ENREF_111)): Description and illustrations from Case et al. ([2005](#_ENREF_8)), Johanson ([1996b](#_ENREF_57)), and Storer ([1991](#_ENREF_111)). Cast of UCMP 51385. As discussed in the text, *N. intermedium* Case et al., 2005 is considered a junior synonym of *N. jasoni*.

*Nortedelphys magnus* Case et al., 2005: Descriptions and illustrations from Case et al. (2005), Johanson ([1996b](#_ENREF_57)), and Lillegraven ([1969](#_ENREF_71)). Cast of UCMP 44095.

*Nortedelphys minimus* Case et al., 2005: Descriptions and illustrations from Case et al. ([2005](#_ENREF_8)).

*Oklatheridium szalayi* Davis et al., 2008: Descriptions and illustrations from Davis et al. ([2008](#_ENREF_29)).

*Pariadens kirklandi* Cifelli and Eaton, 1987: Descriptions and illustrations from Cifelli and Eaton ([1987](#_ENREF_18)) and Eaton ([1993](#_ENREF_31)).

*Pariadens mckennai* Cifelli, 2004: Descriptions and illustrations from Cifelli ([2004](#_ENREF_15)).

*Pediomys elegans* ([Marsh, 1889](#_ENREF_79)): Description and illustrations from Davis ([2007](#_ENREF_28)). Casts of AMNH 58768 and 58809. Scored for characters of “petrosal A” of Averianov et al. (2010).

*Peradectes californicus* ([Stock, 1936](#_ENREF_110)): Descriptions and illustrations from Krishtalka and Stucky ([1983a](#_ENREF_66)).

*Peradectes chesteri* ([Gazin, 1952](#_ENREF_44)): Descriptions and illustrations from Gazin ([1952](#_ENREF_44)) and Krishtalka and Stucky ([1983a](#_ENREF_66)).

*Peradectes elegans* Matthew and Granger, 1921: Descriptions and illustrations from Gazin (1956), Matthew and Granger ([1921](#_ENREF_86)), and Krishtalka and Stucky ([1983a](#_ENREF_66)). AMNH 17376 (holotype), 17383, 93665, and USNM 20979 (holotype of *P. pauli* Gazin, 1956). As discussed in the text, *Peradectes pauli* Gazin, 1956, is considered to be a junior synonym of *P. elegans*.

*Peradectes gulottai* Rose, 2010: Descriptions and illustrations from Rose ([2010](#_ENREF_97)).

*Peradectes* *coproxeches*. Williamson and Taylor, 2011. Based on specimens described and illustrated by Williamson and Taylor ([Williamson and Taylor, 2011](#_ENREF_120)).

*Peradectes* *minor* Clemens, 2006: Descriptions and illustrations from Clemens ([2006](#_ENREF_22)).

*Peradectes protinnominatus* McKenna, 1960: Descriptions and illustrations from Krishtalka and Stucky ([1983a](#_ENREF_66)) and McKenna ([1960](#_ENREF_88)). Casts of UCMP 44077 (holotype), 44767, and 47738. We did not include teeth from the late Paleocene (Tiffanian) of Saskatchewan, tentatively referred to *P. protinnominatus* by Rankin (2009), in this taxon.

*Prokennalestes trofimovi* Kielan-Jaworowska and Dashzeveg, 1989: Descriptions and illustrations from Kielan-Jaworowska and Dashzeveg ([1989](#_ENREF_61)) and Sigogneau-Russell et al. ([1992](#_ENREF_103)).

*Protalphadon foxi* Johanson, 1996: Descriptions and illustrations from Johanson ([1996b](#_ENREF_57)).

*Protalphadon lulli* ([Clemens, 1966](#_ENREF_23)): Descriptions and illustrations from Clemens ([Clemens, 1966](#_ENREF_23)) and Johanson ([1996b](#_ENREF_57)). Casts of AMNH 58758 and 58760.

*?Protolambda clemensi* Sahni, 1972: Descriptions and illustrations from Davis ([2007](#_ENREF_28)) and Sahni ([1972](#_ENREF_101)).

*Protolambda florencae* ([Clemens, 1966](#_ENREF_23)): Descriptions and illustrations from Clemens ([1966](#_ENREF_23)) and Davis ([2007](#_ENREF_28)).

*Protolambda hatcheri* Osborn, 1898: Descriptions and illustrations from Davis ([2007](#_ENREF_28)).

*Pucadelphys andinus* Marshall and Muizon, 1988: Descriptions and illustrations from Marshall and Muizon ([1988](#_ENREF_80); [1995](#_ENREF_81))

*Roberthoffstetteria nationalgeographica* Marshall et al., 1983. Descriptions and illustrations from Goin et al. ([2003](#_ENREF_46)) and Marshall et al. ([1983](#_ENREF_82)).

*Sinbadelphys schmidti* Cifelli, 2004: Descriptions and illustrations from Cifelli ([2004](#_ENREF_15)).

*Sulestes karakshi* Nessov, 1985: Descriptions and illustrations from Nessov ([1985](#_ENREF_92)), Kielan-Jaworowska and Nessov ([1990](#_ENREF_62)), Kielan-Jaworowska et al. ([2004](#_ENREF_60)), Davis et al. ([2008](#_ENREF_29)) and Averianov et al. (2010). We follow Averianov et al. (2010) in considering *Marsasia aenigma* Nessov, 1997 and *Deltatherus kizylkumensis* (Nessov, 1993) to be junior synonyms of *Sulestes karakshi*.

*Swaindelphys cifellii* Johanson, 1996a: Descriptions and illustrations from Johanson ([1996a](#_ENREF_56)).

*Swaindelphys* *encinensis*. Williamson and Taylor, 2011. Based on specimens described and illustrated by Williamson and Taylor ([2011](#_ENREF_120)).

*Swaindelphys* *johansoni* Williamson and Taylor 2011. Based on specimens described and illustrated by Williamson and Taylor ([Williamson and Taylor, 2011](#_ENREF_120)).

*Szalinia gracilis* Muizon and Cifelli, 2001: Descriptions and illustrations from Muizon and Cifelli ([2001](#_ENREF_30)).

*Thylacodon pusillus* Matthew and Granger, 1921. Descriptions and illustrations from Clemens ([2006](#_ENREF_22)), Matthew ([1937](#_ENREF_85)), and Matthew and Granger ([1921](#_ENREF_86)). Scoring for p3 based on UC 35070.

*Thylacodon* *montana* Williamson et al., 2012: Descriptions and illustrations from Archibald ([1982](#_ENREF_2)), Lofgren ([1995](#_ENREF_74)), and Clemens ([2006](#_ENREF_22)).

*Turgidodon lillegraveni* Cifelli, 1990a: Descriptions and illustrations from Cifelli ([1990a](#_ENREF_9)) and Johanson ([1996b](#_ENREF_57)).

*Turgidodon madseni* Cifelli, 1990a: Descriptions and illustrations from Cifelli ([1990a](#_ENREF_9)) and Johanson ([1996b](#_ENREF_57)).

*Turgidodon petiminis* Storer, 1991: Descriptions and illustrations from Johanson ([1996b](#_ENREF_57)) and Storer ([1991](#_ENREF_111)).

*Turgidodon praesagus* ([Russell, 1952](#_ENREF_100)): Descriptions and illustrations from Johanson ([1996b](#_ENREF_57)) and Russell ([1952](#_ENREF_100)). There is disagreement regarding specific identification of some specimens referred to the genus *Turgidodon* ([see Johanson, 1996b](#_ENREF_57)). We follow Cifelli ([1990a](#_ENREF_9)) and Johanson ([1996b](#_ENREF_57)) in regarding specimens referred to *T. praesagus* from the Judith River Formation by Sahni ([1972](#_ENREF_101)) to be *T. russelli*.

*Turgidodon rhaister* Clemens, 1966: Descriptions and illustrations from Clemens ([1966](#_ENREF_23)) and Johanson ([1996b](#_ENREF_57)). Scored for petrosal characters of “Turgidodon” in Averianov et al. (2010).

*Turgidodon russelli* (Fox, 1979): Descriptions and illustrations from Fox ([1979](#_ENREF_37)), Hunter et al. ([2010](#_ENREF_54)), Johanson ([1996b](#_ENREF_57)), Lillegraven and McKenna ([1986](#_ENREF_72)), and Montellano ([1992](#_ENREF_90)). Hunter et al. ([2010](#_ENREF_54)) considered *Turgidodon* ?*parapraesagus* ([Rigby and Wolberg, 1987](#_ENREF_96)) to be a synonym.

*Ukhaatherium nessovi* Novacek et al., 1997: Description and illustrations from Novacek et al. ([Novacek et al., 1997](#_ENREF_95)), Kielan-Jaworowska et al. ([Kielan-Jaworowska et al., 2004](#_ENREF_60)), Archibald and Averianov ([Archibald and Averianov, 2006](#_ENREF_1)), and Wible et al. ([Wible et al., 2009](#_ENREF_117)).

*Varalphadon creber* (Fox, 1971): Descriptions and illustrations from Fox ([1971](#_ENREF_35)) and Johanson ([1996b](#_ENREF_57)).

*Varalphadon crebreforme* (Cifelli, 1990c): Descriptions and illustrations from Cifelli ([1990b](#_ENREF_10)) and Johanson ([1996b](#_ENREF_57)).

*Varalphadon wahweapensis* (Cifelli, 1990b): Descriptions and illustrations from Cifelli ([1990b](#_ENREF_10)) and Johanson ([1996b](#_ENREF_57)). Casts of OMNH 20109, 20115, 20121, 20123, 20467, 20536, 20587, 20597, MNA V4516, and 4574.

**Metatherian taxa not included**

*Alphadon eatoni* Cifelli and Muizon, 1998: Known only from holotype, a partial dentary with lower teeth.

*Arcantiodelphys marchandi* Vullo et al. 2009: Known only from the holotype, a partial upper molar, and three partial lower teeth.

*Boreodon matutinus* Lambe, ([1902](#_ENREF_70)): Considered a nomen dubium by Fox and Naylor ([2006](#_ENREF_42)).

*Delphodon comptus* Simpson, 1927: Considered a nomen dubium following Fox and Naylor (2006).

*Didelphodon padanicus* Cope, 1892: Descriptions and illustrations from Cope ([1892](#_ENREF_27)) and Matthew ([1916](#_ENREF_84)). Considered a nomen nudum by Cifelli and de Muizon ([1998](#_ENREF_17)).

*Esteslestes ensis* Novacek et al., 1991: Descriptions and illustrations from Novacek et al. ([1991](#_ENREF_94)). Known only from a partial dentary with p3, m3-4.

*Holoclemensia texana* ([Slaughter, 1968](#_ENREF_107)): Based on Descriptions and illustrations from Slaughter ([Slaughter, 1968](#_ENREF_107)) and Jacobs et al. (1989), based on the holotype, a partial maxilla with a partial penultimate upper molar (SMP-SMU 61997) and the paratype ultimate upper molar (SMP-SMU 62009) and a referred lower molar (SMP-SMU 62131). Casts of SMP-SMU 61997, 62009, and 62131. Upper premolar characters are based on referral of P4 (SMP-SMU 61948) and P5 (CNHM PM 931) following Averianov et al. (2010).

*Oxlestes grandis* ([Nessov, 1982](#_ENREF_91)) and *Khuduklestes bohlini* ([Nessov et al., 1994](#_ENREF_93)) are each based on a partial axis vertebra. Both were tentatively regarded as deltatheroidan metatherians by ([Kielan-Jaworowska et al., 2004](#_ENREF_60)), but Averianov and Archibald ([2005](#_ENREF_3)) suggested that they might represent “zhelestid” eutherians.

*Pappotherium pattersoni* Slaughter ([1965](#_ENREF_106)): Descriptions and illustrations from Butler ([1978](#_ENREF_7)), Fox ([Fox, 1975](#_ENREF_36)), Jacobs et al. (1989), and Kielan-Jaworowska et al. ([2004](#_ENREF_60)). Includes lower molars tentatively referred to this taxon by Butler ([1978](#_ENREF_7)). Cast SMP-SMU 61725 (holotype).

*Sinodelphys szalayi* Luo et al., 2003: Descriptions and illustrations from Luo et al. (2003). This specimen includes a poorly preserved or poorly exposed dentition.

*Turgidodon parapraesagus* ([Rigby and Wolberg, 1987](#_ENREF_96)): The holotype of *T. parapraesagus*, UNM-B5338, a right dentary fragment with m2-4, is now lost. Considered a nomen dubium by Johanson ([1996b](#_ENREF_57)) and was considered a synonym of *Turgidodon russelli* by Cifelli ([1990a](#_ENREF_9)).

Krause ([Krause, 2001](#_ENREF_65)) reported a metatherian from the Late Cretaceous of Madagascar based on a partial lower molar. Averianov et al. (2003) argued that this tooth represented a “zhelestid” eutherian.

Several phylogenetic analyses have included an unnamed taxon based on a skull, the “Gurlin Tsav Skull” from the Maastrichtian of Mongolia (e.g., [Averianov et al., 2010](#_ENREF_4); [Rougier et al., 1998](#_ENREF_98); [Rougier et al., 2004](#_ENREF_99); [Szalay and Trofimov, 1996](#_ENREF_113)). We have not examined this specimen and have not included it in our analysis.

**Characters**

1. Lower incisor number: No more than 3. (0); 4 or more. (1). Modified from Horovitz and Sánchez-Villagra ([2003](#_ENREF_53)), character 151; Horovitz et al. ([2009](#_ENREF_52)), character 148; Rougier et al. ([1998](#_ENREF_98); [2004](#_ENREF_99)), character 42.

2. Lower incisor staggering: Not staggered. (0); Staggered (medially staggered position of root and alveolus of i2; i2 larger than i1, i3, or i4). (1). Condition described in Cifelli and Muizon ([1997](#_ENREF_16); [1998](#_ENREF_17)), Hershkovitz ([1982](#_ENREF_50); [1995](#_ENREF_51)) and Marshall and Muizon ([1995, p. 68](#_ENREF_81)), but with the i2 being staggered rather than the i3 as originally described by Hershkovitz ([1982](#_ENREF_50)) as was proposed by Sánchez-Villagra et al. ([2007](#_ENREF_102)) and followed by Horovitz et al. ([2009, Character 169](#_ENREF_52)); Rougier et al. ([1998](#_ENREF_98); [2004](#_ENREF_99)), character 43.

3. C: Double rooted. (0); Single rooted. (1). From Horovitz and Sánchez-Villagra ([2003](#_ENREF_53)), character 171; Horovitz et al. ([2009](#_ENREF_52)), character 168.

4. Number of lower premolars: Five. (0); Four or less. (1). Modified from Kielan-Jaworowska et al. ([2004](#_ENREF_60)), character 108; modified from Rougier et al. ([1998](#_ENREF_98); [2004](#_ENREF_99)), character 1.5. First upper premolar: Single rooted. (0); Double rooted (1).

6. First lower premolar: Oriented in line with jaw axis. (0); Oblique. (1). From Averianov et al. (2010), character 43.

7. Deciduous precursors for ante-P4/p4 dentition: Present (0); Absent (1).

8. DP4 mesial stylar shelf: Narrow, but present. (0); Present as an ectocingulum (1); Absent. (2). Ordered.

9. DP4 parastylar lobe: Moderate in size and stylar cusp A is in line with paracone and metacone. (0); Expanded and stylar cusp A is positioned lingual to a line drawn from the paracone and metacone. (1).

10, DP4 stylar cusp B: Present (0); Absent (1).

11. DP4 stylar cusp C: Present. (0); Absent. (1).

12. DP4 stylar cusp C size: Smaller than stylar cusp D. (0); Larger than stylar cusp D. (1).

13. P4: Absent. (0); Small lingual bulge. (1); With an enlarged basin. (2). From Rougier et al. ([1998](#_ENREF_98); [2004](#_ENREF_99)), character 12. Ordered.

14. Number of roots on P4: Two. (0); Three. (1). From Rougier et al. ([1998](#_ENREF_98); [2004](#_ENREF_99)), character 13.

15. P5/p5 (permanent replacement tooth): present, (0); absent, deciduous tooth retained (1).

16. Inflation of P4/p4: Uninflated (main cusp of p4 is narrower than the trigonid of m1). (0); Somewhat inflated (the width of the main cusp of p4 is subequal to that of m1). (1); Markedly inflated (p4 is wider and longer than m1; P4 buccolingual width approaches that of M1). Lingual lobe or "accessory lobe" present. (2). Modified from Davis ([2007](#_ENREF_28)), character 56. Ordered.

17. Morphological features on the buccal cingulum or stylar shelf of the upper molars (excluding the parastyle and metastyle): Distinctive cingulum, without cuspules. (0); Individualized or even hypertrophied cuspules (1) (modified from Luo et al., 2011, character 99).

18. Stylar cusp A (dP5-M2): Absent. (0); Present. (1).

19. Stylar cusp D: Absent. (0); Variably present. (1); Consistently present. (2). From Davis ([2007](#_ENREF_28)), character 10. Ordered.

20. Stylar cusps B, C, and D: Stylar cusp D present, C and B absent. (0); Stylar cusp D present, C and B present. (1); Stylar cusp D and C present, B absent. (2); Stylar cusp D and B present, C absent. (3). Modified after Johanson ([1996b](#_ENREF_57)), character 6; Davis ([2007](#_ENREF_28)), character 6 and 8; Rougier et al. ([1998](#_ENREF_98); [2004](#_ENREF_99)), character 23; Vullo et al. ([2009](#_ENREF_116)), character 6.

21. Stylar cusp A (parastyle) position relative to stylar cusp B (stylocone): Lower than cusp B. (0); Closely approximated to cusp B. (1); Modified after Johanson ([1996b](#_ENREF_57)), character 2.

22. Stylar cusp A size relative to stylar cusp B (penultimate molar): Equal to or larger than stylar cusp B. (0); Smaller than stylar cusp B. (1). From Rougier et al. ([1998](#_ENREF_98); [2004](#_ENREF_99)), character 20.

23. Stylar cusp B (stylocone) size: Small (less than half the size of the paracone). (0); Moderately developed (greater than about half the size of paracone, but smaller than paracone). (1); Large (subequal to paracone). (2). Modified after Johanson ([1996b](#_ENREF_57)), character 3; Rougier et al. ([1998](#_ENREF_98); [2004](#_ENREF_99)), character 22; Davis ([2007](#_ENREF_28)), character 3. Ordered.

24. Position of stylar cusp B (stylocone) relative to paracone: Positioned nearly directly buccal to paracone. (0); Positioned mesiobuccal to paracone. (1). From Davis ([2007](#_ENREF_28)), character 5.

25. Stylar cusp C size: Small (present as a distinct cusp, but is equal to or smaller than stylar cusp A). (0); Well-developed (greater than stylar cusp A, but less than stylar cusp D). (1); Large (equal to or greater than stylar cusp B or D. (2). Modified after Davis ([2007](#_ENREF_28)), character 8, Vullo et al. ([2009](#_ENREF_116)), character 6. Ordered.

26. Presence of a "twinned" cusp anterior to cusp C: Absent. (0); Variably present. (1); Consistently present. (2). Ordered.

27. Position of cusp C relative to the ectoflexus (dP5-m2): Positioned at, or mesial to, the deepest part of the ectoflexus. (0); Positioned distal to the deepest part of the ectoflexus. (1). Modified after Johanson ([1996b](#_ENREF_57)), character 7 and Davis ([2007](#_ENREF_28)), character 9.

28. Position of cusp C relative to the buccal edge of the stylar shelf (dP5-M2): Positioned along the buccal edge of the stylar shelf. (0); Positioned lingual to the buccal edge of the stylar shelf. (1). Modified after Johanson ([1996b](#_ENREF_57)), character 8.

29. Relative size of stylar cusps C and D (M2): Stylar cusp C is smaller than D. (0); Stylar cusp C is equal to or larger than D. (1). Modified after Johanson ([1996b](#_ENREF_57)), character 10.

30. Cusp C shape in buccal view: Conical. (0); Squared at its apex. (1). Modified after Johanson ([1996b](#_ENREF_57)), character 11.

31. Stylar cusp D size (M2): Moderately developed. (0); Small. (1). Modified from Davis ([2007](#_ENREF_28)), character 11, Vullo et al. ([2009](#_ENREF_116)), character 7.

32. Position of Stylar cusp D relative to metacone: Positioned buccal to metacone. (0); Positioned mesiobuccal to metacone and closer to deepest part of ectoflexus. (1). From Davis ([2007](#_ENREF_28)), character 12.

33. Stylar cusp D shape at base: Broad and mesiodistally long at base. (0); Conical at base (1). From Davis (2007), character 14.

34. Mesial portion of stylar shelf (parastylar lobe) (M2): Not reduced. (0); Somewhat reduced so that it is buccolingually narrower than metastylar lobe. (1); Greatly reduced so that just a rim remains buccal to paracone and stylar cusp A is nearly directly mesial to paracone. (2). From Davis ([2007](#_ENREF_28)), characters 1 and 15 which are here combined. Ordered.

35. Preparacrista strength: Well-developed. (0); Weakly-developed or absent. (1). From Davis ([2007](#_ENREF_28)), character 16.

36. Preparacrista orientation: Preparacrista runs to a position below apex of cusp B (lingual side of cusp B) or to the mesiolingual face of cusp B. (0); Preparacrista runs to position mesial to the apex of cusp B or toward stylar cusp A if stylar cusp B is absent. (1). Modified from Davis ([2007](#_ENREF_28)), character 17.

37. Presence of carnassial notch along preparacrista: Present. (0); Absent. (1). From Davis ([2007](#_ENREF_28)), character 18.

38. Presence of carnassial notch along postmetacrista: Present. (0); Absent. (1). From Davis ([2007](#_ENREF_28)), character 20.

39. Ectoflexus Depth (M2): Deep (embayment equal to or greater than 10% of total width of tooth). (0); Shallow (embayment less than 10% of total width of tooth). (1). From Davis ([2007](#_ENREF_28)), character 21.

40. Ectoflexus across molar series (dP5-M2): Increases in depth distally through the molar series. (0); Little or no change in depth of ectoflexus along the molar series. (1). Modified after Johanson ([1996b](#_ENREF_57)), character 15; Davis ([2007](#_ENREF_28)), character 22.

41. Relative height of the paracone and metacone (dP5-M2). Paracone is taller than the metacone. (0); Paracone and metacone are subequal in height. (1); Metacone is taller than the paracone. (2). Modified from Johanson ([1996b](#_ENREF_57)), character 16; Davis ([2007](#_ENREF_28)), character 23. Approximately equivalent to Horovitz and Sánchez-Villagra ([2003](#_ENREF_53)), character 155; Horovitz et al. ([2009](#_ENREF_52)), character 152; Vullo et al. ([2009](#_ENREF_116)), character 10; Rougier et al. ([1998](#_ENREF_98); [2004](#_ENREF_99)), character 27. Ordered.

42. Relative size of paracone and metacone in buccal view (as measured mesiodistally; dP5-M2): Paracone is longer than the metacone. (0); Paracone and metacone are of equal size. (1); Metacone is longer than the paracone. (2). Modified from Johanson ([1996b](#_ENREF_57)), character 17; Davis ([2007](#_ENREF_28)), character 24. Approximately equivalent to Horovitz and Sánchez-Villagra ([2003](#_ENREF_53)), character 155; Horovitz et al. ([2009](#_ENREF_52)), character 152; and Vullo et al. ([2009](#_ENREF_116)), character 10; Rougier et al. ([1998](#_ENREF_98); [2004](#_ENREF_99)), character 27. Ordered.

43. Paracone and metacone shape (dP5-M2): Uninflated and unrounded. (0); The paracone is inflated and rounded compared to the metacone. (1); Both the paracone and metacone are inflated and rounded. (2). Modified from Davis ([2007](#_ENREF_28)), character 25.

44. The shape of the buccal faces of the paracone and metacone (dP5-M2): Flat or concave. (0); Buccal face of paracone is convex (rounded); buccal face of metacone is flat. (1); Convex or rounded. (2). Modified from Davis ([2007](#_ENREF_28)), character 26; Rougier et al. ([1998](#_ENREF_98); [2004](#_ENREF_99)), character 29.

45. The paracone and metacone relative separation at base (dP5-M2): Share a portion of their bases. (0); Entirely separate at their bases. (1). From Davis ([2007](#_ENREF_28)), character 27; Vullo et al. ([2009](#_ENREF_116)), character 11; Rougier et al. ([1998](#_ENREF_98); [2004](#_ENREF_99)), character 30.

46. Centrocrista morphology (refers to location of the deepest point of the centrocrista relative to an imaginary line between the apices of the paracone and metacone): Straight (equivalent to a U-shaped ectoloph). (0); Deflected buccally so that it is V-shaped (equivalent to a W-shaped ectoloph). (1); Invades stylar shelf as disconnected crests. (2). Modified from Johanson ([1996b](#_ENREF_57)) character 23; Horovitz and Sánchez-Villagra ([2003](#_ENREF_53)), character 156; Horovitz et al. ([2009](#_ENREF_52)) and Davis ([2007](#_ENREF_28)), character 29, Kielen-Jaworowska et al. ([2004](#_ENREF_60)), character 82, and after Case et al. ([2005](#_ENREF_8)); Rougier et al. ([1998](#_ENREF_98); [2004](#_ENREF_99)), character 31.

47. Paraconule and metaconule size: Small and weakly-developed (dP5-M2). (0); Large and strongly-developed. (1). Modified from Johanson ([1996b](#_ENREF_57)), character 24; Davis ([2007](#_ENREF_28)), character 30; and Vullo et al. ([2009](#_ENREF_116)), character 8.

48. Position of paraconule relative to protocone and paracone: Relatively closer to protocone or midway between protocone and paracone. (0); Relatively closer to paracone. (1). Modified from Davis ([2007](#_ENREF_28)), character 31.

49. Strength and morphology of the internal cristae: Well-developed. (0); Weakly-developed or absent. (1). From Davis ([2007](#_ENREF_28)), character 32; modified from Rougier et al. ([1998](#_ENREF_98); [2004](#_ENREF_99)), character 35.

50. Protocone height relative to paracone/metacone (whichever is taller): Shorter than paracone/metacone. (0); Approaching height of paracone/metacone (over half the height). (1). Modified from Davis ([2007](#_ENREF_28)), character 33; from Rougier et al. ([1998](#_ENREF_98); [2004](#_ENREF_99)), character 38.

51. Protocone position: Positioned mesiodistally midway between paracone and metacone. (0); Positioned mesial to the midway point between paracone and metacone. (1).

52. Protocone procumbency: Absent. (0); Present (1). From Rougier et al. (1998; 2004), caracter 37.

53. Protocone basal distal expansion (M1-2): Unexpanded. Protocone is V-shaped. (0); Expanded so that part of the protocone is somewhat lobe-shaped or "squared off." (1). Modified from Davis ([2007](#_ENREF_28)), character 35.

5. Pre- and postcingula: Absent. (0); Precingulum present, postcingulum absent. (1); Pre- and postcingula present. (2). Modified from Johanson ([1996b](#_ENREF_57)) character 28; Davis ([2007](#_ENREF_28)), character 36; and Vullo et al. ([2009](#_ENREF_116)), character 15.

55. Placement of paraconule, protocone, and metaconule: Not aligned in a row. (0); Aligned in a row. (1). From discussion by Goin et al. ([2003](#_ENREF_46)) and Case et al. ([2005](#_ENREF_8)).

56. Preprotocrista: Terminates lingual of base of paracone. (0); Joins preparaconular crista and extends buccally past base of paracone (presence of double rank prevallum/postvallid shearing). (1). From Cifelli ([1993b](#_ENREF_13)), Rougier et al. ([1998](#_ENREF_98); [2004](#_ENREF_99)), character 33.

57. Postprotocrista: Absent. (0); Present. (1).

58. Postprotocrista length (dP5-M2): Extends from protocone or merges with postmetaconule crista only to base of metacone. (0); Extends buccally to wrap around the distal side of metacone, but does not extend to distobuccal corner of tooth (all molars but last). (1); Extends beyond buccal base of metacone to near buccal margin of tooth. (2). Modified from Cifelli ([1993b](#_ENREF_13)), Davis (2007), character 39 and 41, Rougier et al. ([1998](#_ENREF_98); [2004](#_ENREF_99)), and Vullo et al. ([2009](#_ENREF_116)), character 9. Ordered.

59. Postmetacrista orientation (M2): Wide Line drawn between paracone and metacone forms approximately a right angle with postmetacrista. (0); Line drawn between paracone and metacone forms an obtuse angle (greater than about 100 degrees) with postmetacrista. (1).

60. Relative transverse widths of the trigonid and talonid (M1-M2): Trigonid is wider than talonid. (0); Talonid and trigonid are of subequal width. (1); Talonid is significantly wider than trigonid. (2). Modifed from Johanson ([1996b](#_ENREF_57)), character 32; Davis ([2007](#_ENREF_28)), character 42; Horovitz and Sánchez-Villagra ([2003](#_ENREF_53)), character 158; and Horivitz et al. ([2009](#_ENREF_52)), character 155; Rougier et al. ([1998](#_ENREF_98); [2004](#_ENREF_99)), character 50; and Vullo et al. ([2009](#_ENREF_116)), character 30. Ordered.

61. Relative lengths of trigonid and talonid (dP5-M2): long (trigonid length/talonid length is 1.8 or less). (0); Talonid short (trigonid length/talonid length is greater than 1.8). (1).

62. Relative mesiodistal lengths of paraconid and metaconids in lingual view (dp5-2): The paraconid is longer than the metaconid. (0); The two cusps are relatively equal in length. (1); The metaconid is longer than paraconid. (2). From Davis ([2007](#_ENREF_28)), character 45. Ordered.

63. Relative heights of paraconid and metaconid: Paraconid is taller than metaconid. (0); Two cusps are subequal in height. (1); Metaconid is taller than paraconid. (2). From Davis ([2007](#_ENREF_28)), character 44 and modified from Vullo et al. ([2009](#_ENREF_116)), character 22; Rougier et al. ([1998](#_ENREF_98); [2004](#_ENREF_99)), character 59. Ordered.

64. Mesiolingual face of the paraconid: Not strongly keeled. (0); Strongly keeled, sometimes with basal cusp. (1).

65. Position of paraconid relative to metaconid: Posititioned bucally relative to metaconid. (0); Positioned more lingually, such that the paraconid, metaconid and entoconid, if all present, line up mesiodistally. (1). From Davis ([2007](#_ENREF_28)), character 46 and modified from Vullo et al. ([2009](#_ENREF_116)), character 22.

66. Position of paraconid relative to metaconid (m1-3): Paraconid projects mesially. (0); Paraconid appressed to the metaconid. (1). Modified from Kielan-Jaworowska ([2004](#_ENREF_60)) and discussed by Fox and Naylor ([2006, p. 34](#_ENREF_42)). This also essentially includes Kielan-Jaworowska et al. (2004), character 72 (angle between the paracristid and protocristid), because, with the paraconid closely appressed to the metaconid, the angle between the paracristid and the protocristid is low.

67. Angle of trigonid (measured from entoconid to protoconid, with metaconid as vertex; all molars but first): Obtuse (greater than 95 degrees). (0); Approaches 90 degrees (equal to or less than 95 degrees). (1). From Davis ([2007](#_ENREF_28)), character 48.

68. Molar talonid: Narrow, lacking entoconid. (0); With at least three cusps, including entoconid (1).

69. Size of entoconid relative to hypoconulid (dp5-m2): Smaller than hypoconulid. (0); Comparable in size to hypoconulid. (1); Larger than hypoconulid. (2). From Davis ([2007](#_ENREF_28)), character 49 and modified from Vullo et al. ([2009](#_ENREF_116)), character 32. Ordered.

70. Height of entoconid relative to metaconid (m1-m2): Less than or equal to 0.30 height of metaconid. (0); Greater than 0.30 height of metaconid. (1). Modified From Davis ([2007](#_ENREF_28)), character 49; Rougier et al. ([1998](#_ENREF_98); [2004](#_ENREF_99)), character 54.

71. Shape of entoconid (dp5-m2): Buccolingually compressed and blade-like. (0); Conical. (1). Springer et al. ([1997](#_ENREF_109)), character 20 and Ladevèze and Muizon ([2007](#_ENREF_69)), character 65.

72. Position of hypoconulid relative to entoconid (dp5-m2): Hypoconulid and entoconid are not twinned or twinning is weak, hypoconulid is near tooth midline. (0); Hypoconulid and entoconid are strongly twinned, but hypoconulid is buccal to a position that is distal to entoconid. (1); Hypoconulid and entoconid are twinned and hypoconulid is positioned on the lingual margin of tooth, distal to entoconid. (2). Modified from Davis ([2007](#_ENREF_28)), character 50 and Vullo et al. ([2009](#_ENREF_116)), character 32; Rougier et al. ([1998](#_ENREF_98); [2004](#_ENREF_99)), character 52. Ordered.

73. Postcingulid (dp5-m2): Absent. (0); Present. (1).

74. Postcingulid (m3): Absent. (0); Present. (1).

75. Accessory cusp adjacent to entoconid along entocristid (entoconulid; dp5-m2): Absent. (0); Present. (1). From Davis ([2007](#_ENREF_28)), character 51.

76. m3 entoconid: Same relative size as in preceding molars. (0); Reduced compared to preceding molars. (1).

77. Presence of talonid ectocingulid (as extension of postcingulid): Absent. (0); Present. (1). From Davis ([2007](#_ENREF_28)), character 52.

78. Ventral extent of talonid portion of crown (in buccal view): Extends ventrally to level of trigonid or slightly past level of trigonid (ventral margin of crown is horizontal or slopes gently distally). (0); Significantly expanded such that the ventral margin of crown slopes steeply distally. (1). Modified from Davis ([2007](#_ENREF_28)), character 53.

79. Cristid obliqua (dp5-m2): Incomplete; postmetacristid present. (0); Complete. (1). Modified from Rougier et al. ([1998](#_ENREF_98); [2004](#_ENREF_99)), character 51.

80. Cristid obliqua (dp5-m2): Meets the distal trigonid wall at a point lingual or ventral to the protocristid notch. (0); Meets the distal trigonid wall buccal to the protocristid notch, approximately below apex of protoconid. (1); Meets the distal trigonid wall buccal to the protocristid notch near buccal margin of tooth. (2). Ordered. Modified from Davis (2007), characters 53 and 55; Horovitz and Sánchez-Villagra ([2003](#_ENREF_53)), character 160; Horovitz et al. ([2009](#_ENREF_52)), character 157; Johanson ([1996b](#_ENREF_57)), character 35; and Vullo et al. ([2009](#_ENREF_116)), character 27; Rougier et al. ([1998](#_ENREF_98); [2004](#_ENREF_99)), character 51. Ordered.

81. m3 cristid obliqua: Meets the distal trigonid wall at a point lingual or ventral to the protocristid notch. (0); Meets the distal trigonid wall buccal to the protocristid notch. (1).

82. Estimated mass (ln g) based on tooth measurements ([after Gordon, 2003](#_ENREF_47)): Small (equal to or less than 4). (0); Medium (greater than 4, equal to or less than 6). (1); Large (greater than 6). (2). Modified from Davis (2007), character 57. Ordered.

83. m3 molar size: m3 is small relative to dp5 (m3 Length/dp5 Length less than 0.8. (0); m3 is not small or large relative to dp5 (m3L/dp5 L equal to or greater than 0.8, less than 1.5). (1); m3 is large relative to dp5 (m3 L/dp5 L greater than 1.5). (2). Modified from Rougier et al. ([1998](#_ENREF_98); [2004](#_ENREF_99)), character 61. Ordered.

**References**

Archibald, D., and A. Averianov. 2006. Late Cretaceous asioryctitherian eutherian mammals from Uzbekistan and phylogenetic analysis of Asioryctitheria. Acta Palaeontologica Polonica 51:351-376.

Archibald, J. D. 1982. A study of Mammalia and geology across the Cretaceous-Tertiary boundary in Garfield County, Montana. University of California Publications in Geological Sciences 122:1-286.

Averianov, A., and D. J. Archibald. 2005. Mammals from the mid-Cretaceous Khodzhakul Formation, Kyzylkum Desert, Uzbekistan. Cretaceous Research 26:593-608.

Averianov, A. O., J. D. Archibald, and E. G. Ekdale. 2010. New material of the Late Cretaceous deltatheroidan mammal Sulestes from Uzbekistan and phylogenetic reassessment of the metatherian-eutherian dichotomy. Journal of Systematic Palaeontology 8:301-330.

Beard, K. C., and M. R. Dawson. 2009. Early Wasatchian mammals of the Red Hot local fauna, uppermost Tuscahoma Formation, Lauderdale County, Mississippi. Annals of Carnegie Museum 78:193-243.

Bown, T. M., and K. D. Rose. 1979. *Mimoperadectes*, a new marsupial, and *Worlandia*, a new dermopteran, from the lower part of the Willwood Formation (Early Eocene), Bighorn Basin, Wyoming. Contributions from the Museum of Paleontology, University of Michigan 25:89-104.

Butler, P. M. 1978. A new interpretation of the mammalian teeth of tribosphenic pattern from the Albian of Texas. Breviora 446:1-27.

Case, J. A., F. J. Goin, and M. O. Woodburne. 2005. "South American" marsupials from the Late Cretaceous of North America and the origin of marsupial cohorts. Journal of Mammalian Evolution 11:223-255.

Cifelli, R. L. 1990a. Cretaceous mammals of southern Utah; I, Marsupials from the Kaiparowits Formation (Judithian). Journal of Vertebrate Paleontology 10:295-319.

Cifelli, R. L. 1990b. Cretaceous mammals of southern Utah; II, Marsupials and marsupial-like mammals from the Wahweap Formation (early Campanian). Journal of Vertebrate Paleontology 10:320-321.

Cifelli, R. L. 1990c. Cretaceous mammals of southern Utah; III, Therian mammals from the Turonian (early Late Cretaceous). Journal of Vertebrate Paleontology 10:332-345.

Cifelli, R. L. 1993a. Early Cretaceous mammal from North America and the evolution of marsupial dental characters. Proceedings of the National Academy of Sciences (USA) 90:9413-9416.

Cifelli, R. L. 1993b. Theria of metatherian-eutherian grade and the origin of marsupials; pp. 205-215 *i*n F. S. Szalay, M. J. Novacek, and M. C. McKenna (eds.), Mammal Phylogeny: Placentals. Springer-Verlag, New York.

Cifelli, R. L. 1994. Therian mammals of the Terlingua local fauna (Judithian), Aguja Formation, Big Bend of the Rio Grande, Texas. Contributions to Geology 30:117-136.

Cifelli, R. L. 2004. Marsupial mammals from the Albian-Cenomanian (Early-Late Cretaceous) boundary, Utah. Bulletin of the American Museum of Natural History 285:62-79.

Cifelli, R. L., and C. de Muizon. 1997. Dentition and jaw of *Kokopellia juddi*, a primitive marsupial or near-marsupial from the medial Cretaceous. Journal of Mammalian Evolution 4:241-258.

Cifelli, R. L., and C. de Muizon. 1998. Marsupial mammal from the Upper Cretaceous North Horn Formation, central Utah. Journal of Paleontology 72:532-537.

Cifelli, R. L., and J. G. Eaton. 1987. Marsupial from the earliest Late Cretaceous of Western US. Nature 325:520-522.

Cifelli, R. L., and Z. Johanson. 1994. New marsupial from the Upper Cretaceous of Utah. Journal of Vertebrate Paleontology 14:292-295.

Clemens, W. A. 1968. A Mandible of *Didelphodon vorax* (Marsupialia, Mammalia). Los Angeles County Museum Contributions in Science:1-11.

Clemens, W. A. 1979. Marsupialia; pp. 192-220 *i*n J. A. Lillegraven, Z. Kielan-Jaworowska, and W. A. Clemens (eds.), Mesozoic Mammals, the First Two-thirds of Mammalian History. University of California Press, Berkeley.

Clemens, W. A. 2006. Early Paleocene (Puercan) peradectid marsupials from northeastern Montana, North American Western Interior. Palaeontographica Abteilung A 27:19-31.

Clemens, W. A., Jr. 1966. Fossil mammals of the type Lance Formation, Wyoming; Part II, Marsupialia. University of California Publications in Geological Sciences 62.

Clemens, W. A., and J. A. Lillegraven. 1986. New Late Cretaceous, North American advanced therian mammals that fit neither the marsupial nor eutherian molds. Contributions to Geology. Special Paper 3:55-85.

Cope, E. D. 1873. Third notice o fthe extinct Vertebrata from the Tertiary of the Plains. Paleontology Bulletin 16:1-8.

Cope, E. D. 1884. The Tertiary Marsupialia. American Naturalist 18:686-697.

Cope, E. D. 1892. Geology and Paleontology. The American Naturalist 26:754-765.

Davis, B. M. 2007. A revision of "pediomyid" marsupials from the Late Cretaceous of North America. Acta Palaeontologica Polonica 52:217-256.

Davis, B. M., R. L. Cifelli, and Z. Kielan-Jaworowska. 2008. Earliest evidence of Deltatheroida (Mammalia: Metatheria) from the Early Cretaceous of North America; pp. 3-24 *i*n E. J. Sargis, and M. Dagosto (eds.), Mammalian Evolutionary Morphology: A Tribute to Frederick S. Szalay. Springer, Dordecht.

de Muizon, C., and R. L. Cifelli. 2001. A new basal "didelphoid" (Marsupialia, Mammalia) from the early Paleocene of Tiupampa (Bolivia). Journal of Vertebrate Paleontology 21:87-97.

Eaton, J. G. 1993. Therian mammals from the Cenomanian (Upper Cretaceous) Dakota Formation, southwestern Utah. Journal of Paleontology 13:105-124.

Eaton, J. G. 2006. Late Cretaceous mammals from Cedar Canyon, southwestern Utah. New Mexico Museum of Natural History and Science Bulletin 35:373-402.

Eaton, J. G. 2009. Cenomanian (Late Cretaceous) mammals from Cedar Canyon, southwestern Utah, and a revision of Cenomanian *Alphadon*-like marsupials; pp. 97-110 *i*n L. B. albright, III (ed.), Papers on Geology. Vertebrate Paleontology and Biostratigraphy in Honor of Michael O. Woodburne. Museum of Northern Arizona, Flagstaff.

Eaton, J. G., and R. L. Cifelli. 2013. Review of Late Cretaceous mammalian faunas of the Kaiparowits and Paunsaugunt plateaus, southwestern, Utah; pp. 319-369 *i*n A. L. Titus, and M. A. Loewen (eds.), At the Top of the Grand Staircase: The Late Cretaceous of Southern Utah. Indiana University Press, Bloomington.

Fox, R. C. ed (1971) Marsupial mammals from the early Campanian Milk River Formation, Alberta, Canada.

Fox, R. C. 1975. Molar structure and function in the Early Cretaceous mammal *Pappotherium*: Evolutionary implications for Mesozoic Theria. Canadian Journal of Earth Science 12:412-442.

Fox, R. C. 1979. Mammals of the Upper Cretaceous Oldman Formation, Alberta. I. *Alphadon* Simpson (Marsupialia). Canadian Journal of Earth Sciences 16:91-102.

Fox, R. C. 1981. Mammals from the Upper Cretaceous Oldman Formation, Alberta. V. *Eodelphis* Matthew, and the evolution of the Stagodontidae (Marsupialia). Canadian Journal of Earth Sciences 18:350-365.

Fox, R. C. 1983. Notes on the North American marsupials *Herpetotherium* and *Peradectes*. Canadian Journal of Earth Sciences 20:1565-1578.

Fox, R. C. 1987. An ancestral marsupial and its implications for early marsupial evolution; pp. 101-105 *i*n P. J. Currie, and E. H. Koster (eds.), Fourth Symposium on Mesozoic Terrestrial Ecosystems. Tyrrell Museum, Drumheller.

Fox, R. C., and B. G. Naylor. 1986. A new species of *Didelphodon* Marsh (Marsupialia) from the Upper Cretaceous of Alberta, Canada. Paleobiology and phylogeny. Neues Jahrbuch für Geologie und Paläontologie Abhandlungen 172:357-380.

Fox, R. C., and B. G. Naylor. 2006. Stagodontid marsupials from the Late Cretaceous of Canada and their systematic and functional implications. Acta Palaentologica Polonica 51:13-36.

Fox, R. C., C. S. Scott, and H. N. Bryant. 2007. A new, unusual therian mammal from the Upper Cretaceous of Saskatchewan, Canada. Cretaceous Research 28:821-829.

Gazin, C. L. 1952. The Lower Eocene Knight Formation of western Wyoming and its mammalian faunas. Smithsonian Miscellaneous Collection 131:1-82.

Gingerich, P. D., and T. Smith. 2006. Paleocene-Eocene land mammals from three new latest Clarkforkian and earliest Wasatchian wash sites at Polecat Bench in the northern Bighorn Basin, Wyoming. Contributions from the Museum of Paleontology. University of Michigan 31:245-303.

Goin, F. J., A. M. Candela, and C. d. Muizon. 2003. The affinities of *Roberthoffstetteria nationalgeograhica* (Marsupialia) and the origin of the polydolopine molar pattern. Journal of Vertebrate Paleontology 23:869-876.

Gordon, C. L. 2003. A first look at estimating body size in dentally conservative marsupials. Journal of Mammalian Evolution 10:1-21.

Green, M., and J. E. Martin. 1976. *Peratherium* (Marsupialia: Didelphidae) from the Oligocene and Miocene of South Dakota; pp. 155-168 *i*n C. S. Churcher (ed.), Athlon: Essays on Palaeontology in Honour of Loris Shano Russell. Royal Ontario Museum of Life Sciences, Ottawa.

Gregory, W. K., and G. G. Simpson. 1926. Cretaceous mammal skulls from Mongolia. American Museum Novitates 225:1-20.

Hershkovitz, P. 1982. The staggered marsupial lower third Incisor (I3). Géobios 6:191-200.

Hershkovitz, P. 1995. The staggered marsupial third lower incisor: Hallmark of cohort Didelphimorphia, and description of a new genus and species with staggered i3 from the Albian (Lower Cretaceous) of Texas. Bonner Zoologische Beitrage 45:153-169.

Horovitz, I., T. Martin, J. I. Bloch, S. Ladevèze, C. Kurz, and M. R. Sánchez-Villagra. 2009. Cranial Anatomy of the earliest Marsupials and the origin of opossums. PLoS ONE 4:e8278.

Horovitz, I., and M. R. Sánchez-Villagra. 2003. A morphological analysis of marsupial mammal higher-level phylogenetic relationships. Cladistics 19:181-112.

Hunter, J. P., R. E. Heinrich, and D. B. Weishampel. 2010. Mammals from the St. Mary River Formation (Upper Cretaceous), Montana. Journal of Vertebrate Paleontology 30:885-898.

Johanson, Z. 1995. New information concerning the Late Cretaceous marsupial *Albertatherium* Fox, 1971. Journal of Vertebrate Paleontology 14:595-602.

Johanson, Z. 1996a. New marsupial from the Fort Union Formation, Swain Quarry, Wyoming. Journal of Paleontology 70:1023-1031.

Johanson, Z. 1996b. Revision of the Late Cretaceous North American marsupial genus *Alphadon*. Palaeontographica Abteilung A 242:127-184.

Kielan-Jaworowska, Z. 1975. Preliminary descriptions of two new eutherian genera from the Late Cretaceous of Mongolia. Palaeontologia Polonica 33:5-16.

Kielan-Jaworowska, Z., and R. L. Cifelli. 2001. Primitive boreosphenidan mammal (?Deltatheroida) from the Early Cretaceous of Oklahoma. Acta Palaeontologica Polonica 46:377-391.

Kielan-Jaworowska, Z., R. L. Cifelli, and Z.-X. Luo. 2004. Mammals from the Age of Dinosaurs: Origins, evolution, and structure. 630 pp. Columbia University Press, New York.

Kielan-Jaworowska, Z., and D. Dashzeveg. 1989. Eutherian mammals from the Early Cretaceous of Mongolia. Zoologica Scripta 18:347-355.

Kielan-Jaworowska, Z., and L. A. Nessov. 1990. On the metatherian nature of Deltatheroida, a sister group of the Marsupialia. Lethaia 23:1-10.

Korth, W. W. 1994. Middle Tertiary marsupials (Mammalia) from North America. Journal of Paleontology 68:376-397.

Korth, W. W. 2008. Marsupialia; pp. 39-47 *i*n C. M. Janis, G. F. Gunnell, and M. D. Uhen (eds.), Evolution of Tertiary Mammals of North America, Vol. 2. Cambridge University Press, Cambridge.

Krause, D. W. 2001. Fossil molar from a Madagascar marsupial. Nature 412:497-498.

Krishtalka, L., and R. K. Stucky. 1983a. Paleocene and Eocene marsupials of North America. Annals of Carnegie Museum 52:229-263.

Krishtalka, L., and R. K. Stucky. 1983b. Revision of the Wind River faunas, early Eocene of Central Wyoming, Part 3. Marsupialia. Annals of Carnegie Museum 52:205-227.

Krishtalka, L., and R. K. Stucky. 1984. Middle Eocene marsupials from northeastern Utah and the mammalian fauna from Powder Wash. Annals of Carnegie Museum 53:31-45.

Ladeveze, S., and C. de Muizon. 2007. The auditory region of early Paleocene Pucadelphydae (Mammalia, Metatheria) from Tiupampa, Bolivia, with phylogenetic implications. Palaeontology 50:1123-1154.

Lambe, L. M. 1902. On vertebrates of the Mid-Cretaceous of the North West Territory-New genera and species form the Belly River Series (Mid-Cretaceous). Contributions to Canadian Palaeontology, Geological Survey of Canada 3:25-81.

Lillegraven, J. A. 1969. Latest Cretaceous mammals of the upper part of Edmonton Formation of Alberta, Canada, and review of Marsupial-Placental dichotomy in mammalian evolution. The University of Kansas Paleontological Contributions:1-122.

Lillegraven, J. A., and M. C. McKenna. 1986. Fossil mammals from the "Mesaverde" Formation (Late Cretaceous, Judithian) of the Bighorn and Wind River Basins, Wyoming, with definitions of Late Cretaceous North American Land Mammal "Ages". American Museum Novitates:1-68.

Lofgren, D. L. 1992. Upper premolar configuration of *Didelphodon vorax* (Mammlia, Marsupialia, Stagodontida). Journal of Paleontology 66:162-164.

Lofgren, D. L. 1995. The Bug Creek problem and the Cretaceous-Tertiary transition at McGuire Creek, Montana. University of California Publications in the Geological Sciences 140:1-185.

Lopatin, A. V., and A. O. Averianov. 2007. *Kielantherium*, a basal tribosphenic mammal from the Early Cretaceous of Mongolia, with new data on the aegialodontian dentition. Acta Palaeontologica Polonica 52:441-446.

Luo, Z.-X., Q. Ji, J. R. Wible, and C.-X. Yuan. 2003. An Early Cretaceous tribosphenic mammal and metatherian evolution. Science 302:1934-1940.

Luo, Z.-X., C.-X. Yuan, Q.-J. Meng, and Q. Ji. 2011. A Jurassic eutherian mammal and divergence of marsupials and placentals. Nature 476:442-445.

Luo, Z. X., Z. Kielan-Jaworowska, and R. L. Cifelli. 2002. In quest for a phylogeny of Mesozoic mammals. Acta Palaeontologica Polonica 47:1-78.

Marsh, O. C. 1889. Discovery of Cretaceous Mammalia. American Journal of Science 3:81-92.

Marshall, L. G., and C. de Muizon. 1988. The dawn of the age of mammals in South America. National Geographic Research 4:23-55.

Marshall, L. G., and C. de Muizon. 1995. Part II: The skull, *Pucadelphys andinus* (Marsupialia, Mammalia) from the early Paleocene of Bolivia; pp. 21-90 *i*n L. G. Marshall, C. d. Muizon, and D. Sigogneau-Russell (eds.), Memoires du Museum National d'Histoire Naturelle. Serie C., Sciences de la Terre.

Marshall, L. G., C. d. Muizon, and B. Sigé. 1983. Late Cretaceous mammals (Marsupialia) from Bolivia. Géobios 16:739-745.

Martin, J. E., J. A. Case, J. W. M. Jagt, A. S. Schulp, and E. W. A. Mulder. 2005. A new European marsupial indicates a Late Cretaceous high-latitude transatlantic dispersal route. Journal of Mammalian Evolution 12:495-511.

Matthew, W. D. 1916. A marsupial from the Belly River Cretaceous. With critical observations upon the affinities of the Cretaceous mammals. Bulletin of the American Museum of Natural History 35:477-500.

Matthew, W. D. 1937. Paleocene faunas of the San Juan Basin, New Mexico [edited by Walter Granger, William King Gregory, and Edwin Harris Colbert]. Transactions of the American Philosophical Society 30:510.

Matthew, W. D., and W. Granger. 1921. New genera of Paleocene mammals. American Museum Novitates 13:1-7.

McGrew, P. O. 1959. Marsupialia. Bulletin of the American Museum of Natural History 117:147-148.

McKenna, M. C. 1960. Fossil Mammalia from the early Wasatchian Four Mile local fauna, Eocene of northwest Colorado. University of California Publications in Geological Sciences 37:1-130.

Montellano, M. 1988. *Alphadon halleyi* (Didelphidae, Marsupialia) from the Two Medicine Formation (Late Cretaceous, Judithian) of Montana. Journal of Vertebrate Paleontology 8:378-382.

Montellano, M. 1992. Mammalian Fauna of the Judith River Formation (Late Cretaceous, Judithian), Northcentral Montana. University of California Publications in Geological Sciences 136:115 pp.

Nessov, L. A. 1982. Ancient mammals of the USSR. Ezhegodnik Vsesoyuznogo Paleontologicheskogo Obshchestva 35:228-243.

Nessov, L. A. 1985. Novyye mlyekopitayushchiye myela Kyzylkumov [New mammals from the Cretaceous of the Kyzylkum Desert]. Vyestnik Lyeningradskogo Univyersityeta Syeriya 7 17:8-18.

Nessov, L. A., D. Sigogneau, and D. E. Russell. 1994. A survey of Cretaceous tribosphenic mammals from middle Asia (Uzbekistan, Kazakhstan, and Tajikistan), of their geological setting, age and faunal environment. Palaeovertebrata 23:51-92.

Novacek, M. J., I. Ferrusqia-Villafranca, J. J. Flynn, A. Wyss, and M. A. Norell. 1991. Wasatchian (early Eocene) mammals and other vertebrates from Baja California, Mexico: the Loma Las Tetas de Cabra fauna. Bulletin of the American Museum of Natural History 208:1-88.

Novacek, M. J., G. W. Rougier, J. R. Wible, M. C. McKenna, D. Dashzeveg, and I. Horovitz. 1997. Epipubic bones in eutherian mammals from the Late Cretaceous of Mongolia. Nature 389:483-486.

Rigby, J. K., Jr., and D. L. Wolberg. 1987. The therian mammalian fauna (Campanian) of Quarry 1, Fossil Forest study area, San Juan Basin, New Mexico; pp. 51-80 *i*n J. E. Fassett, and J. K. Rigby, Jr. (eds.), The Cretaceous-Tertiary Boundary in the San Juan and Raton Basins, New Mexico and Colorado. Geological Society of America, Boulder, Colorado.

Rose, K. D. 2010. New Marsupial from the Early Eocene of Virginia. Journal of Paleontology 84:561-565.

Rougier, G. W., J. R. Wible, and M. J. Novacek. 1998. Implications of *Deltatheridium* specimens for early marsupial history. Nature 396:459-463.

Rougier, G. W., J. R. Wible, and M. J. Novacek. 2004. New Specimen of *Deltatheroides cretacicus* (Metatheria, Deltatheroida) from the Late Cretaceous of Mongolia. Bulletin of Carnegie Museum of Natural History, Vol. 36:245-266.

Russell, L. S. 1952. Cretaceous mammals of Alberta. Annual Report of the National Museum for the fiscal year 1950-1951 126:110-118.

Sahni, A. 1972. The vertebrate fauna of the Judith River Formation, Montana. Bulletin of the American Museum of Natural History 147:321-412.

Sánchez-Villagra, M., S. Ladeveze, I. Horovitz, T. E. Macrini, J. E. Martin, S. Morre-Fay, C. De Muizon, T. Schmeizle, and R. J. Asher. 2007. Exceptionally preserved North American Paleogene metatherians: Adaptations and discovery of a major gap in the opossum fossil record. Biological Letters 3:318-322.

Sigogneau-Russell, D., D. Dashzeveg, and D. E. Russell. 1992. Further data on *Prokennalestes* (Mammalia, Eutheria inc. sed.) from the Early Cretaceous of Mongolia. Zoologica Scripta 21:205-209.

Simpson, G. G. 1927. Mammalian fauna of the Hell Creek Formation of Montana. American Museum Novitates:1-7.

Simpson, G. G. 1928. American Eocene didelphids. American Museum Novitates 307:1-7.

Slaughter, B. H. 1965. A therian from the Lower Cretaceous (Albian) of Texas. Postilla 93:1-18.

Slaughter, B. H. 1968. Earliest known marsupials. Science 162:254-255.

Smith Woodward, A. 1916. On a mammalian mandible (*Cimolestes cutleri*) from an Upper Cretaceous formation in Alberta, Canada. Zoological Society of London Proceedings 158:525-528.

Springer, M. S., J. A. W. Kirsch, and J. A. Case. 1997. The chronicle of marsupial evolution; pp. 129-161 *i*n T. J. Givnish, and K. J. Sytsma (eds.), Molecular Evolution and Adaptative Radiation. Cambridge University Press, New York.

Stock, C. 1936. Sespe Eocene didelphids. Proceedings of the National Academy of Sciences (USA) 22:122-124.

Storer, J. E. 1991. The mammals of the Gryde local fauna, Frenchman Formation (Maastrichtian: Lancian), Saskatchewan. Journal of Vertebrate Paleontology 11:350-369.

Strait, S. G. 2001. New Wa-0 mammalian fauna from Castle Gardents in the southeastern Bighorn Basin. University of Michigan Papers on Paleontology 33:127-143.

Szalay, F. S., and B. A. Trofimov. 1996. The mongolian Late Cretaceous *Asiatherium*, and the early phylogeny and Paleobiogeography of Metatheria. Journal of Vertebrate Paleontology 16:474-509.

Trofimov, B. A., and F. S. Szalay. 1993. New group of Asiatic marsupials (Order Asiadelphia) from the Late Cretaceous of Mongolia. Journal of Vertebrate Paleontology 13.

Troxell, E. L. 1923. A new marsupial. American Journal of Science 5:507-510.

Vullo, R., E. Gheerbrant, C. d. Muizon, and D. Néraudeau. 2009. The oldest modern therian mammal from Europe and its bearing on stem marsupial paleobiogeography. Proceedings of the National Academy of Science (USA) 106:19910-19915.

Wible, J. R., G. W. Rougier, M. J. Novacek, and R. J. Asher. 2009. The eutherian mammal *Maelestes gobiensis* from the Late Cretaceous of Mongolia and the phylogeny of Cretaceous Eutheria. Bulletin of the American Museum of Natural History 327:1-123.

Williamson, T. E., S. L. Brusatte, T. D. Carr, A. Weil, and B. R. Standhardt. 2012. The phylogeny and evolution of Cretaceous-Paleogene metatherians: New cladistic analysis and description of new early Paleocene specimens from the Nacimiento Formation, New Mexico. Journal of Systematic Palaeontology 10:625-651.

Williamson, T. E., and D. L. Lofgren. 2014. Late Paleocene (Tiffanian) metatherians from the Goler Formation, California. Journal of Vertebrate Paleontology 34:477-482.

Williamson, T. E., and L. Taylor. 2011. New species of *Peradectes* and *Swaindelphys* (Mammalia; Metatheria) from the early Paleocene (Torrejonian) Nacimiento Formation, San Juan Basin, New Mexico. Palaeontologia Electronica 14:23A: 16 p.

Wilson, G. P., and J. A. Riedel. 2010. New specimen reveals deltatheroidan affinities of the North American Late Cretaceous Mammal Nanocuris. Journal of Vertebrate Paleontology 30:872 - 884.
