# Supplementary material for: The origin and early evolution of metatherian mammals: the Cretaceous record
Source: Zookeys. 2014 Dec 17;(465):1–76. doi: 10.3897/zookeys.465.8178 (PMC4284630; doi:10.3897/zookeys.465.8178)
Supplement: Supplementary material 3 — Characters in common on the most parsimonious trees diagnosing the nodes on the strict consensus tree in Figure 6. [file zookeys-465-001-s003.docx]

Node 1 :

All trees:

No synapomorphies

Node 2 :

All trees:

Char. 30: 1 --> 0

Char. 36: 1 --> 0

Char. 37: 1 --> 0

Char. 57: 1 --> 0

Node 3 :

All trees:

Char. 12: 0 --> 2

Char. 13: 0 --> 1

Char. 23: 0 --> 1

Char. 38: 0 --> 1

Char. 66: 0 --> 1

Char. 81: 0 --> 1

Some trees:

Char. 46: 0 --> 1

Char. 48: 1 --> 0

Char. 65: 0 --> 1

Node 4 :

All trees:

Char. 49: 1 --> 0

Char. 61: 1 --> 0

Char. 62: 1 --> 0

Char. 64: 0 --> 1

Char. 78: 1 --> 0

Some trees:

Char. 60: 0 --> 1

Char. 68: 1 --> 0

Node 5 :

All trees:

Char. 22: 1 --> 0

Some trees:

Char. 50: 0 --> 1

Node 6 :

All trees:

Char. 17: 1 --> 0

Char. 39: 1 --> 0

Some trees:

Char. 81: 0 --> 1

Node 7 :

All trees:

Char. 34: 0 --> 1

Some trees:

Char. 20: 1 --> 0

Char. 36: 0 --> 1

Node 8 :

All trees:

Char. 39: 1 --> 0

Char. 58: 1 --> 0

Char. 59: 0 --> 1

Char. 70: 1 --> 0

Char. 72: 0 --> 1

Some trees:

Char. 5: 0 --> 1

Char. 20: 1 --> 0

Char. 50: 0 --> 1

Node 9 :

All trees:

Char. 51: 0 --> 1

Char. 57: 0 --> 1

Char. 69: 0 --> 1

Char. 71: 0 --> 1

Some trees:

Char. 43: 2 --> 0

Node 10 :

All trees:

Char. 61: 1 --> 2

Some trees:

Char. 19: 3 --> 1

Char. 21: 0 --> 1

Char. 37: 0 --> 1

Char. 40: 0 --> 1

Char. 41: 0 --> 1

Char. 46: 0 --> 01

Char. 60: 0 --> 01

Char. 64: 0 --> 01

Node 11 :

All trees:

Char. 81: 01 --> 0

Some trees:

Char. 24: 1 --> 0

Char. 32: 0 --> 1

Char. 38: 0 --> 1

Char. 47: 0 --> 1

Node 12 :

Some trees:

Char. 24: 1 --> 0

Char. 30: 0 --> 1

Char. 32: 0 --> 1

Char. 34: 1 --> 0

Char. 43: 02 --> 0

Node 13 :

All trees:

Char. 61: 2 --> 1

Some trees:

Char. 24: 1 --> 0

Char. 32: 0 --> 1

Char. 43: 0 --> 2

Char. 48: 0 --> 1

Char. 81: 01 --> 1

Node 14 :

All trees:

Char. 60: 0 --> 1

Char. 70: 0 --> 1

Some trees:

Char. 41: 2 --> 1

Char. 79: 0 --> 1

Node 15 :

All trees:

Char. 15: 0 --> 1

Node 16 :

Some trees:

Char. 61: 2 --> 1

Char. 81: 0 --> 1

Node 17 :

All trees:

Char. 40: 1 --> 2

Char. 41: 12 --> 2

Some trees:

Char. 19: 1 --> 3

Char. 22: 1 --> 2

Char. 32: 01 --> 0

Char. 37: 1 --> 0

Char. 47: 01 --> 1

Char. 65: 0 --> 1

Char. 66: 0 --> 1

Char. 74: 0 --> 1

Char. 81: 01 --> 2

Node 18 :

All trees:

Char. 34: 1 --> 0

Node 19 :

All trees:

Char. 40: 1 --> 2

Char. 48: 0 --> 1

Char. 79: 0 --> 1

Some trees:

Char. 34: 1 --> 0

Char. 43: 02 --> 0

Node 20 :

All trees:

Char. 45: 0 --> 1

Some trees:

Char. 40: 2 --> 3

Char. 58: 0 --> 1

Node 21 :

All trees:

Char. 31: 0 --> 1

Char. 35: 0 --> 1

Some trees:

Char. 22: 1 --> 0

Char. 32: 0 --> 1

Node 22 :

All trees:

Char. 26: 0 --> 1

Char. 28: 0 --> 1

Node 23 :

All trees:

Char. 20: 0 --> 1

Char. 35: 0 --> 1

Some trees:

Char. 8: 0 --> 1

Char. 80: 0 --> 1

Node 24 :

All trees:

Char. 45: 0 --> 1

Char. 68: 2 --> 1

Node 25 :

All trees:

Char. 75: 0 --> 1

Node 26 :

All trees:

Char. 27: 0 --> 1

Char. 39: 0 --> 1

Char. 42: 0 --> 2

Char. 52: 0 --> 1

Char. 57: 0 --> 2

Some trees:

Char. 32: 0 --> 1

Char. 43: 0 --> 2

Node 27 :

All trees:

Char. 24: 1 --> 0

Char. 34: 0 --> 1

Char. 47: 0 --> 1

Char. 53: 0 --> 1

Char. 59: 1 --> 2

Char. 60: 0 --> 1

Char. 76: 0 --> 1

Char. 79: 1 --> 2

Char. 82: 1 --> 0

Node 28 :

All trees:

Char. 7: 2 --> 1

Char. 81: 1 --> 0

Some trees:

Char. 8: 1 --> 0

Node 29 :

All trees:

Char. 32: 0 --> 1

Char. 36: 1 --> 0

Some trees:

Char. 21: 1 --> 0

Char. 45: 0 --> 1

Char. 60: 1 --> 0

Node 30 :

All trees:

Char. 38: 0 --> 1

Node 31 :

All trees:

Char. 40: 2 --> 1

Some trees:

Char. 41: 2 --> 1

Node 32 :

All trees:

Char. 59: 1 --> 0

Char. 61: 2 --> 1

Char. 62: 2 --> 1

Node 33 :

All trees:

Char. 42: 0 --> 2

Char. 43: 0 --> 2

Char. 81: 0 --> 1

Some trees:

Char. 41: 1 --> 2

Node 34 :

All trees:

Char. 60: 1 --> 0

Char. 68: 1 --> 0

Node 35 :

All trees:

Char. 23: 0 --> 1

Char. 47: 0 --> 1

Char. 57: 0 --> 2

Some trees:

Char. 19: 1 --> 3

Char. 22: 1 --> 0

Char. 33: 0 --> 1

Node 36 :

All trees:

Char. 34: 0 --> 1

Char. 52: 0 --> 1

Some trees:

Char. 21: 1 --> 0

Node 37 :

All trees:

Char. 33: 1 --> 2

Some trees:

Char. 19: 3 --> 0

Node 38 :

All trees:

Char. 30: 0 --> 1

Char. 44: 1 --> 0

Char. 48: 1 --> 0

Node 39 :

All trees:

Char. 46: 1 --> 0

Char. 47: 1 --> 0

Char. 61: 2 --> 1

Char. 68: 2 --> 1

Node 40 :

All trees:

Char. 15: 0 --> 1

Char. 38: 0 --> 1

Char. 41: 2 --> 1

Node 41 :

All trees:

Char. 39: 0 --> 1

Char. 62: 2 --> 1

Node 42 :

All trees:

Char. 59: 1 --> 2

Char. 61: 2 --> 1

Node 43 :

All trees:

Char. 19: 0 --> 1

Char. 32: 0 --> 1

Char. 34: 1 --> 0

Char. 40: 1 --> 0

Char. 57: 2 --> 1

Node 44 :

All trees:

Char. 33: 2 --> 1

Char. 81: 1 --> 2
